# Companion Diagnostics in Clinical Therapy: Current Applications and Future Directions

**DOI:** 10.1002/mco2.70638

**Published:** 2026-03-01

**Authors:** Yuesong Wu, Rou Xue, Xiangwen Luo, Jiangnan Liao, Zongbo Zhang, Jinhai Deng, Teng Liu, Xin Li, Zhe‐Sheng Chen, Mingzhu Yin

**Affiliations:** ^1^ Clinical Research Center (CRC) Medical Pathology Center (MPC) Cancer Early Detection and Treatment Center (CEDTC) and Translational Medicine Research Center (TMRC) Chongqing University Three Gorges Hospital, Chongqing University Wanzhou District Chongqing China; ^2^ Chongqing Technical Innovation Center for Quality Evaluation and Identification of Authentic Medicinal Herbs Wanzhou District Chongqing China; ^3^ School of Medicine Chongqing University Chongqing University Shapingba District Chongqing China; ^4^ Richard Dimbleby Department of Cancer Research Comprehensive Cancer Centre, Kings College London London United Kingdom; ^5^ Chongqing University Three Gorges Hospital & Academy for Advanced interdisciplinary Technology, CQU ‐ Ferenc Krausz Nobel Laureate Scientific Workstation Chongqing China; ^6^ Department of Pharmaceutical Sciences College of Pharmacy and Health Sciences St. John's University Queens New York USA; ^7^ Institute of Advanced Interdisciplinary Studies Chongqing University Chongqing China

**Keywords:** companion diagnostics, in vitro diagnostics, next‐generation sequencing, oncology treatment, personalized medicine, precision medicine

## Abstract

Companion diagnostics (CDx) plays a pivotal role in precision medicine by enabling personalized treatment plans based on individual biomarker profiles. This approach can enhance therapeutic efficacy in selected indications and may reduce healthcare expenditures. Particularly in oncology, precision targeted therapies targeting pathways such as EGFR, HER2, and programmed death‐1/programmed death‐ligand 1 have established robust models for biomarker‐driven treatment. However, rapid advancements in diagnostic technologies, expanding application scopes, and increasingly complex mechanisms of biomarker resistance are presenting new challenges for CDx. This review comprehensively examines the evolving regulatory landscape, current clinical applications across various solid tumors and hematologic malignancies, and diverse methodological platforms ranging from next‐generation sequencing and immunohistochemistry to emerging liquid biopsies and point‐of‐care testing. It also delves into persistent barriers in CDx development, including tumor heterogeneity, test standardization, trade‐offs between tissue biopsy and liquid biopsy, and the economic complexities of codevelopment and reimbursement mechanisms. By synthesizing existing knowledge and projecting future trends, this paper serves as a valuable resource for researchers, regulators, and clinicians. It provides critical insights to guide the synergistic development of drugs and diagnostics, paving the way for their integration into a more dynamic, artificial intelligence‐enhanced, and multiomics‐driven healthcare ecosystem.

## Graphical abstract

Companion diagnostics use biomarker‐based assays to stratify patients for precision therapies, improving outcomes while reducing ineffective treatment and side effects. This review summarizes the evolution, regulatory landscape, clinical applications, and technological platforms of CDx, highlighting their role in biomarker‐driven precision therapy. It outlines current challenges and emphasizes future directions, including multiomics integration, AI‐enabled interpretation, and dynamic, next‐generation CDx development. Created with BioRender.

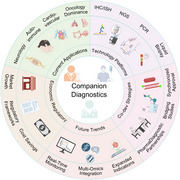

## 1 Introduction

Precision medicine is a medical paradigm characterized by the use of individual molecular features to tailor corresponding treatment strategies, whether for timely disease prevention, identification of disease susceptibility, or optimization of treatment plans [1, 2, 3]. In 1998, the discovery of the HER2 protein encoded by the ERBB2 gene as a key target led to the approval of trastuzumab and its companion test HercepTest for the treatment of HER2‐positive breast cancer [4, 5]. This not only established the cornerstone of precision treatment but also highlighted the core role of reliable biomarker testing in targeted therapy. Companion diagnostics (CDx) is a diagnostic paradigm that provides clinicians with crucial evidence for selecting the most appropriate treatment and predicting treatment outcomes by detecting specific biomarkers such as genes, proteins, or metabolites in patients. In highly heterogeneous and complex diseases like cancer, CDx assays have become an indispensable bridge connecting molecular drivers with clinical benefits.

Advances in precision medicine have accelerated the need for the simultaneous development of companion or complementary in vitro diagnostics (IVDs) in drug clinical trials. In 2014, the United States Food and Drug Administration (US FDA) formally defined CDx as IVD devices that furnish crucial information necessary for the safe and effective administration of corresponding drugs or biological products [6]. In parallel, complementary diagnostics are designed to assist in evaluating the benefit–risk profile of therapeutic interventions, particularly in settings where clinically meaningful differences in treatment benefit exist. Essential information from complementary IVDs is typically incorporated into the labeling of therapeutic products [7]. Before 2015, complementary diagnostics pertained to tests aimed at enhancing disease management, early diagnosis, patient risk stratification, and drug monitoring, but they did not necessitate a regulatory linkage to a specific therapeutic [8]. Historically, the first US FDA‐approved CDx was HercepTest for trastuzumab in 1998 [9], followed by the first complementary diagnostic, the programmed death‐ligand 1 (PD‐L1) IHC assay for nivolumab in 2015 [10]. By approving complementary diagnostics, the US FDA demonstrated its commitment to ensuring that patients are not excluded from immunotherapy, even when treatment benefit correlates with high PD‐L1 expression in tumors [11].

Currently, the majority of CDx and complementary diagnostics are applied in oncology, reflecting the proliferation of targeted therapies and the increasingly sophisticated understanding of cancer‐related genetic and immunologic pathways. In the diagnostic process, these assays typically measure the expression of the therapeutic target in tumor tissue or identify mutations in the gene corresponding to the therapeutic target. This establishes a clear link between the biomarker measured by the diagnostic test and the mechanism of action of the therapeutic agent [12]. In contrast to CDx, complementary diagnostics represent a relatively recent addition to the toolkit. While not mandatory for the safe and effective application of a therapeutic agent, they can provide valuable assistance to physicians in making benefit–risk assessments regarding therapeutic usage [13]. However, the rapid expansion of biomarker–drug pairs and testing platforms has also raised practical challenges in assay selection, validation, standardization, and implementation across clinical settings, underscoring the need for an updated and integrative review that connects clinical applications with technology platforms and real‐world considerations.

This review provides a comprehensive overview of the development history of CDx and the changes in its regulatory policies and summarizes the evolution process of CDx based on biomarkers approved by the US FDA. Subsequently, the article delves into the specific applications of CDx based on popular biomarkers in various disease types. Then, the article details the advantages and disadvantages of the current mainstream CDx detection technology platforms and focuses on the development of emerging technology platforms, including liquid biopsy, imaging‐based CDx assays, and point‐of‐care testing (POCT). Finally, the article offers insights into economic considerations and regulatory frameworks worldwide and summarizes the future development direction of CDx. In summary, a comprehensive review of CDx not only provides a scientific basis for individualized clinical medication but also lays a solid foundation for the innovation and practice of interdisciplinary diagnosis and treatment models in the future.

## 2 The Evolution and Regulatory Landscape of CDx

The evolution of CDx has paralleled the rise of precision medicine, which identifies biological information such as RNA/DNA and proteins to stratify patients and target those most likely to respond to a specific treatment [14]. With a deeper understanding of molecules, CDx emerged as a complementary tool to ensure the accurate identification of specific drug‐suitable patients [15, 16]. Consequently, pharmaceuticals are now being designed specifically for well‐defined subsets of patients, with CDx playing a pivotal role in this process [11]. However, a significant challenge in the implementation of precision medicine is that many patients do not receive the necessary tests in a timely manner, even though these tests are readily available [17]. To ensure that the outcomes of precision medicine can truly benefit patients, the regulatory framework and pathways guiding CDx have undergone significant evolution, as discussed below.

### 2.1 Historical Perspective: From Codevelopment to Regulatory Frameworks

**FIGURE 1:**
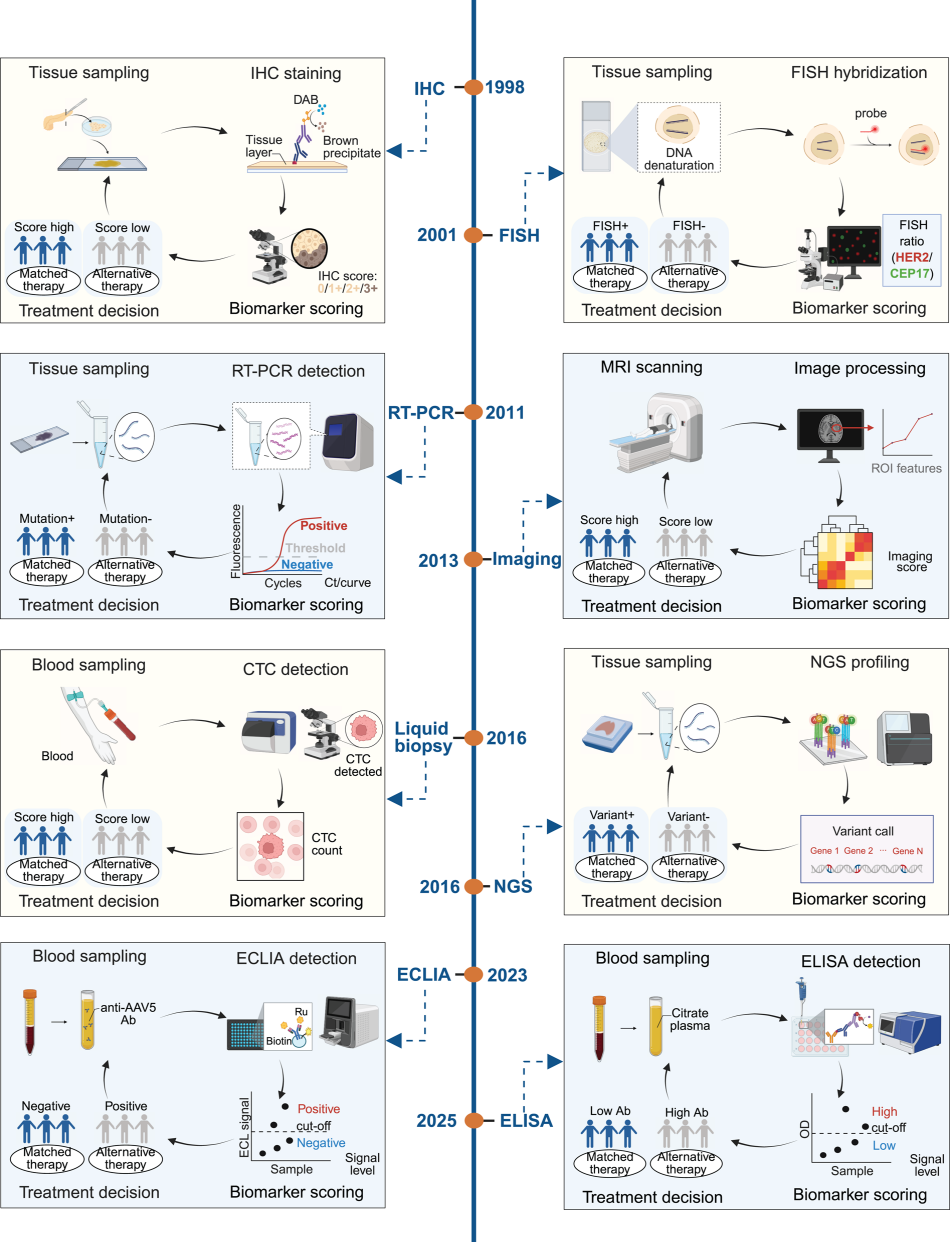
Analytical approaches used in CDx development. The graph systematically shows the CDx evolution from tissue level detection (e.g., IHC in 1998 and FISH in 2001) to the molecular level (RT‐PCR in 2011 and NGS in 2016), imaging histology (imaging‐based approaches in 2013), and minimally invasive liquid biopsy (CTC assay in 2016 and ECLIA/ELISA in 2023–2025). Created with BioRender.

Approximately two decades ago, the CDx field witnessed a significant milestone when the first predictive biomarker, the HER2 protein, along with its associated immunohistochemical assay known as HercepTest, gained approval from the US FDA [18, 19]. In 2006, the concept of CDx was first introduced [20]. Over time, an increasingly widespread consensus emerged among researchers, emphasizing the importance of subjecting biomarker studies within the diagnostic industry to the same level of rigor as therapeutic trials conducted within pharmaceutical companies. The number of US FDA‐approved CDx assays has steadily increased, reaching 34 approved in 2017, 44 in 2021, and a remarkable 188 as of 2025. Regulatory requirements mandating that CDx meets minimum performance criteria are in place in numerous countries, including the US, the European Union (EU), and Japan. A significant milestone occurred with the introduction of the first US FDA‐approved assay based on NGS, namely, the FoundationFocus CDx BRCA assay, designed to detect BRCA1 and BRCA2 alterations in ovarian cancer patients [6]. In 2017, the US FDA granted approval to FoundationOne CDx as the first comprehensive genomic profiling assay for all solid tumors, encompassing multiple CDx assays. The number of US FDA‐approved NGS‐based CDx assays increased from 7 in February 2021, 33 in August 2023 to 85 in March 2025. Various analytical approaches underpin CDx development, encompassing techniques such as PCR [21], IHC [22], Sanger sequencing [23], liquid biopsy, in situ hybridization (ISH), magnetic resonance imaging (MRI), and NGS [24]. CDx has evolved from single‐marker testing to multimarker and high‐throughput platforms (Figure 1). Next‐generation CDx enables high‐throughput, simultaneous assessment of multiple biomarkers, shifting diagnostics from single‐marker tests to integrated multibiomarker approaches [25, 26]. Advances in genomic and sequencing technologies have clarified oncogenic mechanisms and facilitated the discovery of more accurate predictive markers and targeted therapies [27].

Recognizing the pivotal role of CDx assays in patient management, it is only logical that several other countries, including the United States, the EU, China, Korea, and Japan, have adopted similarly stringent requirements in their regulatory frameworks [28]. In 2014, the US FDA first published guidelines defining in vitro CDx devices, with subsequent refinements in 2016, 2020, and 2023 [29]. The 2023 guidance document “Oncology Drug Products Used with Certain In Vitro Diagnostic Tests: Pilot Program” announced and described the US FDA's voluntary pilot program for certain CDER‐regulated oncology drug products used in conjunction with specific IVD tests [30]. In May 2022, new regulations governing the market approval of CDx in the EU came into full effect. Under the new regulation, CDx will be categorized as either high individual risk or moderate public health risk, necessitating a conformity assessment by a notified body (NB). Furthermore, it has been suggested that evidence of the device's clinical utility for its intended purpose should be a prerequisite for CDx [31]. The introduction of Regulation (EU) 2017/746 on IVD medical devices mandates that medicines regulatory authorities be consulted during the review of CDx conformity assessments, providing an opportunity for more consistent and transparent information on CDx to be disseminated [32]. Anticipated consequences include potential obstacles to innovation activities within small diagnostic companies and healthcare institutions. The primary driving factors here revolve around the potential for large diagnostic companies to establish monopolies and for major pharmaceutical companies to compete in markets where numerous products are available. To achieve a successful and cost‐effective personalized healthcare strategy that benefits all European patients, addressing challenges related to effective market access for CDx must be a priority for most member states [33]. While the principles, procedures, and requirements for drug approvals may diverge between the US FDA and the European Medicines Agency (EMA), there are ongoing efforts to harmonize these processes, simplifying global drug development endeavors [34]. In addition, Health Canada, the Therapeutic Goods Administration of Australia, and the Health Sciences Authority of Singapore have jointly formed a comprehensive global surveillance framework for CDx.

### 2.2 Key Regulatory Pathways and Guidelines for CDx Approval

Regulatory bodies are now implementing more rigorous regulations and are in the process of formulating or revising guidelines [35]. Table S1 enumerates the major differences in CDx regulation across the US FDA, EMA, National Medical Products Administration (NMPA) of China, Ministry of Food and Drug Safety (MFDS) of Korean, and Pharmaceuticals and Medical Devices Agency (PMDA) of Japan. The US FDA regulates CDx as medical devices, with the vast majority classified as Class III devices. Based on the risk level, they undergo premarket approval (PMA), 510(k) premarket notification, or de novo regulatory pathways, with PMA being the most stringent [36]. The US FDA guidance emphasizes three primary pathways for CDx development: codevelopment, bridging, and follow‐on. Codevelopment integrates the CDx into clinical trials with the therapeutic agent, allowing simultaneous approval, such as crizotinib and the Vysis ALK Break Apart FISH Probe Kit in 2011 [37]. Bridging involves retrospective validation of a CDx against the Clinical Trial Assay used in pivotal studies, such as trastuzumab with HercepTest [38]. Follow‐on CDx are developed after drug approval when original trial samples are unavailable, demonstrating analytical equivalence to an existing approved CDx, as with PathVysion HER2 DNA Probe Kit and Dako Cytomation HER2 FISH PharmD [39]. Recent US FDA guidance, including the 2023 document on oncology drug products used with IVD tests, further clarifies voluntary pilot programs and collaborative approaches to CDx development [40].

The EU and other nations that have implemented CDx regulations represent a significant stride toward enhancing patient safety. In 2017, the EU In Vitro Diagnostic Medical Devices Regulation classifies CDx as Class C high‐risk devices, requiring joint oversight by the EMA and NBs. Whether conducted using commercial test kits or by qualified laboratories, testing must demonstrate consistency between results obtained using the method employed in pivotal clinical trials and the method intended for postapproval use. Additionally, a literature review related to the selected biomarkers and their detection methods must be submitted to support their clinical and scientific validity [41]. CDx in MFDS belong to medium‐to‐high risk IVDs (Class 2–3) and require coregulation based on their relationship with drugs. Newly developed CDx devices must submit complete analytical and clinical data, while those based on existing approved products may follow a simplified pathway. All CDx devices must demonstrate equivalence to the test methods used in drug clinical trials and play a pivotal role in drug approval [42]. In Japan, CDx regulation is handled by the PMDA. During the early stages, the PMDA conducts strategic consultation and evaluation, assessing the quality, performance, and safety of CDx and corresponding drugs. These are then approved for market entry through either conditional approval or full approval [43]. China has introduced a series of regulations to provide a clear clinical evaluation pathway for the market launch of CDx products. The NMPA encourages the simultaneous development and submission of drugs and CDx tests. The drug review department and the medical device technical review department will conduct coordinated reviews for drugs and CDx reagents developed in tandem. This determination ensures the safety and efficacy of both products when used in conjunction, thereby demonstrating their potential clinical utility and ultimately benefiting patients upon their market introduction.

Overall, CDx requires rigorous validation of analytical performance and clinical effectiveness, alongside simultaneous approval and coordinated assessment with corresponding therapeutic drugs. These global regulatory guidelines aim to optimize CDx development processes, enhance approval collaboration, and ultimately improve clinical outcomes for patients.

### 2.3 Global Regulatory Harmonization and Challenges

As previously mentioned, there are significant variations in the approval of CDx globally, posing considerable challenges for pharmaceutical companies, regulatory agencies, and healthcare systems. Addressing these challenges and advancing precision medicine requires concerted efforts across multiple fronts. The early integration and global deployment of CDx at the strategic level are crucial. By comparing approval timelines for targeted therapies with and without CDx in Japan, it becomes evident that the early integration of CDx into drug development, coupled with a global development strategy, can significantly accelerate the overall timeline for oncology drug approval, thereby enabling patients to access optimal treatment options more rapidly [44]. This suggests that incorporating CDx into the early stages of drug development and formulating a global development strategy is a key driver of shortening the time‐to‐market of new drugs and enabling patients to benefit faster. Furthermore, Kukk et al. indicate that promoting personalized drugs and their corresponding CDx in the UK requires more than just technological advancement [45]. For instance, drugs and CDx assays must be launched in tandem, timing must be strategically aligned, and coordination among hospitals, insurance companies, and other stakeholders is essential for the entire system to function effectively. Addressing the challenges associated with implementing precision medicine in a nation like Brazil necessitates the collaboration of experts to tackle issues within an oncological context. These challenges encompass intricate regulatory hurdles, limited human and technical resources, and the complexities of a two‐tiered healthcare delivery system [46].

The journey to establish a validated CDx is marked by numerous challenges spanning various aspects, including biomarker discovery, the concurrent development of drugs and CDx tests, economic considerations, and regulatory compliance [47]. Therefore, it is necessary to continuously improve the measurement techniques of biomarkers and the corresponding analysis methods, while identifying and validating these biomarkers. Significant challenges encompass the development of robust procedures for the reliable handling of formalin‐fixed paraffin‐embedded samples, DNA recovery, analysis of DNA fragments, and the identification of highly sensitive detection methods [48]. Furthermore, the rapid pace of innovation in both drug development and diagnostic test creation presents a formidable obstacle in the codevelopment of drug–CDx. It is imperative that pharmaceutical and diagnostics collaborations commence early in the drug development process, with a simultaneous emphasis on the availability of high‐quality biological samples, which are pivotal for the successful development of a diagnostic test [49].

Validating a CDx involves a substantial investment by the diagnostic company, which may not necessarily yield a profitable return upon market entry. It is necessary to establish a value‐oriented incentive mechanism to promote the discovery and development of CDx testing methods with significant medical value, thereby meeting the growing demand for personalized drugs. The current intellectual property regulations offer very limited protection to most diagnostic test manufacturers. Therefore, determining an appropriate pricing strategy remains a challenge for most high‐value medical diagnostic analyses, although this specific CDx has proven to be effective [50]. In the United States, US FDA‐regulated IVDs are subject to stringent regulations [51], mandating a rigorously controlled development process akin to that of their companion drugs. In contrast, within the EU, a drug may obtain formal approval from the EMA, while the diagnostic test often qualifies for manufacturer self‐certification under the EU IVD directive [52]. However, the rapid advancement of CDx is poised to surmount these challenges, facilitating their widespread adoption on a much broader scale [53]. As a result, both the US FDA and the EMA require CDx marker testing before the use of certain drugs. However, real cooperation between pharmaceutical and diagnostic companies is difficult because clinically relevant biomarkers are often developed in the later stages of drug validation. Additionally, drug development and diagnostics are typically viewed as distinct fields, thus contributing to the complexity of their integration [54, 55].

### 2.4 The Significance of Biomarkers in CDx

A biomarker is defined as a characteristic that is objectively measured and assessed to serve as an indicator of normal biological processes, pathological processes, or pharmacological responses to therapeutic interventions [56]. In the context of CDx, biomarker discovery emerges as a pivotal step, significantly impacting clinical outcomes and accelerating drug development [57]. Clinically, biomarkers can be categorized as diagnostic, prognostic, predictive, or pharmacodynamic markers, serving critical functions in disease diagnosis, staging, and therapy selection [58]. A potential clinical biomarker should meet the criteria of reproducibility, specificity, and sensitivity [59]. The rapid advancement of molecular biology and laboratory technology has facilitated the identification of technically advanced biomarkers and the development of powerful CDx. These molecular biomarkers are identified across multiple omics levels, encompassing the genome, epigenome, transcriptome, proteome, metabolome, and lipidome.

Expanding upon these biomarkers, CDx devices play a pivotal role in aiding clinicians in making precise therapy decisions, thus garnering significant commercial and scientific interest [60, 61]. The ultimate objective of CDx testing is to furnish information that informs the clinical decision‐making process concerning medical treatments while enhancing treatment safety and efficacy [62]. CDx detection methods have been meticulously designed to align with specific therapies. They can operate on cells, or on specific molecules related to the disease process, or conduct analyses of the entire genes and/or genomes [63]. As of March 5, 2025, there are a total of 188 US FDA‐approved CDx assays, associated with 45 distinct molecular biomarkers (Table 1). These accompanying biomarkers have the potential to lead to the development of safer and more effective drugs, reduce the cost of clinical trials, and enhance the safety of the drugs.

**TABLE 1:** Representative US FDA‐approved CDx assays with respect to various biomarkers.

Biomarker	Drug therapy	Device name	Device manufacturer	Indication	Date
Liver iron concentration imaging	Exjade	FerriScan	Resonance Health Analysis Services Pty Ltd	Nontransfusion‐dependent thalassemia	01/23/2013
KIT	Gleevec	KIT D816V Assay	ARUP Laboratories, Inc.	ASM	12/18/2015
PDGFRB	Gleevec	PDGFRB FISH Assay	ARUP Laboratories, Inc.	MSM	12/18/2015
TP53	Venclexta	Vysis CLL FISH Probe Kit	Abbott Molecular, Inc.	BCLL	04/11/2016
IDH2	Idhifa	Abbott RealTime IDH2	Abbott Molecular, Inc.	AML	08/01/2017
t(9;21) Philadelphia chromosome	Tasigna	MRDx BCR‐ABL Test	MolecularMD Corporation	CML	12/22/2017
FGFR3	Balversa	therascreen FGFR RGQ RT‐PCR Kit	QIAGEN Manchester Ltd.	UC	04/12/2019
FGFR2	Pemazyre	FoundationOne CDx	Foundation Medicine, Inc.	Cholangiocarcinoma	04/17/2020
Myriad HRD	Lynparza	Myriad myChoice CDx	Myriad Genetic Laboratories, Inc	Ovarian cancer	05/08/2020
HRRG	Lynparza	FoundationOne CDx	Foundation Medicine, Inc.	MCRP cancer	05/19/2020
TMB	Keytruda	FoundationOne CDx	Foundation Medicine, Inc.	Solid tumors	06/16/2020
EZH2	Tazverik	Cobas EZH2 Mutation Test	Roche Molecular Systems, Inc.	FLT	06/18/2020
BRCA1, BRCA2, ATM	Lynparza	FoundationOne Liquid CDx	Foundation Medicine, Inc.	mCRPC	11/06/2020
ALK	Lorbrena	Ventana ALK CDx Assay	Ventana Medical Systems, Inc.	NSCLC	03/03/2021
POMC, PCSK1, LEPR	Imcivree	POMC/PCSK1/LEPR CDx Panel	PreventionGenetics, LLC	Obesity	01/21/2022
pMMR proteins	Keytruda	Ventana MMR RxDx Panel	Ventana Medical Systems, Inc.	Endometrial carcinoma	06/16/2022
ERBB2	ENHERTU	Guardant360 CDx	Guardant Health, Inc.	NSCLC	08/11/2022
RET mutations	Retevmo	Oncomine Dx Target Test	Life Technologies Corporation	Medullary thyroid cancer	09/21/2022
HLA	Tecelra	SeCore CDx HLA Sequencing System	One Lambda, Inc.	Synovial sarcoma	11/28/2022
ROS1	Rozlytrek	FoundationOne Liquid CDx	Foundation Medicine, Inc.	NSCLC	12/22/2022
EGFR (HER1)	Iressa	FoundationOne Liquid CDx	Foundation Medicine, Inc.	NSCLC	12/19/2022
ESR1	Orserdu	Guardant360 CDx	Guardant Health, Inc.	Breast cancer	01/27/2023
Anti‐AAV5 Antibodies	ROCTAVIAN	AAV5 DetectCDx	ARUP Laboratories	Hemophilia A	06/29/2023
PDGFRA	AYVAKIT	Therascreen PDGFRA RGQ	QIAGEN GmbH	GST	06/29/2023
FLT3 (ITD/TDK)	VANFLYTA	LeukoStrat CDx FLT3	Invivoscribe Technologies, Inc.	AML	07/20/2023
IDH1	Tibsovo	Abbott RealTime IDH1	Abbott Molecular, Inc.	Myelodysplastic syndromes	10/24/2023
PD‐L1	Keytruda	PD‐L1 IHC 22C3 pharmDx	Dako North America, Inc.	GEJ	11/07/2023
AAVRh74var capsid neutralizing antibodies	BEQVEZ	nAbCyte Anti‐AAVRh74var HB‐FE Assay	Labcorp Drug Development	Hemophilia B	04/25/2024
MAGE‐A4	Tecelra	MAGE‐A4 IHC 1F9 pharmDx	Agilent Technologies, Inc.	Synovial sarcoma	08/01/2024
FOLR1	Elahere	Ventana FOLR1 (FOLR‐2.1)	Ventana Medical Systems, Inc.	EO, FT, or PP cancer	08/01/2024
NTRK1/2/3	Vitrakvi	TruSight Oncology Comprehensive	Illumina, Inc.	Solid tumors	08/21/2024
RET fusions	Retevmo	TruSight Oncology Comprehensive	Illumina, Inc.	NSCLC	08/21/2024
BRCA1 and BRCA2	AKEEGA	FoundationOne CDx	Foundation Medicine, Inc.	mCRPC	08/30/2024
IDH1, IDH2	VORANIGO	Oncomine Dx Target Test	Life Technologies Corporation	Astrocytoma and oligodendroglioma	09/18/2024
Claudin 18	VYLOY	VENTANA CLDN18 RxDx Assay	Ventana Medical Systems, Inc.	GEJ adenocarcinoma	10/18/2024
MSI‐high	Jemperli	MI Cancer Seek	Caris Life Sciences	Solid tumors	11/05/2024
Not MSI‐high	Keytruda, Lenvima	MI Cancer Seek	Caris Life Sciences	EC	11/05/2024
KRAS and NRAS	Vectibix	MI Cancer Seek	Caris Life Sciences	Colorectal cancer	11/05/2024
PIK3CA	Piqray	MI Cancer Seek	Caris Life Sciences	Breast cancer	11/05/2024
MET	Tepmetko	FoundationOne Liquid CDx	Foundation Medicine, Inc.	NSCLC	11/14/2024
dMMR proteins	Imfinzi	Ventana MMR RxDx Panel	Ventana Medical Systems, Inc.	EC	12/18/2024
KRAS	Lumakras	Therascreen KRAS RGQ PCR Kit	Qiagen Manchester, Ltd.	Colorectal cancer	01/16/2025
BRAF	Ojemda	FoundationOne CDx	Foundation Medicine, Inc.	Low‐grade glioma	01/16/2025

*Source*: https://www.fda.gov/medical‐devices/in‐vitro‐diagnostics, until March 5, 2025.

Abbreviations: AML, acute myelogenous leukemia; ASM, aggressive systemic mastocytosis; BCLL, B‐cell chronic lymphocytic leukemia; CML, chronic myeloid leukemia; dMMR, deficient mismatch repair; EC, endometrial carcinoma; EO, epithelial ovaria cancer; FLT, follicular lymphoma tumor; FT, fallopian tube cancer; GEJ, gastroesophageal junction; GST, gastrointestinal stromal tumors; HRRG, homologous recombination repair genes; MCRPC, metastatic castrate resistant prostate cancer; MSM, myelodysplastic syndrome/myeloproliferative; pMMR, proficient mismatch repair; PP, primary peritoneal cancer; UC, urothelial cancer.

NGS techniques play a crucial part in the exploration of novel biomarkers for CDx [64]. Genome‐wide mutational analyses have revealed numerous previously unrecognized cancer‐associated genes, such as EGFR, ALK, ROS1, KRAS, and FGFR1 in lung cancer [65]; KRAS, BRAF, PIK3CA, PTEN, and NRAS in colorectal cancer [66]; and HER2, PI3K, VEGF, and mTOR in breast cancer [67]. There is an accumulating body of evidence underscoring the importance of establishing a well‐defined, comprehensive biomarker strategy early in the drug development process, thereby increasing the likelihood of new therapeutic approvals [68]. In the future, the adoption of a comprehensive or multibiomarker approach is poised to become both desirable and cost effective, primarily due to the availability of multitarget therapies and the intricate mechanisms of therapy resistance, often involving multiple genes within cancer biological pathways [69].

## 3 Current Clinical Applications of CDx

The commercial application of CDx has long been dominated by oncology, obesity, and hematological malignancies, but in recent years, some companies have been accelerating expansion into areas such as cardiovascular disease, neurological disorders, and autoimmune diseases. The development and clinical adoption of CDx tests in oncology therapy have advanced our comprehension of cancer biology among clinicians and addressed unmet medical needs for drug developers. Table 2 depicts the currently adopted cancer diagnostic approaches. Ongoing technological advancements are enabling the utilization of more sensitive, specific, and tissue‐sparing methods for the meaningful assessment of oncology biomarker levels and status [70]. This section summarizes CDx applications across major cancer types in accordance with the key biomarker categories outlined above. Additionally, we analyze the status and future trends of CDx in other diseases, such as COVID‐19, neuropsychiatric disorders, aortic valve diseases, and retinal vein occlusion (RVO) [71].

**TABLE 2:** Merits and demerits of currently applied approaches for cancer diagnostic.

Method	Definition	Advantage	Disadvantage	Applicable targets	Data source
IHC	Label target proteins in tissues or cells using specific antibodies	Detect specific protein; Routinely performed in hospital labs; Fast and cost effective	Limited sensitivity; Complex and time consuming to operate; Highly subjective results; Difficulty of multiple tests	PD‐L1 HER2 ALK MAGE‐A4	[72]
FISH	Fluorescent or colorimetric probes detect specific alterations	High sensitivity and specificity; Fast results; Multicolor multidetection; Single‐cell level assay	Time consuming; Limitations of resolution; Unable to detect equilibrium translocation; More expensive	HER2 amplification ALK rearrangement	[73]
PCR	Amplifies a specific gene or transcript and quantifies the amplified template molecules	High sensitivity; Product availability; Easy and fast operation; Absolute quantitative capability	Easily contaminated; High sample requirements; Data interpretation needs strict controls; Unable to distinguish between low and high transcription cells	BRAF EGFR KRAS PIK3CA PDGFRA FGFR	[74]
NGS	High‐throughput sequencing of millions to billions of DNA fragments in parallel	Multicancer early screening; Precise detection; Safe and fast	Higher costs; Complex data analysis; High sample quality and quantity	EGFR KRAS ALK RET MET NTRK BRAF BRCA	[75]
Sanger sequencing	The gene(s) are amplified by PCR, then any of several direct methods are used to determine the sequence of the entire PCR product	High accuracy; Mature operation; Cost controllable; Long‐read sequencing; Visualization of results	Low throughput; Rely on specific primers; Difficulty in handling homodimers; Not suitable for long genome sequencing	HLA‐typing	[76]

### 3.1 Targeting EGFR Mutations

Over the past decade, the identification of key driver gene mutations has become paramount in enhancing treatment precision and, consequently, improving patient survival rates. Epidermal growth factor receptor (EGFR) sensitizing mutations are among the most critical driver gene mutations in lung cancer [77]. In the Chinese non‐small cell lung cancer (NSCLC) population, the EGFR mutation rate is 28.2%, while in lung adenocarcinoma, this proportion can reach as high as 50.2% [78]. Moreover, EGFR‐targeted therapies have shown effectiveness not just in lung cancer but also in several types of cancer [79]. Clinically significant EGFR‐activating mutations in individuals with lung adenocarcinomas are typically located within its kinase domain, encompassing exons 18, 19, 20, or 21 [80, 81]. These discoveries have paved the way for the development of first‐generation EGFR tyrosine kinase inhibitors (TKIs), including gefitinib and erlotinib [82], followed by second‐ and third‐generation TKIs such as afatinib [83] and osimertinib [84], respectively. The advent of first‐generation EGFR TKIs marked a paradigm shift in the therapeutic management of patients with metastatic NSCLC carrying common sensitizing EGFR mutations [85]. By reversibly binding to the ATP‐binding pocket of the EGFR tyrosine kinase domain, these inhibitors effectively prevent autophosphorylation and the propagation of downstream signaling. The second‐generation TKIs enhance the antitumor activity through irreversibly binding to members of the ERBB family and significantly prolong the survival period in the first‐line treatment [86]. However, patients typically developed drug resistance within a median of 10–14 months, with the emergence of the T790M mutation posing significant clinical challenges. Although third‐generation EGFR–TKIs have been clinically used for patients with EGFR T790M mutations, the outcomes remain unfavorable [87].

Several CDx assays have been proposed to aid in the selection of suitable patients for these medications. In 2004, the Dako EGFR pharmDx Kit (P030044) was the first US FDA‐approved diagnostic assay for detecting EGFR protein expression in colorectal cancer patients to identify candidates for cetuximab therapy [88]. This study employed Dako EGFR pharmDx Kit (P030044) for retesting the samples, with all results confirming EGFR negativity. This strongly demonstrates that cetuximab is indeed effective for colorectal cancer patients identified as EGFR‐negative via IHC [89]. After 9 years, while approving erlotinib as a first‐line treatment for patients with EGFR mutations, the US FDA announced approval of the Cobas EGFR Mutation Test v1 (P120019) as a CDx for erlotinib in NSCLC patients, making it the first CDx approved by the US FDA capable of detecting EGFR exon 19 deletions or exon 21 L858R substitution mutations [90]. Before the US FDA approval, the Cobas EGFR Mutation Test v1 (P120019) had already demonstrated high concordance (>96%) with the laboratory developed test (LDT) used in the EURTAC trial, showing superior analytical sensitivity and specificity compared with Sanger sequencing [91]. In response to the T790M mutation in exon 20 of EGFR, the US FDA approved the Cobas EGFR Mutation Test v2 (P20019/S007 and P150044) as a tissue‐ and plasma‐based CDx to identify patients eligible for osimertinib therapy. Interestingly, in the AURA2 trial (NCT02094261), the authors used the Cobas EGFR Mutation Test v1 to screen patients confirmed as T790M‐positive, achieving an ORR of 62.3% as verified by blinded independent central review [92]. Additionally, the study compared the clinical impact of switching EGFR CDx from Therascreen to Cobas v2. Although overall mutation detection rates were comparable (35.9 vs. 39.3%), Cobas identified slightly more exon 19 deletions, while Therascreen detected more L858R mutations. No significant difference was observed for T790M detection, suggesting limited clinical impact of this transition [93]. In summary, EGFR mutation CDx has achieved a transition from single gene to multigene testing, integrating NGS with liquid biopsy to enable noninvasive, dynamic genotyping. Consequently, the CDx has evolved from being dominated by a single technology to a mature system where multiple high‐precision platforms, including IHC, PCR, and tissue/liquid NGS, coexist and complement each other, collectively providing decision‐making support for personalized treatment.

### 3.2 HER2 Amplification

HER2 is a transmembrane receptor tyrosine kinase protein encoded by the ERBB2 gene, which normally participates in cell growth and differentiation. Currently, HER2 CDx primarily relies on four categories of detection technologies, including IHC for protein expression, FISH, and chromogenic ISH (CISH) for gene amplification, and NGS for gene copy number, mutations, and other structural variations [94]. In 1997, the US FDA approved the INFORM HER‐2/neu assay kit for marketing, but only for prognostic evaluation. The following year, Dako HerceptTest was approved concurrently with trastuzumab and became the first CDx defined by the US FDA [95]. HER2 status assessment is typically performed early using an IHC scoring system (0–3+). For IHC 2+ samples, FISH testing for ERBB2 gene amplification must be performed to confirm positive or negative status [96]. The HERA trial demonstrated that 1 year of adjuvant trastuzumab therapy significantly improves disease‐free survival and overall survival in patients with early‐stage HER2‐positive breast cancer [97]. Despite ongoing debate over HER2‐low scoring, patients with IHC 1+ or 2+/ISH‐negative disease showed PFS benefits.

CISH is a molecular pathology technique used to detect specific DNA sequences or chromosomal abnormalities on formalin‐fixed, paraffin‐embedded tissue sections [98]. In 2008, the SPOT‐LIGHT HER2 CISH kit received US FDA approval, replacing the need for fluorescence microscopy in FISH testing. However, CISH falls short of FISH in terms of sensitivity, standardization, consistency, and clinical validation, limiting its clinical application [99, 100]. Recently, in January 2025, the PATHWAY anti‐HER2/neu (4B5) assay based on CISH and dual‐color CISH was not only employed in clinical trials to precisely distinguish HER2‐low and ‐ultralow populations, but also received formal US FDA approval as the first CDx for identifying patients with HER2‐ultralow breast cancer [101]. Its application directly facilitated the expanded indication of Enhertu, allowing more patients with extremely low HER2 expression to benefit from this targeted therapy [102]. This opens an unprecedented new pathway for HER2‐targeted therapy, empowering clinicians to improve outcomes for patients facing this challenging disease.

HER2‐targeted therapy is also the standard treatment regimen for patients with locally advanced or metastatic gastric cancer (GC) and biliary tract cancer [103]. In 2010, the sole approved CDx devices for GC were IHC for HER2 overexpression and FISH for HER2 amplification [104]. In this study, the authors performed quantitative HER2 protein analysis using the PATHWAY anti‐HER2/neu (4B5) assay in biliary tract cancer patients with ISH‐confirmed HER2 amplification [105]. These findings highlight the importance of combining ISH and IHC testing to accurately identify patients most likely to benefit from HER2‐targeted therapy. Therefore, on November 20, 2024, the US FDA granted approval to the PATHWAY anti‐HER2/neu (4B5) CDx to test biliary tract cancer. Overall, the development of HER2 CDx has advanced from merely determining positivity to now enabling more precise stratification of HER2 expression intensity levels, providing accurate classification for a broader patient population.

### 3.3 PD‐1/PD‐L1 ICIs

Encouragingly, the recent advent of immune checkpoint blockade targeting programmed death‐1 (PD‐1) and PD‐L1 has brought about a transformative shift in the treatment landscape for many cancers [106]. Currently, PD‐L1 assessment using IHC remains the most widely implemented and clinically validated CDx to guide anti‐PD‐1/PD‐1/PD‐L1 therapy, although its predictive accuracy is imperfect and varies across tumor types and assay platforms [107]. However, the clinical utility of PD‐L1 testing varies substantially depending on the specific cancer type and treatment context. Consequently, employing distinct CDx assays tailored to individual therapy agents becomes imperative [108]. Most US FDA‐approved CDx are designed for pretreatment use in NSCLC. For instance, anti‐PD‐1/PD‐L1 antibodies for NSCLC treatment encompass nivolumab (Opdivo) [109], pembrolizumab (Keytruda) [110], atezolizumab (Tecentriq), and cemiplimab (Libtayo) [111]. Ohyanagi et al. conducted a molecular assessment of the Oncomine Dx Target Test CDx System (Oncomine DxTT) using formalin‐fixed paraffin‐embedded lung tissue samples, aiming to assess the test success rate and turnaround time. Their findings indicated that Oncomine DxTT could streamline the testing process for multiple biomarkers in small tissue samples [112]. In another study, Marwitz et al. investigated epigenetic modifications of PD1, PD‐L1, and CTLA4 in NSCLC tissues from 39 patients. They observed significant differences in the CpG‐methylation patterns between tumor tissues and matched controls, particularly noting decreased methylation of CTLA4 and PD1 genes in tumor tissues compared with matched tumor‐free tissues from the same patients [113]. Hersom et al. delved into the clinical performance of PD‐L1 IHC 22C3 pharmDx, PD‐L1 IHC 28‐8 pharmDx, and the Ventana PD‐L1 (SP142) assays, including discussions on the selection of clinical cutoff values [114, 115]. The robustness and reproducibility of the PD‐L1 IHC (SP263) assay were highlighted in a clinical context, meeting all predefined acceptance criteria [116].

As the need for an increasing number of tests becomes imperative in newly diagnosed patients, standardization of assays and a deeper understanding of the biology of candidate markers assume growing significance in both research and clinical practice [117]. In addition to NSCLC, PD‐L1 CDx has received US FDA approval to identify patients eligible for Keytruda or Tecentriq treatment across seven additional tumor indications, using clinically validated combined positive score (CPS) cutoffs. Moutafi et al. have suggested that CPS exhibits greater sensitivity than tumor cell assessment, particularly in GC/gastroesophageal junction. They propose that evaluating the tumor microenvironment should be considered when predicting responses to PD‐1 axis immunotherapy [118]. To ensure precision and accuracy in immunotherapy decisions, the Flagship Biosciences’ Computational Tissue Analysis platform was employed for the Ventana PD‐L1 SP263 and Dako PD‐L1 22C3 assays [119].

PD‐L1 testing is one of the most active CDx areas in immunotherapy. Chang et al. comprehensively summarized US FDA‐approved CDx assays associated with PD‐1 and PD‐L1, examining 70 Biologic Licensing Applications or supplement approvals, of which 32 were granted, with 21% involving CDx devices [120]. Building upon regulatory overview, Dolled‐Filhart et al. provided supporting evidence for the clinical utility of a PD‐L1 IHC assay employing the 22C3 anti‐PD‐L1 murine monoclonal antibody on the Dako platform as a viable CDx for pembrolizumab in NSCLC patients [121]. At the experimental level, studies utilizing the microtube array membrane demonstrated that combination therapy targeting PD‐1/PD‐L1 significantly enhanced T‐cell activity, resulting in up to a 70% reduction in tumor cell viability and an 82% decrease in cytotoxic effects [122]. Complementing these findings, Hansen et al. emphasized that the isolated development of CDx assays for individual PD‐1/PD‐L1 agents, without harmonization, can lead to inflexibility in clinical practice where an assay validated for one agent cannot be applied to another within the same drug class and indication [123]. While CDx testing for ICIs shows reproducibility in tumor cell PD‐L1 expression, it remains inconsistent in immune cells. Even with simplified scoring systems, substantial variability persists between assays and pathologists in assessing immune cell PD‐L1 expression [124]. Finally, Mizutani et al. further validated the rational use of nivolumab, rather than other PD‐1 antibodies, as a capture antibody in ELISA, providing a more reliable and clinically relevant approach for quantifying soluble PD‐1 levels in serum [125].

### 3.4 BRAF and Other Kinase Mutations

BRAF is a key regulator of the mitogen‐activated protein kinase (MAPK)/ERK signaling pathway [126]. Mutations in the BRAF gene occur in about 70% of cutaneous melanomas, most commonly the V600E substitution, which mimics activation loop phosphorylation and disrupts the inactive kinase conformation [127]. Vemurafenib stands as a novel small molecule renowned for its specific inhibitory action against mutant B‐RF proteins. It exhibits remarkable specificity and potency when targeting melanoma and colorectal cell lines harboring the V600E mutation. The development of vemurafenib as a cancer therapy is set to benefit immensely from a CDx test tailored to detect the BRAF V600E mutation, serving as a pivotal biomarker for patient selection [128]. Cheng et al. have detailed the successful codevelopment of a first‐in‐class, selective inhibitor for the oncogenic BRAF kinase, alongside the Cobas 4800 BRAF V600 Mutation Test. In a similar vein, Halait et al. have provided a comprehensive account of the analytical performance characteristics of the Cobas 4800 BRAF V600 Mutation Test, meticulously designed for the detection of V600E (1799T>A) mutation DNA in formalin‐fixed paraffin‐embedded tissue samples [129]. Notably, the Cobas BRAF Mutation Test has demonstrated significantly superior performance characteristics when compared with the ABI BRAF test and bidirectional direct sequencing [130]. Currently, BRAF CDx primarily relies on PCR and NGS technologies. PCR methods are typically used for rapid detection of single common mutations, such as V600E, offering sensitivity and low cost. NGS, meanwhile, can simultaneously detect multiple gene mutations or fusions, supporting personalized treatment for complex tumors. Each technology possesses distinct advantages and plays a vital role in clinical practice.

Kinase‐driven malignancies beyond melanoma also benefit markedly from CDx‐guided therapy. For example, EGF and EGFR activation triggers two major downstream prooncogenic signaling pathways, including the MAPK cascade (RAS–RAF–MEK–ERK) and PI3K–AKT–mTOR pathway, respectively [131]. Bauml et al. clinically validated the use of the specialized Guardant360 CDx to identify NSCLC patients with KRAS p.G12C mutations who are eligible for sotorasib therapy [132]. The MET amplification or exon 14 skipping mutation stands out as sufficiently predictive properties for drugs targeting MET in the context of NSCLC [133]. In a clinical trial (NCT02143466) evaluating the combination of EGFR inhibitor osimertinib with MET inhibitor treatment in patients with first/second‐generation EGFR TKI resistance, T790M‐negative, and MET amplification advanced NSCLC, savolitinib demonstrated significant antitumor activity with ORR of 64.5% and PFS of 9–11 months [134]. This study employed Foundation Medicine CDx T7 panel to perform NGS testing on 39 tumor samples, successfully identifying NSCLC patients harboring METex14 mutations [135]. These patients subsequently received capmatinib treatment and achieved significant clinical responses, with tumor shrinkage ranging from 14 to 83%, validating the efficacy in identifying the beneficiary population. ALK is a receptor tyrosine kinase whose gene rearrangements generate oncogenic fusion proteins driving cancer progression [136]. In a clinical comparison of ALK inhibitors, Kang et al. demonstrated that alectinib yields superior progression‐free survival, duration of response, and overall survival compared with crizotinib in ALK‐positive NSCLC identified using IHC‐based CDx [137]. Complementary prospective validation by Takeuchi confirmed that the expression level of ALK fusion protein does not correlate with therapeutic response, supporting IHC CDx as a reliable and efficient screening modality regardless of quantitative expression [138]. Similarly, other receptor tyrosine kinases (such as RET and ROS1) also require CDx for patient selection. RET gene fusions can be detected via NGS or FISH, thereby guiding the use of selective RET inhibitors like selpercatinib. ROS1 gene rearrangements are typically identified via NGS, providing the basis for targeted therapy with crizotinib or entrecitinib. Furthermore, NTRK1, NTRK2, and NTRK3 gene fusions, widely present across various tumor types, serve as targets for US FDA‐approved TRK inhibitors. Detection methods include IHC for initial screening, followed by validation via NGS or FISH to ensure accurate identification of fusion‐positive patients. Finally, for lipid kinase targets like PIK3CA, CDx based on NGS or allele‐specific PCR can identify activating mutations, informing the use of PI3K inhibitors in cancers such as breast cancer. Collectively, these examples highlight the critical role of CDx in guiding targeted therapies across a wide spectrum of kinase‐driven malignancies.

### 3.5 HRR Genes and PARP Inhibitors

Researchers estimate that approximately 30–50% of ovarian cancers are associated with homologous recombination (HR) deficiency. Pathogenic variants in two critical HR genes, BRCA1 and BRCA2, represent some of the earliest and most well‐established genetic markers of HR deficiency [139]. Consequently, targeted therapies such as poly ADP ribose polymerase (PARP) inhibitors have emerged as highly promising treatments for ovarian cancer, particularly among women with BRCA1/2 mutations or those lacking a functional HR repair pathway. To better understand the safety profiles of these agents, LaFargue et al. conducted a comprehensive evaluation of the toxicities associated with different PARP inhibitors, both when used alone and in innovative combinations with other drugs [140]. In clinical practice, several US FDA‐approved CDx assays are used to guide PARP inhibitor therapy in ovarian cancer. In 2014, the US FDA approved BRACAnalysis CDx, the first LDT CDx approved through the PMA pathway. Unlike many earlier DNA‐based CDx tests, BRACAnalysis CDx detects multiple types of mutations in the BRCA1/2 genes, including single nucleotide variants, small insertions/deletions, and large rearrangements, and classifies them as deleterious or suspected deleterious to guide treatment decisions for Olaparib. In 2016, FoundationFocus CDxBRCA Assay was developed to identify patients likely to respond to rucaparib [141] and BRACAnalysis is unique in several respects, including its comprehensive BRCA gene germline profiling, accessibility to all women with ovarian cancer, and implications for family members [142].

PARP inhibitors CDx is also utilized for pretreatment diagnosis in pancreatic, breast, metastatic castrate resistant prostate cancer, and prostate cancers [143, 144, 145]. In the clinical setting, multigene signature assays such as Oncotype DX, MammaPrint, and Prosigna have been integrated to tailor decisions regarding adjuvant endocrine and chemotherapy treatments. For example, Kumar et al. conducted an evaluation of androgen receptor (AR) expression by IHC in patients with advanced triple‐negative breast cancer (TNBC) before initiating treatment with the AR inhibitor enzalutamide [146]. Olaparib has received approval for the treatment of patients with germline BRCA1/2 mutations who have HER2‐negative advanced or recurrent breast cancer. The BRACAnalysis diagnostic system was introduced as CDx, significantly advancing breast cancer treatment based on genetic diagnosis [147].

Testing high‐grade serous ovarian carcinoma tissues for BRCA mutations offers the potential to identify individuals harboring either germline or somatic mutations, thereby enabling their treatment with PARP inhibitors. Kwon et al. conducted a comprehensive cost‐effectiveness analysis to compare the utility of universal germline testing against tumor testing as a CDx for PARP inhibitor therapy [148]. Wehnelt et al. conducted a rigorous validation of the analytical performance of germline testing in the context of ovarian cancer therapies. They utilized hybridization‐based target enrichment (Agilent) incorporating a panel of 10,742 probes to assess its reliability and accuracy. Sotgia et al. made significant strides in the field by identifying and utilizing mitochondrial biomarkers as novel CDx tools. These biomarkers have the potential to predict clinical outcomes and enhance therapeutic responses in ovarian cancer patients [149]. Furthermore, research has indicated that the prevalence of BRCA gene mutations is higher in tumor cells compared with germline cells. Copeland et al. demonstrated that a tumor‐based testing approach has the potential to identify a greater number of individuals who may benefit from treatment compared with germline testing [150]. In contrast, microsatellite instability‐high was found to be less commonly detected as a CDx for pembrolizumab treatment in unselected gynecologic patients [151]. It is imperative to emphasize that genetic counseling should always accompany treatment selection. Last, this paper provides an overview and analysis of US FDA‐approved CDx assays for three specific targeted treatments for ovarian cancer: vintafolide and etarfolatide [152], lynparza (olaparib) [153], and rucaparib [154]. These assays represented crucial advancements in tailoring treatments to individual patient profiles.

### 3.6 Emerging Applications in Nononcology Diseases

In addition to its applications in oncology, patient‐specific biomarkers hold significant promise for enhancing the diagnosis and treatment of various other diseases. For example, CDx also plays a crucial role in hereditary red blood cell disorders, such as nontransfusion‐dependent thalassemia. Exjade is an iron chelator indicated for the treatment of chronic iron overload in patients with nontransfusion‐dependent thalassemia [155]. In 2013, the US FDA approved FerriScan as CDx for Exjade, with its core significance lying in providing a precise, noninvasive, and quantitative method for assessing hepatic iron load to guide treatment decisions for iron overload patients [60]. Simultaneously, it became the first CDx based on in vivo imaging, pioneering the use of CDx in noncancer fields. Furthermore, across multiple hematologic disorders (including acute and chronic myeloid leukemias, myelodysplastic syndromes, and systemic mastocytosis), the US FDA‐approved CDx assays provide critical decision‐making support for precision medicine through molecular subtyping, mutation detection, and risk stratification. In 2015, the KIT D816V Assay is a droplet digital PCR and sanger sequence‐based test designed to detect the KIT D816V mutation in bone marrow to assess whether patients with aggressive systemic mastocytosis may benefit from imatinib therapy [156]. Acute myeloid leukemia (AML) constitutes nearly half of all AML cases and exhibits substantial clinical heterogeneity. In the case of AML patients, three CDx testing assays are available for detecting IDH1, IDH2, and FLT3 mutations [157]. The results of these tests inform treatment decisions made by physicians. Interestingly, in 2022, the US FDA approved the first CDx for obesity based on NGS. The POMC/PCSK1/LEPR CDx Panel is a CDx test for blood or saliva samples designed to identify patients with severe early‐onset obesity who carry pathogenic, likely pathogenic, or variants of uncertain significance in the POMC, PCSK1, or LEPR genes [158]. These patients may benefit from treatment with setmelanotide.

Beyond the CDx already approved by the US FDA for specific diseases, researchers both domestically and internationally are actively exploring additional disease‐related biomarkers and are committed to developing new CDx to expand their applications in precision medicine. For instance, common targets shared by COVID‐19 and chloroquine, such as GSTA2, TNF, TLR9, GST, HMGB1, and GSTM1, have been identified [159]. Emerging literature highlights the potential utility of various biomarkers, including sTREM‐1, IL‐27, suPAR, neutrophil CD64, presepsin, cell‐free DNA (cfDNA), and miRNAs, as novel CDx options for sepsis diagnosis [160]. In the context of RVO, intraocular biomolecules such as VEGF, IL‐6, IL‐8, MCP‐1, sICAM‐1, IL‐12, IL‐13, sVEGFR‐1, sVEGFR‐2, and PDGF‐AA have been reported [161]. Furthermore, CDx also plays a crucial role in various therapeutic domains, including neuroscience, immunology, and rare diseases such as human immunodeficiency virus (HIV) [162], Alzheimer's disease [163], mycobacterium tuberculosis [164], and Lyme disease [165]. Notably, the recent emphasis on the high‐throughput analysis of exosomal molecular contents has brought this topic to the forefront of discussions. Exosomes have emerged as potential candidates for CDx development in the realm of neurodegenerative disorders. ESAT‐6, a pivotal vaccine antigen with unique properties, is incorporated into several vaccine candidates under development for tuberculosis [166]. Urinary thioredoxin serves as a biomarker for diagnosing tubular redox dysregulation and as a CDx for identifying responders to redox‐modulating therapeutics in kidney disease [167]. Serum periostin was identified as a CDx for targeted therapy against refractory Th2/eosinophilic inflammation and for asthma management [168].

## 4 Methodological Platforms for CDx

**FIGURE 2:**
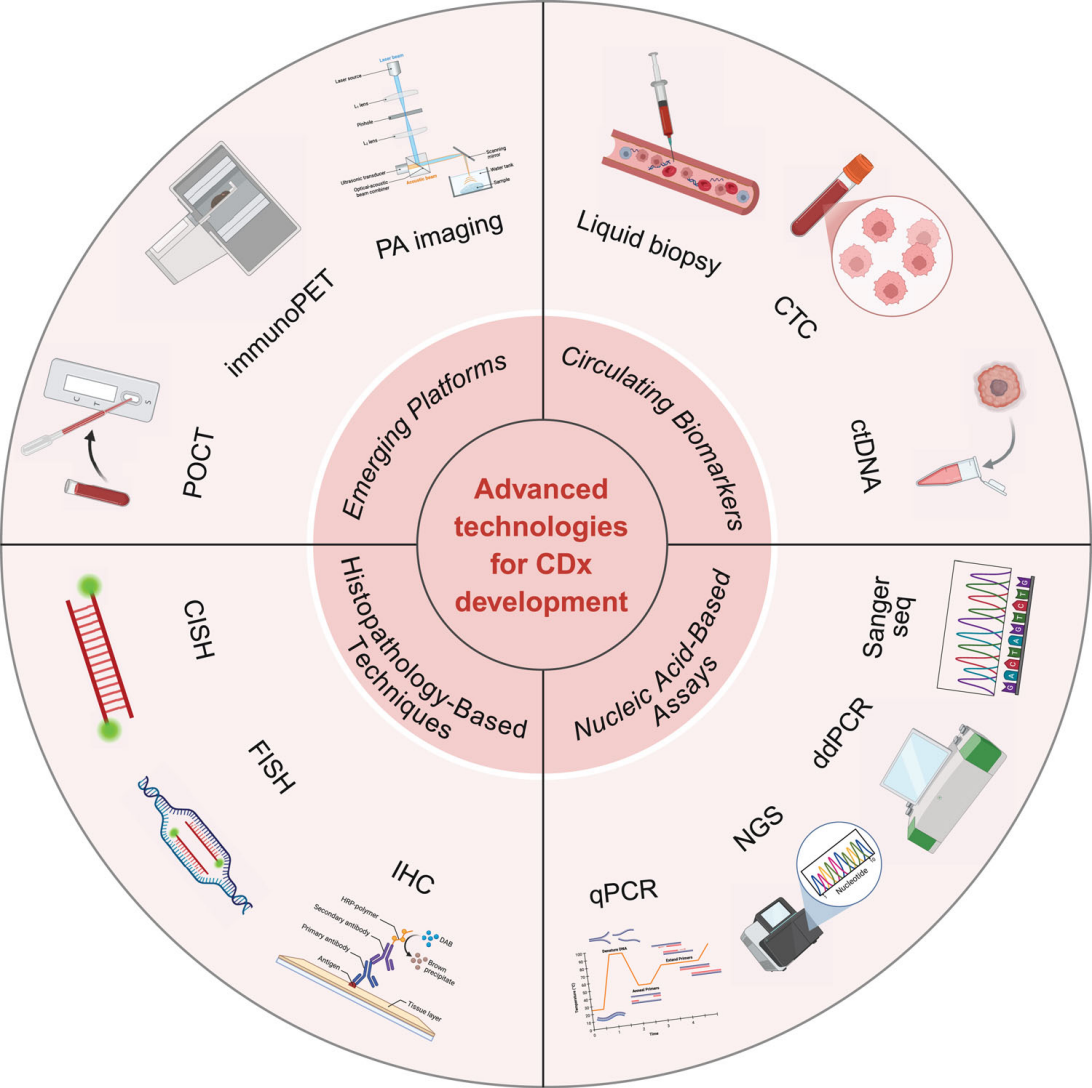
A suite of advanced methods and technologies are used to guide treatment, monitor progress, and predict efficacy. These include traditional and emerging histopathology techniques, highly sensitive nucleic acid assays, emerging minimally invasive circulating biomarker assays, and cutting‐edge imaging and real‐time detection platforms, all of which work together to build a technological support system for precision diagnosis and treatment. Created with BioRender.

The advancement of CDx relies on diverse analytical and detection platforms that provide complementary biological insights across molecular, cellular, and functional levels. This section will provide a comprehensive review of the current mainstream and emerging CDx technology platforms, including nucleic acid testing, histopathology‐based testing, circulating biomarker analysis, imaging, and point‐of‐care technologies (Figure 2).

### 4.1 Nucleic Acid‐Based Assays: PCR and NGS

PCR uses a DNA polymerase to replicate target DNA sequences in vitro, allowing exponential amplification of specific genetic regions [169]. PCR technology is characterized by high sensitivity, specificity, and operational convenience, making it widely adopted in clinical diagnostics, molecular research, and other applied fields [170]. In the context of CDx, PCR accounts for 32.76% and allows for the rapid detection of known genetic variants, often achieving single‐digit mutant allele sensitivity. Typical amplification reactions can be completed within 2–4 h, requiring only crude DNA extraction, which makes it suitable for routine clinical laboratories. Today, PCR technology has undergone three generations of technological evolution, including qualitative PCR, real‐time quantitative PCR (qPCR), and digital PCR (dPCR), with continuously improving accuracy and sensitivity [171]. qPCR, as one of the most mature and standardized molecular detection technologies currently available, has gained widespread adoption in clinical settings due to its high sensitivity, excellent reproducibility, and controllable costs. It remains the mainstream technology platform for CDx such as the Therascreen EGFR RGQ PCR Kit and Cobas KRAS Mutation Test and routine clinical testing at this stage. Although dPCR has evolved into a highly sensitive and quantitative molecular detection technology, as for now, there are no gene testing products explicitly based on the dPCR platform that have been approved by US FDA‐listed CDx in the public database. dPCR has been applied in multiple clinical trials, including studies on ovarian cancer [172], CML [173], and glioma [174]. The technology demonstrates high precision and sensitivity in detecting target genetic alterations, offering valuable information for treatment management. However, the high cost of dPCR currently limits its clinical use, though future cost reductions and technical improvements may enable broader adoption.

While PCR excels in detecting predefined mutations, its scope is limited in the context of tumor heterogeneity, motivating the transition toward broader NGS‐based platforms [175]. NGS, often referred to as high‐throughput sequencing, enables simultaneous analysis of millions of DNA/RNA fragments and accounts for 22.41% of US FDA‐approved CDx assays. This remarkable capacity empowers researchers to explore the genetic landscape of diseases in depth, revealing intricate variations and alterations that were previously inaccessible. A standard NGS workflow consists of four key stages: sample preparation, amplification, sequencing, and data analysis [176]. CDx demands the precise identification of genetic mutations, variations, or expression patterns that influence a patient's response to a specific therapy. NGS excels in this context as it offers a comprehensive view of the entire genome or transcriptome, facilitating the detection of not only known biomarkers but also unexpected genetic anomalies that may impact treatment decisions [177]. By analyzing the specific genetic complexity of each patient, CDx tests driven by NGS enable healthcare providers to develop personalized treatment strategies with unparalleled precision. This approach can minimize the risk of adverse reactions to the greatest extent and optimize the therapeutic effect, thus ushering in a new era of patient‐centered care [178]. Heterogeneous NGS technologies play a crucial role in the development of CDx and personalized medicine. For instance, Kato et al. devised a novel NGS‐based compact panel with impressive mutation detection limits: 0.14% for EGFR exon 19 deletion, 0.20% for L858R, 0.48% for T790M, 0.24% for BRAF V600E, and 0.20% for KRAS G12C. Their analysis results indicate that this panel can effectively accommodate various biopsy samples routinely obtained in clinical practice, and no strict pathological supervision is required [179].

NGS still faces technical bottlenecks, high costs, and limited hospital adoption and insurance coverage. Overall, PCR meets routine testing needs while NGS is suited for high‐throughput analysis. Their application scenarios have low overlap, forming a complementary relationship. In the short to medium term, PCR remains the mainstream molecular diagnostics (MDx) platform, while NGS represents the future development trend.

### 4.2 Histopathology‐Based Techniques: IHC and ISH

IHC stands as a specialized laboratory technique that facilitates the visualization and quantification of specific proteins or biomarkers within tissue samples. By labeling specific antibodies, it can detect the presence of specific protein antigen in tissue sections and present the test results through color reactions [180]. The process of IHC testing encompasses five essential steps, including sample preparation, antigen retrieval, primary antibody incubation, secondary antibody binding, and visualization [181]. IHC enables the stratification of patients according to their suitability for specific therapies, forming a fundamental component of CDx assays. Among US FDA‐approved CDx tests, those based on IHC technology account for approximately 19.68%, primarily detecting PD‐L1 or HER2 expression in cancer treatment. For example, the PD‐L1 IHC 22C3 pharmDx assay has been demonstrated to be a sensitive, accurate, and reliable CDx that facilitates the safe and effective use of pembrolizumab in patients with NSCLC and squamous cell carcinoma [182, 183]. Comparative analysis and efficacy evaluation of PD‐L1 detection methods, along with a comprehensive review of PD‐1/PD‐L1 immune checkpoint inhibitors, further substantiate the clinical utility of immunohistochemical testing. Twomey et al. provided a comprehensive summary of US FDA‐approved ICIs, with a specific focus on monoclonal antibodies targeting PD‐1 or PD‐L1 [184]. Torlakovic et al. conducted an assessment of the performance of PD‐L1 IHC assays, evaluating their diagnostic accuracy at specific cut‐off points, as established for specific immunotherapies, in accordance with the clinical efficacy demonstrated in pivotal clinical trials [185].

Despite its strengths, IHC faces technical and interpretative challenges that affect reproducibility and interlaboratory concordance. The PD‐L1 antibody 22C3 represents the approved CDx IHC test for the treatment of various cancer types with pembrolizumab and cemiplimab. Both the 22C3 and 28‐8 antibodies target the extracellular domain of PD‐L1, which is known to contain N‐glycosylation sites. Fernandez et al. conducted tests on samples over time and assessed the impact of time and deglycosylation on PD‐L1 signal by comparing an antibody utilizing an extracellular domain antigen to one employing an intracellular domain antigen. The results indicated that the glycan component of the 22C3 epitope is not stable over time [186]. The broader implementation of such IHC assays holds the potential to promote a more rational and cost‐effective pharmacotherapy, benefiting both individual patients and the healthcare system at large [187]. Furthermore, to interpret the results of a complex CDx IHC assay, Dennis et al. established a digital image‐based training approach, conducted under highly controlled conditions [188]. This approach can effectively substitute for traditional live microscope training and yield skills equivalent to those achieved with face‐to‐face training using conventional microscopy.

ISH is a molecular detection technique that visualizes the distribution of specific DNA or RNA directly within their spatial context by hybridizing nucleic acid probes to complementary sequences within tissues or cells [189]. Traditional FISH is the most used form of ISH, employing fluorescent probes to detect genomic alterations such as gene amplification, deletion, and rearrangement, including HER2 amplification and ALK or ROS1 rearrangements. It serves as a crucial method for CDx in multiple tumors [190]. Currently, CDx based on FISH technology accounts for 6.91%, representing a relatively low overall proportion. This phenomenon reflects to some extent that FISH primarily focuses on specific types of genomic alterations such as gene amplification, deletion, or rearrangement, resulting in a relatively limited scope of application. Although FISH offers advantages including high specificity, strong visualization, and relatively objective results, its complex operation, high cost, and low detection throughput make it difficult to meet the current trend toward multigene, multitarget precision medicine [190]. Therefore, in the CDx field, FISH is more commonly used as a confirmatory detection technique for key sites rather than as a mainstream method for large‐scale screening. With the rise of high‐throughput and high‐sensitivity technologies such as NGS and digital PCR, FISH continues to hold an important position in CDx but its relative proportion has gradually declined. It is increasingly employed for precise confirmation of specific genetic events.

### 4.3 Circulating Biomarkers: Circulating Tumor DNA and Circulating Tumor Cells

Liquid biopsy refers to a technology that utilizes human bodily fluids as the source of specimens to detect and obtain tumor‐related information [191]. Liquid biopsy testing holds the potential to furnish critical insights into various tumor biological characteristics, encompassing tumor progression, metastasis, tumor heterogeneity, genomic mutation profiles, and clonal evolution [192]. Notably, in 2016, the US FDA granted approval for the first circulating tumor DNA (ctDNA) liquid biopsy test designed to identify EGFR gene mutations in patients with NSCLC. This test serves as a CDx for EGFR–TKIs. The next phase of integrating liquid biopsy into routine clinical practice revolves around monitoring ctDNA postsurgical intervention, offering early prognostic insights and enabling the detection of disease relapse well before conventional imaging diagnosis [193]. In addition to ctDNA, circulating tumor cells (CTCs), cfDNA, proteins, cytokines detected in plasma, and circulating T‐lymphocytes are being explored as potential sources for the development of new immune‐oncology biomarkers [194]. Up until 2022, the US FDA has granted approval for several single‐gene assays and more recently, multigene assays for the detection of genetic alterations in plasma cfDNA. These assays are tailored to specific molecularly targeted therapies for cancer, marking a significant turning point in the widespread adoption of liquid biopsy, particularly among patients with advanced‐stage cancer [195].

CTCs and CTC clusters serve a critical function in the fundamental mechanism of tumor cells detaching from the primary site and disseminating to distant locations [196]. CTCs hold promise as a real‐time surrogate source of cancer cells, offering opportunities for the evaluation of predictive biomarkers that can inform treatment decisions [197]. The analysis of CTCs can yield valuable insights into genetic mutations, gene expression patterns, and protein markers within a patient's tumor, facilitating more precise treatment selection. Furthermore, changes in the number or characteristics of CTCs in the blood can serve as indicators of treatment efficacy or the emergence of drug resistance [198]. This continuous real‐time monitoring is a critical component of personalized medicine, which is facilitated by CDx [199]. Recent studies have underscored the predictive significance of monitoring CTC levels for both progression‐free and overall survival in diverse cancer types, including breast cancer [200], lung cancer [201], prostate cancer [202], and colorectal cancer [203]. However, the challenge of obtaining sufficient CTCs has prompted innovative solutions. Patil et al. have introduced a novel method to harvest an adequate quantity of CTCs for PD‐L1 profiling using immunocytochemistry. This approach enables the quantitative determination of PD‐L1 expression, a parameter with clinical significance in predicting favorable responses to checkpoint inhibitor therapies [204]. To enhance capture efficiency, a reported fluorescent virus‐guided system for viable CTC capture has the capability to image both epithelial and mesenchymal tumor cells exhibiting telomerase activity as GFP‐positive cells [205]. Temporal fluctuations in CTC counts among patients with epithelial tumors have demonstrated a correlation with disease progression, as evaluated through conventional radiographic methods [206]. Efforts to collect CTCs using a microfluidic platform, designed for efficient and selective separation of viable CTCs from peripheral whole blood samples, offer a promising tool for precise identification and measurement of treatment responses [207, 208]. While CTCs enumeration serves as a marker for prognosis and survival, molecular characterization of CTCs may provide a more accurate means of monitoring treatment response, especially considering tumor heterogeneity across primary and metastatic sites [196]. ctDNA has also shown promise as CDx biomarkers for cancer screening, prognostication, and patient surveillance in various cancer types, including ovarian cancer [209] and melanoma [210].

### 4.4 Emerging Platforms: Imaging‐Based CDx and Point‐of‐Care Technologies

In vitro CDx assays are limited in their ability to precisely locate tumors and small metastases. Furthermore, they do not facilitate real‐time treatment monitoring. However, several medical imaging techniques provide dynamic assessments of tumor heterogeneity and treatment responses, thus complementing imaging‐based CDx tests [60, 211]. Consequently, innovative molecular imaging‐based CDx approaches have emerged to enable real‐time tumor diagnosis and treatment response monitoring, thereby advancing precision medicine. Recent advancements in molecular imaging have afforded the capability to assess tumor heterogeneity and dynamically monitor treatment responses. Table 3 summarizes studies related to biomarker‐based CDx imaging strategies in recent years. These techniques encompass nuclear medicine methods [212, 213], ultrasound imaging [214, 215], MRI [216], computed tomography (CT) imaging [217], and optical imaging [218]. They serve the dual purpose of predicting therapeutic efficacy and stratifying patients. In 2013, FerriScan was first approved by the US FDA for the assessment and treatment monitoring of iron overload disorders. This MRI‐based quantitative analysis technology enables noninvasive measurement of hepatic iron concentration [219]. In a study of children with sickle cell anemia experiencing iron overload due to long‐term blood transfusions, FerriScan demonstrated good reliability and consistency in assessing body iron load [220].

**TABLE 3:** Studies related to biomarker‐based CDx imaging strategies.

Biomarker	Disease model	Drug	Detection method	Mechanism of action	References
APE1	Xenografted model	Radiotherapy; APX3330	MRI	APE1 activity is used as a marker for dynamic monitoring of tumor response to radiotherapy by means of highly sensitive imaging probes to assess early signals of efficacy and dose response.	[221]
gpNMB	TNBC	^89^ZrDFO–CR011	PET	A monoclonal antibody targeting gpNMB (CR011) labeled with ^8^ ^9^Zr via DFO chelation enables PET imaging of gpNMB‐expressing tumors, allowing noninvasive in vivo visualization and quantitative assessment of gpNMB to support targeted therapy.	[222]
CDH17	Xenografted model	^68^Ga–NOTA–CDH1; Al^18^F–RESCA–CDH1	ImmunoPET	Immuno‐PET imaging for noninvasive detection of tumor CDH17 expression has demonstrated different imaging sensitivities and targeting capabilities in different tumor models.	[223]
Trop2	Solid tumors	^68^Ga–NOTA‐T4	ImmunoPET	Radioactive PET tracers reflect the distribution of Trop‐2 expression in tumor tissues on PET images through high‐affinity recognition of Trop‐2.	[224]
CD38	Multiple myeloma	[^68^Ga]Ga–TOHP–CD3813	ImmunoPET	TOHP–CD3813 specifically binds to CD38 on the surface of tumor cells and helps to accurately image CD38 expression through radiolabeling in clinical or experimental settings, thus supporting the diagnosis and efficacy assessment of multiple myeloma.	[225]
PDL1	Lung adenocarcinoma	^99^mTc‐NM‐01	SPECT	NM‐01 binds to various PD‐L1‐positive cancer cell lines and interacts only with PD‐L1 expressed on the cell surface.	[226]
P‐tau217	Alzheimer's disease	—	Electrochemiluminescence	Accurately predicts amyloid PET positivity in cognitively unimpaired individuals	[227]
GDF‐15	Epithelioid hemangioendothelioma	Sirolimus	CT	GDF‐15 predicts epithelioid hemangioendothelioma aggressiveness.	[228]
CD24; HER2; MUC16	Endometrial cancer	[^89^Zr]Zr–DFO–ATG‐031; [^89^Zr]Zr–DFO–trastuzumab	ImmunoPET	Noninvasive staging and monitoring of the disease and as companion imaging agents for HER2‐ and CD24‐targeted therapeutics	[229]
LAG‐3	Xenograft models	[^68^Ga]Ga–CC09‐1	ImmunoPET	A promising PET tracer for quantifying the LAG‐3 expression in tumor microenvironment	[230]

Molecular imaging enables the characterization, visualization, and quantification of biological processes at the cellular and subcellular levels within living individuals [231]. Tully et al. have highlighted the development of multiple molecular imaging agents, typically small molecules, for CDx purposes. Examples include ^18^F‐FDG, ^123^I‐MIBG, ^18^F‐FES, ^18^F‐FDHT, and [^18^F]F‐IRS [232]. Additionally, multiplex immunofluorescence and multispectral imaging have underscored the significance of spatial relationships among specific biomarkers in predicting a patient's response to ICIs [233]. The integration of nuclear imaging with established radiopharmaceuticals has introduced new paradigms for evaluating cancer staging, disease status, and treatment response [234]. Efforts in Asia have also advanced nuclear theragnostic, as exemplified by Mishiro, who developed imaging probes for detecting EGFR mutations relevant to osimertinib‐based CDx applications [235, 236]. Photoacoustic (PA) imaging visualizes tissue structure and function by detecting ultrasonic waves generated from transient thermoelastic expansion after biological molecules absorb laser energy at specific wavelengths [237]. Lucero et al. developed a PA imaging‐based CDx for detecting elevated glutathione in a lung cancer model, alongside a corresponding prodrug, PARx, which effectively inhibited tumor growth without off‐target toxicity [238]. In ultrasound molecular imaging, ligand‐functionalized microbubbles have been employed to visualize endothelial targets such as netrin‐1, identified as a potential CDx biomarker in metastatic breast cancer [239].

Immune positron emission tomography (immunoPET) combines PET imaging with the molecular specificity of antibodies or antibody fragments, enabling noninvasive, real‐time quantification of target molecules in vivo [240]. It facilitates patient stratification and drug development, particularly for antibody–drug conjugates, by providing insights into whole‐body biodistribution, pharmacokinetics, and tumor targeting to predict efficacy and toxicity [241, 242]. Specific tracers, [^89^Zr] DFO‐CR011 targeting gpNMB for cancer and ^64^Cu–DOTA‐B‐Fab for CA6 imaging [222, 243]. In a pioneering human trial, ^18^F‐ASIS PET imaging affirmed safe administration and effective tumor imaging [244]. Additionally, magnetic liposome imaging has been validated as a tool to predict tumor responses to nanomedicine therapy without altering biodistribution or immune interactions [245]. The emergence of theranostics, utilizing radiolabeled imaging and therapeutic agents, has solidified its status as a well‐established treatment strategy. This development further reinforces the growth of CDx and personalized medicine [246]. Regarding molecular imaging associated with pharmacological or other treatment categories, there exists a critical imperative for defining and implementing innovative business models that align with the multifaceted needs of the diverse stakeholders within this evolving landscape [247].

POCT involves medical testing or diagnostic processes conducted at locations other than centralized laboratories, typically performed by trained individuals or through self‐testing [248]. POCT is distinguished by its immediacy and convenience, providing rapid results that enable immediate clinical decision‐making [249]. Recently, diagnostic companies have shifted their focus toward two rapidly growing segments of the market: MDx and POCT [250]. Haga offered an overview of implementation research on POCT, covering the benefits, available platforms, and barriers and facilitators related to the development and integration of POCT into clinical settings [251]. Velayudhan et al. discussed various types of POCTs currently utilized for companion and food animal disease diagnostics, along with tests in the developmental pipeline and their respective advantages and disadvantages [252]. A critical challenge in the development of POCT devices is ensuring their effective and sufficient power supply. Choi provided a comprehensive review of techniques for powering POCT devices, addressing their use in both developed and developing countries, and delving into detailed discussions of next‐generation POCT devices and their strategies for power sources [253]. Considering the special requirements of HIV diagnostics in sub‐Saharan Africa, Aleku et al. examined emerging HIV diagnostic platforms, the HIV POCT product pipeline, identified gaps, highlighted perceived challenges in POCT implementation, and offered general recommendations to enhance the quality of care [254].

**FIGURE 3:**
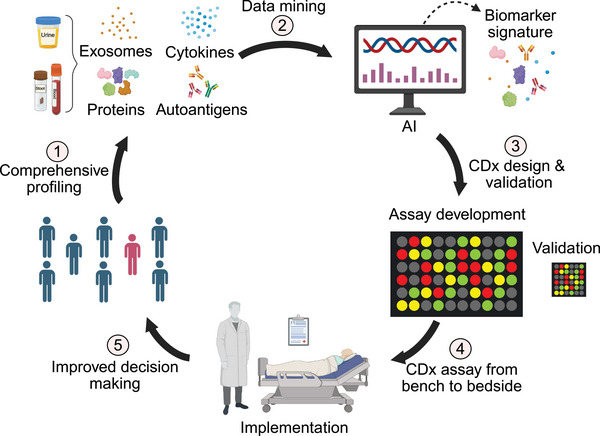
The multistage validation methodology is employed to create and evaluate innovative CDx assays for POCT. The figure demonstrates that POCT is based on the AI platform to mine patients’ multiomics data for clinically significant biomarkers and synchronize the development of CDx with validation to achieve the translation of related technologies from laboratory research to clinical application. Created with BioRender.

POCT‐enabled CDx offer multiple applications across various diseases. Mivacurium chloride, regarded as a crucial adjunct in anesthesia and emergency medicine, is the focus of a point‐of‐care potentiometric sensor designed for accurately and rapidly tracking its enzymatic degradation kinetics [255]. The importance of translating research protocols into multiplexed POCT devices for use in near‐patient settings is emphasized, enabling a more personalized approach to the diagnosis, surveillance, and treatment management of prostate cancer patients [256]. Addressing an unmet clinical need, the development and US FDA approval of noninvasive, rapid (<60 min), in vivo phenotype diagnostic breath tests to assess polymorphic CYP2D6 and CYP2C19 enzyme activity by measuring exhaled ^13^CO_2_ as a biomarker in breath will effectively cater to the demands of individualized psychiatric drug therapy [257]. A nanochannel‐based electrochemical biosensor has been successfully demonstrated for the rapid and multiplexed detection of a panel of three biomarkers associated with sepsis detection: procalcitonin, lipoteichoic acid, and lipopolysaccharide from whole blood [258]. Guan et al. introduced a “contact lens‐on‐a‐chip” CDx testing approach for the personalized selection of compatible contact lens products [259]. Personalization is often achieved through the pairing of a therapeutic strategy with a CDx test that provides individualized information at the point of care (Figure 3).

## 5 Challenges and Considerations in CDx Development and Implementation

The development of CDx is met with substantial scientific, economic, and societal barriers [260]. CDx technology has revolutionized personalized medicine, yet it continues to face multiple challenges in its development and application. These challenges range from the technical validation of detection methods and the biological complexity of tumor evolution to practical obstacles such as regulatory approval, cost, and accessibility. Moreover, the overlapping development of drugs and diagnostic technologies pose unique regulatory and methodological complexities. The following sections will analyze these challenges across four dimensions.

### 5.1 Technical Challenges: Assay Validation, Standardization, and Tissue Versus Liquid Biopsy

Despite significant technological advances, several technical and regulatory challenges continue to constrain the translation of CDx into routine clinical practice. These challenges primarily involve assay validation, cross‐platform standardization, and the complementary use of tissue and liquid biopsy in clinical practice. Assay validation plays a critical role in ensuring the analytical and clinical robustness, encompassing parameters such as bias, precision, repeatability, limits of detection (LoD), linearity, interference, and stability [261, 262]. However, sample heterogeneity significantly distorts validation results. For example, heterogeneity in tumor cell proportion, necrosis, and inflammatory infiltration may compromise test accuracy, resulting in false‐positive or false‐negative outcomes. An analysis examining the relationship between the quality of small biopsy samples for NSCLC and the detection rate of EGFR gene mutations revealed that when the tumor cell proportion was under 30% of the total tissue, the positive detection rate decreased from 47.6 to 29.4% [263]. This finding illustrates how insufficient tumor content can lead to inaccurate molecular testing results, a challenge further exemplified by specific cases reported. Turner et al. reported two cases in which the US FDA‐approved Cobas EGFR Mutation Test failed to detect low‐frequency T790M mutations, or misclassified rare L747S variants as exon 19 deletions, thereby causing patients to be wrongly excluded from the recruitment of clinical trials. In contrast, clinical laboratories confirmed these mutations using rigorously validated in‐house protocols and NGS. This study highlights that although standardized assays offer operational simplicity, they remain limited in addressing tumor heterogeneity, detecting rare variants, and ensuring accurate interpretation [264]. Therefore, clinical practice should implement robust verification systems that employ multiple technical platforms to resolve critical discrepancies and guarantee precise molecular profiling and appropriate targeted therapy for patients.

Moreover, there exists a discrepancy between the lower LoD in clinical and laboratory settings [265]. Under optimized laboratory conditions, NGS‐based ctDNA detection can achieve LoD of 0.1–0.25% for variant allele frequency, yet in real‐world clinical samples, the effective LoD typically increases to approximately 0.5–1% or higher, particularly in patients with low tumor burden or early‐stage cancers [266, 267]. This reduction in detection sensitivity is primarily associated with factors such as variations in sample pretreatment, cfDNA background interference, and sample volume limitations, resulting in a significantly higher effective detection limit in clinical settings compared with laboratory validation [268]. To ensure standardization and comparability among different platforms, Li et al. proposed statistical methods for evaluating the clinical performance of follow‐on CDx through agreement assessments from external consistency studies [269]. They also discuss diverse study designs for concordance studies and proper techniques for estimating agreement between comparator and follow‐on devices.

From a methodological perspective, tissue biopsy offers stable quality and comprehensive molecular characterization, but it also has limitations, such as invasiveness, sample bias, and inapplicability for long‐term monitoring [270]. In cases where the lesion is inaccessible or has undergone metastasis, sample collection may encounter technical difficulties, and the spatial heterogeneity may reduce the representativeness of the obtained tissue [271]. In contrast, liquid biopsy employs ctDNA, CTCs, and extracellular vesicles in plasma to noninvasively reflect the molecular landscape of tumors [272]. The application of liquid biopsy eliminates the necessity for surgical intervention or invasive procedures, enabling repeated collection of tumor samples. This facilitates the establishment of gene expression profiles, identification of targetable mutations, rapid assessment of treatment efficacy, monitoring for the emergence of drug resistance, and adjustment of treatment regimens based on tumor progression [273]. However, sensitivity and specificity are influenced by factors such as biomarker concentration and preprocessing variability [274]. Taken together, rather than serving as a replacement for tissue biopsy, liquid biopsy provides complementary molecular information, where tissue establishes the baseline tumor biology and liquid biopsy enables longitudinal molecular surveillance to guide ongoing clinical decision‐making.

### 5.2 Clinical and Biological Challenges: Tumor Heterogeneity, Biomarker Resistance Dynamics

Although substantial progress has been made in MDx and targeted therapy, the clinical deployment of CDx continues to be constrained by biological complexity, particularly tumor heterogeneity and dynamic biomarker evolution. Among these factors, tumor heterogeneity represents a fundamental barrier to accurate patient stratification and durable treatment response. Tumor heterogeneity refers to the substantial biological diversity that arises among distinct cell populations during tumorigenesis and tumor progression, driven by genetic mutations, epigenetic alterations, and variations in the tumor microenvironment [275]. It is reflected in differences in growth rates, invasiveness, drug sensitivity, and other aspects of cell behavior. Tumor heterogeneity forms the basis for tumor evolution and the development of drug resistance. Based on temporal and spatial characteristics, it can be categorized into temporal heterogeneity and spatial heterogeneity [276]. The former reflects the dynamic changes in the tumor gene lineage of selection pressure at different stages; the latter demonstrates the inconsistency in genes and phenotypes between different parts of the tumor or its metastatic sites [277]. The coexistence of tumor cells and the interplay of signals among them, combined with the presence of subpopulations like stem cells, further exacerbates the complexity within the tumor [278]. Meanwhile, the uneven oxygen supply in the tumor microenvironment, the differences in vascular distribution, and inconsistency of immune infiltration have all contributed to the formation of tumor heterogeneity [279]. For example, hypoxia can induce metabolic reprogramming and immune evasion by activating the SIAH2–NRF1 pathway, while immune cell invasion patterns are closely associated with tumor purity, mutation burden, and neoantigen diversity [280, 281].

The drug resistance of biomarker makes cancer detection techniques more complicated [69]. On one hand, within the therapeutic environment, tumor cells can rapidly acquire resistant phenotypes through multiple pathways. For example, secondary T790M is the primary resistance mutation in NSCLC patients, accounting for approximately 50% of all EGFR–TKI resistance cases [282]. This directly leads to the failure of first‐ and second‐generation EGFR inhibitors. Even with third‐generation inhibitors, new mutations inevitably emerge, resulting in drug resistance. On the other hand, when the major signaling pathway is inhibited, tumor cells can activate alternative signaling pathways to maintain their proliferation, survival, and invasive capabilities, thereby reducing drug efficacy. Taking the EGFR pathway as an example, tumor cells can restore tumor growth through MET amplification, HER2 amplification, FGFR pathway abnormalities, and abnormal activation of IGF1R and AXL [283]. With the advancement of high‐throughput sequencing and single‐cell sequencing technologies, researchers are now able to analyze the evolution processes of tumors and the mechanisms that lead to heterogeneity formation in different spatial and temporal dimensions. This provides a crucial molecular foundation and theoretical support for the design and dynamic monitoring of CDx [284]. Meanwhile, in response to the issue of treatment resistance and biomarker resistance, researchers explored new biomarkers and drugs. Table 4 summarizes the clinical trial situations related to CDx over the past 2 years.

**TABLE 4:** Clinical trials related to CDx over the past 2 years.

NCT No.	Biomarker	Source	Drug	Disease	Phase/status
NCT06680830	LRRK2	Blood; saliva	NEU‐411; placebo	Parkinsons disease	II/recruiting
NCT01227889	BRAF	Tumor tissue	GSK2118436; dacarbazine	Melanoma	III/completed
NCT03816163	Claudin 18.2	Tumor tissue	Zolbetuximab	Pancreatic ductal adenocarcinoma	II/active, not recruiting
NCT05009836	EGFRm+, MET+	Tumor tissue	Savolitinib; placebo	NSCLC	III/active, not recruiting
NCT05622890	Folate receptor alpha	Tumor tissue	Mirvetuximab soravtansine	Ovarian cancer; peritoneal cancer; fallopian tube cancer	III/unknown status
NCT05919212	HER2	Blood	Trastuzumab deruxtecan	Breast cancer	II/recruiting
NCT01966471	HER2	Tumor tissue	Trastuzumab emtansine; trastuzumab; pertuzumab	Breast cancer	III/completed
NCT04044768	HLA‐A*02; MAGE‐A4	Tumor tissue	ADP‐A2M4	Synovial sarcoma; myxoid/round cell liposarcoma	II/active, not recruiting
NCT06472245	HLA‐A2	blood	OSE2101; docetaxel	NSCLC	III/recruiting
NCT06252649	KRAS G12c	Tumor tissue	Sotorasib	Colorectal cancer	III/recruiting
NCT07276880	PDL1	PDL1	Pembrolizumab	Triple‐negative breast cancer (TNBC)	II/not yet recruiting
NCT04486352	PIK3CA; AKT; PTEN	Tumor tissue	Ipatasertib/talazoparib/bevacizumab+atezolizumab; inavolisib + letrozole	Endometrial cancer	II/recruiting
NCT04101227	Serotonin	Blood	AD04; placebo	Alcohol use disorder	III/completed

### 5.3 Economic and Logistical Hurdles: Cost Effectiveness, Reimbursement, and Global Access

A significant factor impeding the widespread adoption of personalized medicines may stem from the heightened complexity of CDx devices and the corresponding pathways for therapeutic development and adoption [285]. Early in the process, multiple assays often need parallel evaluation to determine the most suitable test, increasing both cost and logistical burden [39]. The relevant research also indicates that people have a poor understanding of the financial value of IVD products, and there is also a mismatch between the regulatory cooperation requirements and the market‐driven incentives [250, 286]. Economic evaluations and cost‐effectiveness models have become increasingly significant components in guiding decisions related to market access, reimbursement, and coverage of emerging medical technologies [287]. However, the key challenge lies in balancing between patient benefits, economic value, and clinical value. Seo et al. found that many cost‐effectiveness studies of CDx‐guided therapies lacked reliable clinical outcome data [288]. Meanwhile, Doble et al. emphasized that it is necessary to incorporate the unique parameters of CDx into economic models to ensure the transparency and consistency of payment decisions [289]. Similarly, Montero et al. observed that the incorporation of CDx into health technology assessments is still evolving, with uncertain long‐term effects on decision timelines and reimbursement outcomes [287]. In a different study, Barnes et al. assessed the efficacy, safety, tolerability, and pricing of new anti‐cancer drugs, analyzing 74 studies encompassing 48,527 patients. Their findings indicated that immunotherapy displayed a superior safety and tolerability profile compared with other cancer therapies, and drug pricing did not correlate with efficacy [290].

From an international perspective, case studies of different national healthcare systems further demonstrate that reimbursement and pricing policies directly impact patients’ access to CDx and its corresponding targeted therapies. In the United States, while Medicare and commercial insurers typically cover high‐cost targeted drugs, they often hesitate to fully reimburse the essential CDx required for these therapies [291]. This financial imbalance limits actual patient access. In some European countries, there is a disconnect between reimbursement decisions for drug and CDx. This directly leads to the following consequence: even if a targeted drug is eligible for reimbursement, the required CDx may not be covered by the insurance, thereby affecting the accessibility of precision medicine [292]. In Australia, even when drugs paired with CDx receive timely approval, their diagnostic tests often face lengthy waits before inclusion in the reimbursement list. This disconnects between approval and reimbursement processes severely hinders their timely clinical application [293]. These examples collectively illustrate that the success of precision medicine hinges not only on its scientific efficacy but critically on whether healthcare policy systems can adapt promptly to address the novel economic and administrative challenges posed by the test–drug combination. While payers typically prioritize funding for high‐cost stratified therapies, they may ignore reimbursement for diagnostic tools that could reduce overall system expenditures [294]. Presently, decision‐making frameworks tailored for CDx are in the process of development for practical application by payer policy makers [295]. Additionally, Byron et al. delved into the challenges faced by health technology assessment bodies and policy makers when making reimbursement decisions concerning CDx. They emphasized the necessity for clear guidance from responsible agencies that assess economic models for new treatments, given the rapidly increasing volume of medicines accompanied by a CDx [296].

Additionally, the integration of gene panels into NGS clinical workflows introduces new cost and logistical complexities. Economic evaluations of CDx frequently overlook the broader impact that these tests have on the overall economic value of test–drug combinations. The issue of multiple testing must be thoughtfully addressed when employing NGS panel tests to maintain overall specificity, given that numerous genes are tested concurrently [297]. To realize the social benefits promised by NGS in the field of CDx, all stakeholders need to make a joint commitment. Cost‐effectiveness analyses of CDx tests for various targeted therapies in specialized cancer treatments have been widely conducted and documented in the literature. For instance, Schluckebier et al. evaluated the cost effectiveness of a particular examination using NGS compared with commonly used tests like RT‐PCR and FISH. Their results suggested that employing NGS for molecular diagnosis in patients with advanced‐stage NSCLC histology was not cost effective in terms of quality‐adjusted life years, especially from the perspective of the Brazilian supplementary health system [298]. Lim et al. conducted a cost‐effectiveness evaluation of tailoring treatment based on EGFR mutation status compared with the current no‐testing strategy in South Korea. The incremental cost‐effectiveness ratios rose as the number of patients to be treated with erlotinib increased, primarily due to the high cost of erlotinib [299]. Interestingly, Zaric et al. discovered that, in most cases analyzed, implementing precision medicine through CDx tests often led to an increase in medical costs. This finding contrasts with the implied promise of precision medicine, which suggests a reduction in healthcare costs [300]. Achieving the social benefits promised by NGS‐based CDx will therefore require coordinated collaboration among payers, policymakers, clinicians, and industry stakeholders [274, 301].

Ultimately, the goal of health economics and outcomes research are to facilitate decisions based on comprehensive information, in order to strike a balance between the therapeutic effects of patients and the utilization of health resources [302]. Addressing these challenges through integrated modeling, adaptive reimbursement policies, and cross‐departmental collaboration is crucial for realizing the full social and clinical value of precise diagnosis.

### 5.4 The Complexities of Drug–CDx Codevelopment

In the fields of drug development and precision medicine, a significant bottleneck lies in the translation process. This is particularly evident in the early stages of clinical development, and this phenomenon is commonly referred to as the translation gap [303, 304]. The advent of multiomics profiling, artificial intelligence (AI), and digital biomarkers has catalyzed the emergence of translational precision medicine, a concept aiming to optimize the entire process from drug discovery to clinical application [305, 306, 307]. These technological advances provide unprecedented opportunities to guide biomarker‐driven drug development. Current policies on the approval of CDx tests may inadvertently lead to inaccurate medical outcomes. Salgado et al. analyzed how existing policies governing CDx assay approvals may inadvertently contribute to imprecision in medicine and proposed a series of solutions aimed at addressing these challenges across multiple stakeholders, including the pharmaceutical and diagnostic industries, academia, patients, government bodies, and regulatory agencies [308]. One key recommendation was early engagement by all stakeholders in external quality control schemes to facilitate the swift development of guidelines and quality standards, ideally before regulatory agency approval is obtained. Through expert reviews using multiple CDx examples, the issues faced by the US FDA and the industry in terms of science, clinical practice, and regulation were clarified. At the same time, an in‐depth overview of the constantly changing regulatory environment in major markets was provided, and guidance was offered for the complexity of formulating effective global CDx product regulatory strategies [309, 310]. For CDx developers, the optimal approach is to design assay kits that meet the most stringent regulatory standards across major markets, with early and transparent communication among diagnostic and pharmaceutical partners and regulatory authorities being crucial to success. Kang et al. conducted a comparative analysis of regulatory agencies, including US FDA, the EMA, and the MFDS, to assess the need for strengthening the current clinical regulatory framework for CDx [311].

The drug‐diagnostic codevelopment model entails the simultaneous development of a drug alongside a predictive biomarker assay. This approach, coupled with a clinical enrichment trial design, has facilitated the creation of effective, target‐specific drugs tailored to molecular subgroups of cancer patients [312]. Karuri et al. proposed a two‐stage Bayesian design for the codevelopment of drugs and CDx assays, involving both marker‐positive and marker‐negative patients in a clinical trial setting [313]. Cotter et al. introduced the concept of the contract diagnostics organization, which serves as a business model offering pharmaceutical companies a comprehensive outsourcing partner to initiate and oversee the concurrent development of CDx tests in synergy with drug development efforts [314]. This expert review summarizes the key insights gleaned from pharmaceutical and diagnostic companies regarding the collaborative efforts necessary to support CDx development. These entities should align on aspects such as value, timelines, outcomes, and the impact of CDx development [315].

An illustrative instance of recent drug–CDx approval involves amivantamab (Rybrevant) alongside the NGS‐based Guardant360 CDx assay by Guardant Health. In the absence of broad industry‐wide cooperation, regulatory policies can be leveraged to reduce duplicative requirements and create a more favorable incentive structure for therapeutic and diagnostic companies pursuing targeted therapy and CDx development [316]. Another noteworthy example of CDx approval is the simultaneous the US FDA marketing clearance of the COBAS BRAF V600E test and vemurafenib (ZELBORAF) for metastatic melanoma. Clearly, the V600E mutation status is indispensable for the safe and effective use of vemurafenib (ZELBORAF) in metastatic melanoma. Recognizing this necessity, the sponsors employed a regulatory strategy that entailed the parallel development of the drug and its associated assay. The simultaneous approval of these two components can be regarded as a typical example of a jointly developed drug and diagnostic testing method [317].

Looking ahead, future CDx assays are likely to be composite assays comprising multiple biomarkers of diverse natures. These biomarkers will be combined using specific algorithms tailored to the drug or drugs they are intended to guide [318, 319]. To lower CDx costs, specific CDx quality systems and validation requirements could potentially be streamlined or postponed until the postapproval phase, contingent upon appropriate risk assessments [320]. Collectively, these examples underscore the multifaceted complexities encompassing scientific validation, regulatory coordination, and cross‐sector collaboration that continue to define the codevelopment of drugs and CDx assays.

## 6 Future Directions and Concluding Remarks

The future of CDx is being reshaped by factors such as intelligent technology, multiomics analysis, and data‐driven medicine. As precision medicine continues to evolve, the focus is shifting toward more integrated, automated, and predictive diagnostic frameworks. AI and machine learning (ML) are increasingly pivotal in building automated platforms for predictive and prognostic applications [321, 322]. This trend, together with advances in multiomics integration, AI‐enabled analysis, and an evolving regulatory landscape, underpins the strategic framework shaping the future of precision medicine.

### 6.1 The Rise of Multiomics and Integrated Diagnostic Platforms

Omics technologies provide a comprehensive and multilevel view of disease biology, enabling precise patient stratification, prediction of treatment response, and identification of biomarkers with clinical applications [323]. At the nucleic acid level, genomics systematically studies the structure, function, and variation of the complete genome of an organism through high‐throughput sequencing and bioinformatics methods, revealing the mechanisms of genetic regulation and the key genetic alterations associated with diseases [324]. Transcriptomics is the study of the complete RNA molecules or transcripts expressed by a particular cell, tissue, or organism under specific conditions [325]. Researchers can systematically understand gene expression patterns in different cell types, developmental stages, or environmental conditions, thus providing insights into the mechanisms of disease, drug targets, and the developmental processes of organisms. Proteomics captures functional disease states more directly than genomics [326]. Through proteomics testing, researchers can not only analyze the expression level of proteins, but also analyze their posttranslational modifications, structural changes, and interaction networks, thus revealing the molecular mechanisms of life activities more directly. Metabolomics studies the full spectrum of small‐molecule metabolites produced by a particular cell, tissue, or organism in a specific state, and it reflects both the physiological status of the cell and its immediate responses to environmental changes [327]. As the most dynamic layer of the molecular landscape, the metabolome can further characterize disease states and therapeutic responses, as well as reveal the spatial and temporal distribution of metabolites across tissues, organs, and biofluids [328, 329]. However, the single omics approach has obvious limitations as it can only study cells or organisms at a single level, unable to reveal the functions of the entire organism, nor can it clarify the relationship between the organism and environmental factors.

Integration of genomics, transcriptomics, proteomics, and metabolomics helps to elucidate disease mechanisms at a systemic level and facilitates the discovery of composite biomarkers that reflect dynamic changes in the intrinsic properties of tumors and their microenvironments [330, 331]. Multiomics strategies have demonstrated robust systems analysis capabilities across various disease models. For instance, a multiomics study of metabolic dysfunction‐associated steatitis liver disease combined proteomics, transcriptomics, single‐cell RNA sequencing, and clinical data to systematically investigate disease mechanisms [332]. The study identified key circulating biomarkers, including TREM2 and LAGLS3BP. These biomarkers, integrated with clinical parameters, were used to construct the ABD‐LTyG model for identifying high‐risk MASH. The model achieved AUC of 0.832 and 0.807 in discovery and validation cohorts, outperforming conventional noninvasive scores. Analysis revealed the pivotal role of Kupffer cell‐associated immune–metabolic pathways in disease progression, providing novel insights for the precise early diagnosis of high‐risk MASH. In oncology, multiomics integration has also advanced the stratified understanding of complex disease phenotypes. Another study on breast cancer and multiple primary malignancies employed integrated quantitative proteomics and radiomics to deeply analyze molecular differences across tumor subtypes [333]. Proteomics revealed distinct pathway enrichments, with cell proliferation pathways prominent in solitary breast cancer, estrogen receptor signaling in multiple primary breast cancers, and immune response pathways in multiple primary lung cancers. Radiomics demonstrated good discriminatory power (CC‐Radscore AUC = 0.856, MLO‐Radscore AUC = 0.824). This study presents an innovative approach by performing multidimensional analysis, spanning from molecular levels to imaging phenotypes. It not only uncovers tumor molecular characteristics but also establishes meaningful connections between molecular and immune subtypes and their corresponding imaging features. Moreover, a multidisciplinary approach integrating innovative diagnostic tools, novel therapeutic regimens, and AI‐driven solutions is crucial for the advancement of CDx, as exemplified by liver cirrhosis [334]. This has the potential to enhance precision in diagnosis and risk stratification. As multiomics datasets expand, AI‐assisted decision frameworks become essential for translating complex molecular patterns into clinically deployable CDx algorithms.

### 6.2 AI and ML in CDx Data Interpretation

AI and ML are emerging as key drivers of precision medicine in the field of CDx. Leveraging technologies such as ML, deep learning, natural language processing, and computer vision, AI enables smarter processing of medical data, thereby enhancing the accuracy of disease diagnosis, risk stratification, and treatment response prediction [335]. Algorithmic improvements also enhance staining consistency and analytical efficiency. Leo et al. introduced a nondestructive tissue analysis method, termed histotyping, which offers automated prognosis for biochemical recurrence in prostate cancer [336]. This method relies on computational image analysis of morphologic patterns within prostate tissue, extracted from a single, routinely acquired hematoxylin and eosin slide. The development of AI in CDx does not necessarily depend on sophisticated deep learning architectures. With thoughtful integration and optimization, traditional algorithms can also deliver significant performance improvements in targeted applications. This study demonstrates that combining RGB multichannel differential edge detection operators in cytological image analysis can significantly improve efficiency, particularly offering advantages when processing samples with uneven staining [337].

A primary application is the quantitative analysis of IHC. For instance, algorithms trained on whole‐slide images assist detection and scoring of clinically relevant biomarkers such as HER2, PD‐L1, and Ki‐67, reducing interobserver variability relative to manual reads [338]. Specifically for PD‐L1, several studies have shown that AI‐assisted quantification of the tumor proportion score or CPS demonstrates strong concordance with expert pathologists, while also enhancing reproducibility across various assays and cohorts [339]. Notably, the US FDA granted Breakthrough Device Designation to the VENTANA TROP2 RxDx in 2025, the first computational pathology CDx, enabling precise quantification of TROP2 expression in NSCLC [340]. Traditional algorithms perform well with small‐sample, high‐dimensional data, while deep learning models demonstrate accuracy approaching or even surpassing expert levels in medical image recognition. However, model interpretability, clinical validation, and regulatory approval remain key challenges for future development.

AI exhibits strong capabilities in mining complex biomarker patterns from high‐dimensional, multimodal datasets, providing a foundation for large‐scale cross‐cohort analyses [341]. In biomarker exploration, He et al. analyzed circulating immune‐related miRNAs (cf‐IRmiRNAs) across 15,832 samples and constructed a cross‐cancer diagnostic model using the XGBoost algorithm [342]. This model incorporates 39 key miRNAs and demonstrates outstanding performance (AUC = 0.984) across multiple datasets, distinguishing early‐stage cancers from benign lesions. This underscores the immense potential of AI‐driven models in enabling noninvasive, pan‐cancer early screening. More broadly, ML enables automated feature extraction from multimodal data, including radiomics, molecular omics, and liquid biopsy profiles to support early cancer detection, pathological diagnosis, risk stratification, and treatment response prediction [343]. Yang et al. analyzed 9692 CTC images and over 25 million white blood cell images from 1703 patients [344]. They extracted 57 cellular features and constructed a CTC recognition model using ML. The model demonstrated high accuracy in CTC identification, with an AUC–ROC greater than 0.99 and an AUC–PR of 0.869. It achieved automated recognition and classification of CTC, providing a technical pathway for standardizing and automating tumor microfluidic liquid biopsy. Multimodal fusion models integrate imaging, genomic, and clinical information to achieve more precise personalized diagnosis and treatment [345].

Overall, the rapid advancement of AI and ML is driving the shift of CDx from manual experience‐driven approaches to data intelligence‐driven methodologies. In the future, AI models integrating multiomics data, radiomics, and clinical information will play a pivotal role in precision oncology and personalized therapy [346].

### 6.3 Toward Companion Therapeutics and More Dynamic Monitoring

CDx is evolving from a traditional pretreatment screening tool into a new paradigm that integrates CDx with real‐time monitoring. This transformation fundamentally fulfills the core requirements of precision medicine for real‐time responsiveness and precise regulation, shifting the diagnostic and therapeutic relationship from linear decision‐making to a closed‐loop feedback system. The core concept of companion therapeutics (CTx) lies in deeply integrating the therapeutic process with real‐time diagnostics. Through technologies such as molecular imaging, liquid biopsy, and multiomics testing, it enables the visualization and quantification of dynamic disease changes. Lin et al. first clearly defined CTx as an integrated approach combining therapy with molecular imaging to guide treatment decisions, allowing simultaneous assessment of therapeutic response and adjustment of interventions based on molecular readouts [347]. By constructing a high‐precision detection–treatment‐feedback loop, CTx overcomes the limitations of traditional IVD in capturing tumor heterogeneity.

Clinical examples illustrate the potential of this approach. The ZEPHIR trial employed ^8^ ^9^Zr–trastuzumab PET imaging to assess HER2 expression in breast cancer patients, successfully predicting their response to T‐DM1 targeted therapy [348]. Concurrently, the NCT05000372 trial employed ^6^ ^8^Ga–grazytracer PET imaging to monitor real‐time changes in granzyme B following immunotherapy, enabling noninvasive differentiation between responders and nonresponders [349]. Lin et al. further developed the LET‐1052 photothermal‐responsive probe, which enhances fluorescence signals after treatment by increasing intracellular viscosity. The intensity of these signals positively correlates with tumor suppression efficacy, enabling real‐time self‐assessment following therapy [350].

At the molecular monitoring level, FoundationOne Liquid CDx analyzes over 300 cancer‐related genes from blood samples, identifying rare mutations such as MET exon 14 skipping mutations while dynamically capturing clonal evolution during treatment [351, 352]. In China, suvotinib for NSCLC patients with EGFR exon 20 insertions, combined with the Lank CDx multigene panel, enables continuous monitoring of EGFR and MET mutations throughout treatment, achieving an objective response rate of 61% [353]. These examples fully demonstrate the clinical value of an integrated diagnostics and treatment strategies.

Despite these advances, the implementation of CTx and dynamic monitoring still faces several challenges. Tumor spatiotemporal heterogeneity complicates biomarker identification, while the sensitivity of imaging probes and detection reagents requires further optimization. Additionally, regulatory pathways for simultaneous development of diagnostics and therapeutics need refinement to balance innovation with safety. Nevertheless, with the integration of emerging technologies such as organoid‐based functional testing and real‐time imaging probes, it is foreseeable that CDx will evolve from merely guiding drug use to actively regulating treatment, thereby establishing a more precise and dynamic response system for personalized medicine.

### 6.4 The Future Regulatory and Healthcare Ecosystem for CDx

Currently, most major global markets predominantly follow the one drug, one test CDx regulatory model, designed to ensure patient safety and treatment consistency through rigorous verification of both the analytical performance and clinical validity of diagnostic tests. Since approving the first CDx product in 1998, the United States has clearly defined CDx, established classification criteria, and outlined requirements for codevelopment with drugs, emphasizing their role in ensuring patient medication safety, efficacy, and drug synergistic effects. The EU has established a comprehensive regulatory framework for IVD medical devices, covering design, manufacturing, and postmarket surveillance. China, Japan, Korea, and other countries have also developed corresponding regulatory policies tailored to their national contexts to ensure the quality, clinical safety, and efficacy of CDx [35].

The regulatory framework in China is increasingly aligning with international standards. Since 2014, national drug regulatory authorities have progressively refined policies for CDx, evolving from the CFDA early exploration of clinical trial regulations to the 2017 reform guidelines encouraging simultaneous development of drugs and diagnostic reagents, and culminating in the NMPA's 2018 release of the guidance principles. This gradual development has established a systemic framework to support standardized R&D and clinical translation of CDx. At the same time, payment reforms have significantly improved CDx accessibility. Some regions now include certain CDx tests for common cancers, such as lung and breast cancer in medical insurance coverage. Likewise, the United States and Europe have expanded CDx access through commercial insurance or government subsidies, reducing the financial burden on patients and stimulating growth in testing demand.

Looking ahead, the regulatory and healthcare ecosystem for CDx will enter a new phase characterized by data‐driven approaches, model‐assisted decision‐making, and global collaboration. Regulatory frameworks will transition from static approvals to dynamic management, becoming more flexible and open while deeply relying on digital evidence and big data models for decision‐making and risk assessment. Cross‐platform consistency studies and multicenter data validation will ensure comparability and mutual recognition of test results. For AI‐driven CDx, regulators will strengthen algorithm transparency and performance monitoring to guarantee model robustness and fairness in real‐world data. Approval models will gradually transition from one drug per test to one test per drug class or multiple drugs per shared test, replacing redundant approvals with minimum performance standards to enhance innovation efficiency. Simultaneously, real‐world evidence will become a critical basis for regulatory re‐evaluation and healthcare financing decisions through multicenter data sharing networks built on electronic health records, genomic databases, and patient registries. For AI‐CDx and multiomics diagnostic products, regulators will explore continuous learning approval mechanisms, enabling dynamic oversight through model update filings and performance threshold monitoring. Future payment systems will also deeply integrate with big data evaluation models, advancing value‐based precision medicine payment models. The US FDA is piloting initiatives to replace traditional approvals with minimum performance standards and data sharing, signaling that global regulators, pharmaceutical companies, and healthcare institutions are jointly building an open, interconnected, and sustainable precision medicine ecosystem centered on big data‐driven regulatory science. This approach accelerates the clinical translation and widespread adoption of innovative outcomes while ensuring safety and efficacy.

### 6.5 Conclusion

The development, potential, and growth of CDx assays hold promise for further personalizing therapeutic strategies and enhancing patient access, outcomes, and responses to innovative pharmaceutical agents or diagnostic methods. CDx has witnessed extensive utilization, notably in the realm of oncology therapies, aiding in the identification of patient cohorts where therapeutic products have demonstrated both safety and efficacy. This adaptability allows for treatment modifications with the aim of achieving optimal safety profiles. Undoubtedly, soon, NGS‐based CDx will emerge as a pivotal component within the highly profitable domains of precision medicine and personalized healthcare. A cornerstone of CDx evolution lies in the identification of biomarkers, an indispensable factor in its development. By leveraging diverse biomarker profiles, CDx has been extensively applied in cancer therapy to accurately delineate patient cohorts suitable for specific therapeutic interventions. This personalized approach has resulted in improved therapeutic outcomes while reducing unnecessary healthcare expenses. Moreover, the widespread adoption of CDx is poised to yield substantial economic benefits, positively impacting healthcare economics and public health. CDx, by reducing trial and error in treatment decisions, aids in diminishing superfluous medical costs. Accurate diagnoses and tailored treatments optimize the allocation of medical resources, offering more cost‐effective, accessible, and superior healthcare services to patients. Nevertheless, the CDx field faces its share of challenges and complexities necessitating collaborative solutions. Beyond technical and regulatory challenges, patient‐centered factors are equally critical to the successful implementation of CDx. Patient acceptance and understanding of CDx testing directly influence its real‐world application, while genetic counseling empowers patients to make informed decisions, alleviates psychological burdens, and helps them comprehend complex genomic results. Integrating these patient‐related factors into the CDx process will further enhance the clinical value and societal benefits of precision medicine. These challenges encompass the precise identification of biomarkers, continual technological advancements, intricacies of clinical trial design, and adherence to governmental regulatory policies. To surmount these hurdles, pharmaceutical and diagnostic companies must fortify their collaborations to drive innovation in CDx technology. Concurrently, governments and regulatory bodies should develop transparent policies that provide support and guidance to ensure the safe and effective deployment of CDx technologies, ultimately benefiting patients. Through collective endeavors, there is growing confidence in overcoming these challenges, enabling CDx to assume an even more significant role in the future, delivering precision and personalized healthcare to patients.

## Author Contributions

M. Yin, X. Li, T. Liu, and Z.‐S. Chen made substantial contributions to conception and design. X. Luo, Z. Zhang, and J. Liao prepared all the references and classified them. Y. Wu and R. Xue wrote the first draft. T. Liu, X. Li, and M. Yin mainly revised this manuscript. Y. Wu, R. Xue, and J. Deng drew the figures and tables. Y. Wu, Z.‐S.C., and X. Li made revisions of this review. All authors contributed to the article and approved the submitted version.

## Funding

This work was supported by National Key R&D Programmes (NKPs) of China (Grant No. 2022YFC3601802), the Chongqing Medical Scientific Research Project (Joint Project of Chongqing Health Commission and Science and Technology Bureau) (Grant No. 2024QNXM004), the Chongqing Wanzhou District PhD Direct‐Access Research Project (Grant No. wzstc‐20240006), The Science and Technology Research Program of Chongqing Municipal Education Commission (Grant No. KJZD‐K202300105), The Natural Science Foundation of Chongqing (Grant No. CSTB2024NSCQ‐MSX0874, Grant No. CSTB2024NSCQ‐MSX0761, and No. CSTB2024NSCQ‐MSX1595), and the Chongqing Science and Health Joint Scientific Research Project on Traditional Chinese Medicine (No. 2024ZYQN001).

## Ethics Statement

The authors have nothing to report.

## Conflicts of Interest

Author Zhe‐Sheng Chen is an Editorial board member of MedComm. Author Zhe‐Sheng Chen was not involved in the journal's review or decisions related to this manuscript. The other authors declared no conflict of interest.

## Supporting information




**Supporting Table 1**: The major differences of CDx regulation policies between FDA, EMA, NMPA, MFDS, and PMDA.

## Data Availability

The authors have nothing to report.

## References

[1] F. Beccia , I. Hoxhaj , C. Castagna , et al., “An Overview of Personalized Medicine Landscape and Policies in the European Union,” European Journal of Public Health 32, no. 6 (2022): 844–851. 36305782 10.1093/eurpub/ckac103 PMC9713394

[2] O. Strianese , F. Rizzo , M. Ciccarelli , et al., “Precision and Personalized Medicine: How Genomic Approach Improves the Management of Cardiovascular and Neurodegenerative Disease,” Genes 11, no. 7 (2020): 747. 32640513 10.3390/genes11070747 PMC7397223

[3] G. M. Sprinzl and A. Magele , “Personalized Medicine in Otolaryngology: Special Topic Otology,” Journal of Personalized Medicine 12, no. 11 (2022): 1820. 36579531 10.3390/jpm12111820 PMC9697707

[4] S. Kunte , J. Abraham , and A. J. Montero , “Novel HER2‐targeted Therapies for HER2‐positive Metastatic Breast Cancer,” Cancer 126, no. 19 (2020): 4278–4288. 32721042 10.1002/cncr.33102

[5] J. T. Jørgensen and J. Mollerup , “Companion Diagnostics and Predictive Biomarkers for MET‐Targeted Therapy in NSCLC,” Cancers (Basel) 14, no. 9 (2022): 2150. 35565287 10.3390/cancers14092150 PMC9105764

[6] V. Valla , S. Alzabin , A. Koukoura , et al., “Companion Diagnostics: State of the Art and New Regulations,” Biomarker Insights 16 (2021): 11772719211047763. 34658618 10.1177/11772719211047763 PMC8512279

[7] E. Csoke , S. Landes , M. J. Francis , et al., “How Can Real‐world Evidence Aid Decision Making during the Life Cycle of Nonprescription Medicines?,” Clinical and Translational Science 15, no. 1 (2022): 43–54. 34405554 10.1111/cts.13129 PMC8742642

[8] C. P. Milne , C. Bryan , S. Garafalo , et al., “Complementary versus Companion Diagnostics: Apples and Oranges?,” Biomarkers in Medicine 9, no. 1 (2015): 25–34. 25605453 10.2217/bmm.14.84

[9] J. J. Gao , C. L. Osgood , Y. Gong , et al., “FDA Approval Summary: Pertuzumab, Trastuzumab, and Hyaluronidase‐zzxf Injection for Subcutaneous Use in Patients With HER2‐positive Breast Cancer,” Clinical Cancer Research: An Official Journal of the American Association for Cancer Research 27, no. 8 (2021): 2126–2129. 33188141 10.1158/1078-0432.CCR-20-3474

[10] T. Phillips , M. M. Millett , X. Zhang , et al., “Development of a Diagnostic Programmed Cell Death 1‐Ligand 1 Immunohistochemistry Assay for Nivolumab Therapy in Melanoma,” Applied Immunohistochemistry & Molecular Morphology: AIMM 26, no. 1 (2018): 6–12. 29189265 10.1097/PAI.0000000000000605 PMC5753812

[11] J. T. Jørgensen , “Companion and Complementary Diagnostics: An Important Treatment Decision Tool in Precision Medicine,” Expert Review of Molecular Diagnostics 20, no. 6 (2020): 557–559. 32342707 10.1080/14737159.2020.1762573

[12] J. C. Stingl , “Pharmacogenetic Biomarkers for Companion and Complementary Diagnostics: Challenges for Clinical Practice and Regulation,” Expert Review of Precision Medicine and Drug Development 1, no. 5 (2016): 415–418.

[13] H. Scheerens , A. Malong , K. Bassett , et al., “Current Status of Companion and Complementary Diagnostics: Strategic Considerations for Development and Launch,” Clinical and Translational Science 10, no. 2 (2017): 84–92. 28121072 10.1111/cts.12455 PMC5355969

[14] H. Hampel , P. Gao , J. Cummings , et al., “The Foundation and Architecture of Precision Medicine in Neurology and Psychiatry,” Trends in Neurosciences 46, no. 3 (2023): 176–198. 36642626 10.1016/j.tins.2022.12.004 PMC10720395

[15] A. A. Baumann , Z. Buribayev , O. Wolkenhauer , et al., “Epigenomic Echoes‐Decoding Genomic and Epigenetic Instability to Distinguish Lung Cancer Types and Predict Relapse,” Epigenomes 9, no. 1 (2025): 5. 39982247 10.3390/epigenomes9010005 PMC11843950

[16] K. T. Schmidt , C. H. Chau , D. K. Price , et al., “Precision Oncology Medicine: The Clinical Relevance of Patient‐Specific Biomarkers Used to Optimize Cancer Treatment,” Journal of Clinical Pharmacology 56, no. 12 (2016): 1484–1499. 27197880 10.1002/jcph.765 PMC5112148

[17] P. Keeling , J. Clark , and S. Finucane , “Challenges in the Clinical Implementation of Precision Medicine Companion Diagnostics,” Expert Review of Molecular Diagnostics 20, no. 6 (2020): 593–599. 32336167 10.1080/14737159.2020.1757436

[18] T. B. G. Poulsen , A. Karamehmedovic , C. Aboo , et al., “Protein Array‐based Companion Diagnostics in Precision Medicine,” Expert Review of Molecular Diagnostics 20, no. 12 (2020): 1183–1198. 33315478 10.1080/14737159.2020.1857734

[19] M. R. Campbell , “Update on Molecular Companion Diagnostics—A Future in Personalized Medicine Beyond Sanger Sequencing,” Expert Review of Molecular Diagnostics 20, no. 6 (2020): 637–644. 32167388 10.1080/14737159.2020.1743177

[20] N. Papadopoulos , K. W. Kinzler , and B. Vogelstein , “The Role of Companion Diagnostics in The Development and Use of Mutation‐Targeted Cancer Terapies,” Nature biotechnology 24, no. 8 (2006): 985–995. 10.1038/nbt1234 16900147

[21] I. A. Cree , “Diagnostic Ras Mutation Analysis by Polymerase Chain Reaction (PCR),” Biomolecular Detection and Quantification 8 (2016): 29–32. 27335808 10.1016/j.bdq.2016.05.001 PMC4906127

[22] J. T. Jørgensen , “Clinical Application of Companion Diagnostics,” Trends in Molecular Medicine 21, no. 7 (2015): 405–407. 26141798 10.1016/j.molmed.2015.05.003

[23] M. Kaufmann , M. Keppens , and E. D. Blair , “A Perspective Analysis: Companion Diagnostics: An Evolving Paradigm in 21st Century Healthcare,” Personalized Medicine 12, no. 4 (2015): 389–402. 29771658 10.2217/pme.15.2

[24] J. T. Jørgensen , “A Changing Landscape for Companion Diagnostics,” Expert Review of Molecular Diagnostics 13, no. 7 (2013): 667–669. 24063394 10.1586/14737159.2013.834799

[25] Y. Hu , Y. Sun , Q. H. Zhu , et al., “Poaceae Chloroplast Genome Sequencing: Great Leap Forward in Recent Ten Years,” Current Genomics 23, no. 6 (2023): 369–384. 37920556 10.2174/1389202924666221201140603 PMC10173419

[26] T. C. Chou , L. You , C. Beerens , et al., “Instant Processing of Large‐scale Image Data With FACT, a Real‐time Cell Segmentation and Tracking Algorithm,” Cell Reports Methods 3, no. 11 (2023): 100636. 37963463 10.1016/j.crmeth.2023.100636 PMC10694492

[27] H. Li , S. E. Huang , C. L. Geng , et al., “Comprehensive Analysis Reveals Hub Genes Associated With Immune Cell Infiltration in Allergic Rhinitis,” World Journal of Otorhinolaryngology—Head and Neck Surgery 9, no. 4 (2023): 340–351. 38059138 10.1002/wjo2.92 PMC10696276

[28] D. Olsen and J. T. Jørgensen , “Companion Diagnostics for Targeted Cancer Drugs—Clinical and Regulatory Aspects,” Frontiers in Oncology 4 (2014): 105. 24904822 10.3389/fonc.2014.00105 PMC4032883

[29] FDA . Companion Diagnostics 2023, https://www.fda.gov/medical‐devices/in‐vitro‐diagnostics/companion‐diagnostics.

[30] FDA . Oncology Drug Products Used with Certain In Vitro Diagnostic Tests: Pilot Program 2023, https://www.fda.gov/regulatory‐information/search‐fda‐guidance‐documents/oncology‐drug‐products‐used‐certain‐in‐vitro‐diagnostic‐tests‐pilot‐program.

[31] F. Pignatti , F. Ehmann , R. Hemmings , et al., “Cancer Drug Development and the Evolving Regulatory Framework for Companion Diagnostics in the European Union,” Clinical Cancer Research: An Official Journal of the American Association for Cancer Research 20, no. 6 (2014): 1458–1468. 24634469 10.1158/1078-0432.CCR-13-1571

[32] L. P. Orellana García , F. Ehmann , and P. A. Hines , “Biomarker and Companion Diagnostics‐A Review of Medicinal Products Approved by the European Medicines Agency,” Frontiers in Medicine 8 (2021): 753187. 34790681 10.3389/fmed.2021.753187 PMC8591033

[33] V. Wurcel , O. Perche , D. Lesteven , et al., “The Value of Companion Diagnostics: Overcoming Access Barriers to Transform Personalised Health Care Into an Affordable Reality in Europe,” Public Health Genomics 19, no. 3 (2016): 137–143. 27237134 10.1159/000446531

[34] A. M. Senderowicz and O. Pfaff , “Similarities and Differences in the Oncology Drug Approval Process Between FDA and European Union With Emphasis on in Vitro Companion Diagnostics,” Clinical Cancer Research: An Official Journal of the American Association for Cancer Research 20, no. 6 (2014): 1445–1452. 24634467 10.1158/1078-0432.CCR-13-1761

[35] K. S. Oliner , M. Shiller , P. Schmid , et al., “Challenges to Innovation Arising From Current Companion Diagnostic Regulations and Suggestions for Improvements,” Clinical Cancer Research: An Official Journal of the American Association for Cancer Research 31, no. 5 (2025): 795–800. 39724199 10.1158/1078-0432.CCR-24-2729 PMC11873800

[36] E. Y. Lee and H. C. Shen , “Regulatory Considerations for Companion Diagnostic Devices,” Biomarkers in Medicine 9, no. 1 (2015): 67–75. 25605456 10.2217/bmm.14.98

[37] T. Golan , M. Milella , A. Ackerstein , et al., “The Changing Face of Clinical Trials in the Personalized Medicine and Immuno‐oncology Era: Report From the International Congress on Clinical Trials in Oncology & Hemato‐Oncology (ICTO 2017),” Journal of Experimental & Clinical Cancer Research 36, no. 1 (2017): 192. 29282151 10.1186/s13046-017-0668-0 PMC5745625

[38] M. Li , “Statistical Consideration and Challenges in Bridging Study of Personalized Medicine,” Journal of Biopharmaceutical Statistics 25, no. 3 (2015): 397–407. 24897254 10.1080/10543406.2014.920340

[39] J. T. Jørgensen , S. Møller , B. B. Rasmussen , et al., “High Concordance Between Two Companion Diagnostics Tests: A Concordance Study Between the HercepTest and the HER2 FISH PharmDx Kit,” American Journal of Clinical Pathology 136, no. 1 (2011): 145–151. 21685042 10.1309/AJCPJPJ8ZWGDTTWC

[40] A. Ritzhaupt , I. Hayes , and F. Ehmann , “Implementing the EU in Vitro Diagnostic Regulation—A European Regulatory Perspective on Companion Diagnostics,” Expert Review of Molecular Diagnostics 20, no. 6 (2020): 565–567. 31976775 10.1080/14737159.2020.1720653

[41] EMA . Regulation (EU) 2017/746 of the European Parliament and of the Council of 5 April 2017 on in vitro diagnostic medical devices and repealing Directive 98/79/EC and Commission Decision 2010/227/EU (Text with EEA relevance.) 2017, https://eur‐lex.europa.eu/legal‐content/EN/TXT/?uri=CELEX%3A32017R0746.

[42] (MFDS) MoFaDS . Act on In Vitro Diagnostic Medical Devices Sejong, Korea 2020, https://www.mfds.go.kr/eng/brd/m_75/view.do?company_cd&company_nm&itm_seq_1=0&itm_seq_2=0&multi_itm_seq=0&page=1&seq=4&srchFr&srchTo&srchTp&srchWord&utm_source=chatgpt.com.

[43] Japan Go . Act on Securing Quality, Efficacy and Safety of Products Including Pharmaceuticals and Medical Devices Tokyo (Japan: Japanese Law Translation, 2014), https://www.japaneselawtranslation.go.jp/en/laws/view/3213/en.

[44] A. Tanaka , H. Suzuki , S. Toyoshima , et al., “Co‐Development of Oncology Drugs and Companion Diagnostics: Analyses of Approval Lags and Drug Development Periods in Recently Approved Cases in Japan,” Therapeutic Innovation & Regulatory Science 56, no. 1 (2022): 85–95. 34406635 10.1007/s43441-021-00332-6

[45] P. Kukk , E. H. M. Moors , and M. P. Hekkert , “The Complexities in System Building Strategies—The Case of Personalized Cancer Medicines in England,” Technological Forecasting and Social Change 98 (2015): 47–59.

[46] C. G. Ferreira , M. I. Achatz , P. Ashton‐Prolla , et al., “Brazilian Health‐care Policy for Targeted Oncology Therapies and Companion Diagnostic Testing,” The Lancet Oncology 17, no. 8 (2016): e363–e370. 27511160 10.1016/S1470-2045(16)30171-1

[47] K. Mistry , S. Sadarangani , D. Moreno , et al., “Novel Biomarkers and Imaging Tests for Acute Kidney Injury Diagnosis in Patients With Cancer,” Kidney360 6, no. 1 (2025): 167–174. 39575585 10.34067/KID.0000000660 PMC11793192

[48] S. Cheng , W. H. Koch , and L. Wu , “Co‐development of a Companion Diagnostic for Targeted Cancer Therapy,” New Biotechnology 29, no. 6 (2012): 682–688. 22391147 10.1016/j.nbt.2012.02.002

[49] M. S. Boguski and M. W. McIntosh , “Biomedical Informatics for Proteomics,” Nature 422, no. 6928 (2003): 233–237. 12634797 10.1038/nature01515

[50] M. W. Moore , D. Babu , and P. D. Cotter , “Challenges in the Codevelopment of Companion Diagnostics,” Personalized Medicine 9, no. 5 (2012): 485–496. 29768776 10.2217/pme.12.60

[51] A. Ocana , J. L. Ethier , L. Díez‐González , et al., “Influence of Companion Diagnostics on Efficacy and Safety of Targeted Anti‐cancer Drugs: Systematic Review and Meta‐Analyses,” Oncotarget 6, no. 37 (2015): 39538–39549. 26446908 10.18632/oncotarget.5946 PMC4741844

[52] J. T. Jørgensen , “Companion Diagnostics: The Key to Personalized Medicine. Foreword,” Expert Review of Molecular Diagnostics 15, no. 2 (2015): 153–156. 25597758 10.1586/14737159.2015.1002470

[53] C. Schmidt , “Challenges Ahead for Companion Diagnostics,” Journal of the National Cancer Institute 104, no. 1 (2012): 14–15. 22173588 10.1093/jnci/djr535

[54] Y. S. Fan , “Companion Diagnostic Testing for Targeted Cancer Therapies: An Overview,” Genetic Testing and Molecular Biomarkers 17, no. 7 (2013): 515–523. 23574530 10.1089/gtmb.2012.0510

[55] D. A. Murphy , H. A. Ely , R. Shoemaker , et al., “Detecting Gene Rearrangements in Patient Populations through a 2‐Step Diagnostic Test Comprised of Rapid IHC Enrichment Followed by Sensitive Next‐Generation Sequencing,” Applied Immunohistochemistry & Molecular Morphology: AIMM 25, no. 7 (2017): 513–523. 27028240 10.1097/PAI.0000000000000360 PMC5553231

[56] R. Tenchov , A. K. Sapra , J. Sasso , et al., “Biomarkers for Early Cancer Detection: A Landscape View of Recent Advancements, Spotlighting Pancreatic and Liver Cancers,” ACS Pharmacol Transl Sci 7, no. 3 (2024): 586–613. 38481702 10.1021/acsptsci.3c00346 PMC10928905

[57] N. Nevler , D. Niehoff , A. M. Gleixner , et al., “Developing Digital Health Technologies for Frontotemporal Degeneration,” Alzheimer's & Dementia: The Journal of the Alzheimer's Association 21, no. 4 (2025): e70082. 10.1002/alz.70082 PMC12032191 40285380

[58] H. C. Wang , T. J. Yeh , L. P. Chan , et al., “Exploration of Feasible Immune Biomarkers for Immune Checkpoint Inhibitors in Head and Neck Squamous Cell Carcinoma Treatment in Real World Clinical Practice,” International Journal of Molecular Sciences 21, no. 20 (2020): 7621. 33076306 10.3390/ijms21207621 PMC7589088

[59] R. Simon , “Sensitivity, Specificity, PPV, and NPV for Predictive Biomarkers,” Journal of the National Cancer Institute 107, no. 8 (2015): djv153. 26109105 10.1093/jnci/djv153 PMC4609556

[60] S. Liao , M. Zhou , Y. Wang , et al., “Emerging Biomedical Imaging‐based Companion Diagnostics for Precision Medicine,” iScience 26, no. 8 (2023): 107277. 37520706 10.1016/j.isci.2023.107277 PMC10371849

[61] C. Huber , T. Friede , J. Stingl , et al., “Classification of Companion Diagnostics: A New Framework for Biomarker‐Driven Patient Selection,” Therapeutic Innovation & Regulatory Science 56, no. 2 (2022): 244–254. 34841493 10.1007/s43441-021-00352-2 PMC8854277

[62] B. J. Min , W. S. Lee , M. E. Seo , et al., “Development and Validation of Targeted Gene Sequencing Panel Based Companion Diagnostic for Korean Patients With Solid Tumors,” Cancers (Basel) 13, no. 20 (2021): 5112. 34680263 10.3390/cancers13205112 PMC8534153

[63] D. E. Carvajal‐Hausdorf , K. A. Schalper , V. M. Neumeister , et al., “Quantitative Measurement of Cancer Tissue Biomarkers in the Lab and in the Clinic,” Laboratory Investigation; A Journal of Technical Methods and Pathology 95, no. 4 (2015): 385–396. 25502176 10.1038/labinvest.2014.157 PMC4383674

[64] Y. Cao , R. Agarwal , F. Dituri , et al., “NGS‐based Transcriptome Profiling Reveals Biomarkers for Companion Diagnostics of the TGF‐β Receptor Blocker Galunisertib in HCC,” Cell Death & Disease 8, no. 2 (2017): e2634. 28230858 10.1038/cddis.2017.44 PMC5386488

[65] E. Conde , S. Hernandez , M. Prieto , et al., “Profile of Ventana ALK (D5F3) Companion Diagnostic Assay for Non‐small‐cell Lung Carcinomas,” Expert Review of Molecular Diagnostics 16, no. 6 (2016): 707–713. 27031368 10.1586/14737159.2016.1172963

[66] N. B. La Thangue and D. J. Kerr , “Predictive Biomarkers: A Paradigm Shift towards Personalized Cancer Medicine,” Nature Reviews Clinical Oncology 8, no. 10 (2011): 587–596. 10.1038/nrclinonc.2011.121 21862978

[67] I. A. Cree , “Progress and Potential of RAS Mutation Detection for Diagnostics and Companion Diagnostics,” Expert Review of Molecular Diagnostics 16, no. 10 (2016): 1067–1072. 27494709 10.1080/14737159.2016.1221345

[68] T. A. Yap , S. K. Sandhu , P. Workman , et al., “Envisioning the Future of Early Anticancer Drug Development,” Nature Reviews Cancer 10, no. 7 (2010): 514–523. 20535131 10.1038/nrc2870

[69] V. S. Nikam . Companion Diagnostics and Clinical Biomarkers for Immunotherapy. in Immunotherapy – A Novel Facet of Modern Therapeutics, ed. S. P. Sawarkar , V. S. Nikam , and S. Syed (Singapore: Springer Singapore, 2021): 137–152.

[70] A. Tibau , L. Díez‐González , B. Navarro , et al., “Impact of Availability of Companion Diagnostics on the Clinical Development of Anticancer Drugs,” Molecular Diagnosis & Therapy 21, no. 3 (2017): 337–343. 28247182 10.1007/s40291-017-0267-y

[71] D. R. Parkinson , B. E. Johnson , and G. W. Sledge , “Making Personalized Cancer Medicine a Reality: Challenges and Opportunities in the Development of Biomarkers and Companion Diagnostics,” Clinical Cancer Research: An Official Journal of the American Association for Cancer Research 18, no. 3 (2012): 619–624. 22298894 10.1158/1078-0432.CCR-11-2017

[72] M. K. Le , N. Oishi , K. Mochizuki , et al., “Immunohistochemical Detection of Cancer Genetic Abnormalities,” Pathology, Research and Practice 255 (2024): 155109. 38340581 10.1016/j.prp.2024.155109

[73] L. Hu , K. Ru , L. Zhang , et al., “Fluorescence in Situ Hybridization (FISH): An Increasingly Demanded Tool for Biomarker Research and Personalized Medicine,” Biomarker Research 2, no. 1 (2014): 3. 24499728 10.1186/2050-7771-2-3 PMC3917523

[74] J. Ma , N. Li , M. Guarnera , et al., “Quantification of Plasma miRNAs by Digital PCR for Cancer Diagnosis,” Biomarker Insights 8 (2013): 127–136. 24277982 10.4137/BMI.S13154 PMC3836484

[75] M. Chen and H. Zhao , “Next‐generation Sequencing in Liquid Biopsy: Cancer Screening and Early Detection,” Human Genomics 13, no. 1 (2019): 34. 31370908 10.1186/s40246-019-0220-8 PMC6669976

[76] R. A. Joy , S. K. Thelakkattusserry , N. Vikkath , et al., “Somatic Mutation Detection Efficiency in EGFR: A Comparison Between High Resolution Melting Analysis and Sanger Sequencing,” BMC Cancer 20, no. 1 (2020): 902. 32962681 10.1186/s12885-020-07411-1 PMC7510081

[77] M. Hussain , N. Mackrides , S. Su , et al., “A Case Report of Concurrent Epidermal Growth Factor Receptor (EGFR) Exon 18 (G719A) and Exon 21 (L833_V834delinsFL) Mutations and Treatment Challenges,” Cureus 16, no. 10 (2024): e70896. 39497876 10.7759/cureus.70896 PMC11534275

[78] Chinese Society of Clinical Oncology (CSCO) Non‐small Cell Lung Cancer Committee, & Anti‐cancer Drug Safety Management Committee (ASMC) . Consensus on Application of Third‐generation EGFR‐TKI in EGFR Mutated NSCLC (2022 Version). Zhongguo Fei Ai Za Zhi 2022;25(9):627–641. 36172727 10.3779/j.issn.1009-3419.2022.101.47 PMC9549424

[79] S. Y. Sun , “Taking Early Preventive Interventions to Manage the Challenging Issue of Acquired Resistance to Third‐generation EGFR Inhibitors,” Chin Med J Pulm Crit Care Med 1, no. 1 (2023): 3–10. 37609474 10.1016/j.pccm.2022.10.001 PMC10442612

[80] Y. Li , J. Luo , W. Yip , et al., “P3.02‐062 An EGFR Follow‐On Companion Diagnostic for Clinical Care of Patients With NSCLC,” Journal of Thoracic Oncology 12, no. 11 (2017): S2260.

[81] T. Jatkoe , S. Wang , J. I. Odegaard , et al., “Clinical Validation of Companion Diagnostics for the Selection of Patients With Non‐Small Cell Lung Cancer Tumors Harboring Epidermal Growth Factor Receptor Exon 20 Insertion Mutations for Treatment With Amivantamab,” The Journal of Molecular Diagnostics: JMD 24, no. 11 (2022): 1181–1188. 35963523 10.1016/j.jmoldx.2022.07.003

[82] A. Inoue , K. Kobayashi , M. Maemondo , et al., “Updated Overall Survival Results From a Randomized Phase III Trial Comparing Gefitinib With Carboplatin‐paclitaxel for Chemo‐naïve Non‐small Cell Lung Cancer With Sensitive EGFR Gene Mutations (NEJ002),” Annals of Oncology: Official Journal of the European Society for Medical Oncology 24, no. 1 (2013): 54–59. 22967997 10.1093/annonc/mds214

[83] G. Zhang , B. Yan , Y. Guo , et al., “Case Report: A Patient With the Rare Third‐generation TKI‐resistant Mutation EGFR L718Q Who Responded to Afatinib Plus Cetuximab Combination Therapy,” Frontiers in Oncology 12 (2022): 995624. 36387265 10.3389/fonc.2022.995624 PMC9659857

[84] Z. Liang , Y. Cheng , Y. Chen , et al., “EGFR T790M ctDNA Testing Platforms and Their Role as Companion Diagnostics: Correlation With Clinical Outcomes to EGFR‐TKIs,” Cancer Letters 403 (2017): 186–194. 28642172 10.1016/j.canlet.2017.06.008

[85] C. Zhou , Y. L. Wu , G. Chen , et al., “Erlotinib versus Chemotherapy as First‐line Treatment for Patients With Advanced EGFR Mutation‐positive Non‐small‐cell Lung Cancer (OPTIMAL, CTONG‐0802): A Multicentre, Open‐label, Randomised, Phase 3 Study,” The Lancet Oncology 12, no. 8 (2011): 735–742. 21783417 10.1016/S1470-2045(11)70184-X

[86] Y. L. Wu , C. Zhou , C. P. Hu , et al., “Afatinib ersus Cisplatin Plus Gemcitabine for First‐line Treatment of Asian Patients With Advanced Non‐small‐cell Lung Cancer Harbouring EGFR Mutations (LUX‐Lung 6): An Open‐label, Randomised Phase 3 Trial,” The Lancet Oncology 15, no. 2 (2014): 213–222. 24439929 10.1016/S1470-2045(13)70604-1

[87] J. C. Soria , Y. Ohe , J. Vansteenkiste , et al., “Osimertinib in Untreated EGFR‐Mutated Advanced Non‐Small‐Cell Lung Cancer,” New England Journal of Medicine 378, no. 2 (2018): 113–125. 29151359 10.1056/NEJMoa1713137

[88] S. Hagan , M. C. Orr , and B. Doyle , “Targeted Therapies in Colorectal Cancer‐an Integrative View by PPPM,” Epma J 4, no. 1 (2013): 3. 23356214 10.1186/1878-5085-4-3 PMC3584939

[89] K. Y. Chung , J. Shia , N. E. Kemeny , et al., “Cetuximab Shows Activity in Colorectal Cancer Patients With Tumors That Do Not Express the Epidermal Growth Factor Receptor by Immunohistochemistry,” Journal of Clinical Oncology 23, no. 9 (2005): 1803–1810. 15677699 10.1200/JCO.2005.08.037

[90] N. Karachaliou and R. Rosell , “Targeted Treatment of Mutated EGFR‐expressing Non‐small‐cell Lung Cancer: Focus on Erlotinib With Companion Diagnostics,” Lung Cancer (Auckl) 5 (2014): 73–79. 28210145 10.2147/LCTT.S50671 PMC5217512

[91] S. Benlloch , M. L. Botero , J. Beltran‐Alamillo , et al., “Clinical Validation of a PCR Assay for the Detection of EGFR Mutations in Non‐small‐cell Lung Cancer: Retrospective Testing of Specimens From the EURTAC Ttrial,” PLoS ONE 9, no. 2 (2014): e89518. 24586842 10.1371/journal.pone.0089518 PMC3934888

[92] G. Goss , C. M. Tsai , F. A. Shepherd , et al., “Osimertinib for Pretreated EGFR Thr790Met‐positive Advanced Non‐small‐cell Lung Cancer (AURA2): A Multicentre, Open‐label, Single‐arm, Phase 2 Study,” The Lancet Oncology 17, no. 12 (2016): 1643–1652. 27751847 10.1016/S1470-2045(16)30508-3

[93] K. Uchibori , N. Takano , R. Manabe , et al., “Clinical Influence of Switching Companion Diagnostic Tests for EGFR‐TKs From Therascreen to Cobas V2,” Thoracic Cancer 12, no. 6 (2021): 906–913. 33528892 10.1111/1759-7714.13797 PMC7952781

[94] C. J. Robbins , K. M. Bates , and D. L. Rimm , “HER2 Testing: Evolution and Update for a Companion Diagnostic Assay,” Nature Reviews Clinical Oncology 22, no. 6 (2025): 408–423. 10.1038/s41571-025-01016-y PMC12903097 40195456

[95] J. T. Jørgensen , H. Winther , J. Askaa , et al., “A Companion Diagnostic with Significant Clinical Impact in Treatment of Breast and Gastric Cancer,” Frontiers in Oncology 11 (2021): 676939. 34367962 10.3389/fonc.2021.676939 PMC8343532

[96] U. F. Vogel , “Confirmation of a Low HER2 Positivity Rate of Breast Carcinomas—Limitations of Immunohistochemistry and in Situ Hybridization,” Diagn Pathol 5 (2010): 50. 20670419 10.1186/1746-1596-5-50 PMC2923103

[97] D. Cameron , M. J. Piccart‐Gebhart , R. D. Gelber , et al., “11 Years' Follow‐up of Trastuzumab After Adjuvant Chemotherapy in HER2‐positive Early Breast Cancer: Final Analysis of the HERceptin Adjuvant (HERA) Trial,” Lancet 389, no. 10075 (2017): 1195–1205. 28215665 10.1016/S0140-6736(16)32616-2 PMC5465633

[98] C. Keckeisen , A. Šujanová , T. Himmel , et al., “Isospora and Lankesterella Parasites (Eimeriidae, Apicomplexa) of Passeriform Birds in Europe: Infection Rates, Phylogeny, and Pathogenicity,” Pathogens 13, no. 4 (2024): 337. 38668292 10.3390/pathogens13040337 PMC11053544

[99] L. R. Grazziotin , B. R. Dada , C. de la Rosa Jaimes , et al., “Chromogenic and Silver in Situ Hybridization for Identification of HER2 Overexpression in Breast Cancer Patients: A Systematic Review and Meta‐Analysis,” Applied Immunohistochemistry & Molecular Morphology: AIMM 28, no. 6 (2020): 411–421. 31135445 10.1097/PAI.0000000000000773

[100] A. Cayre , F. Mishellany , N. Lagarde , et al., “Comparison of Different Commercial Kits for HER2 Testing in Breast Cancer: Looking for the Accurate Cutoff for Amplification,” Breast Cancer Research 9, no. 5 (2007): R64. 17908324 10.1186/bcr1770 PMC2242659

[101] A. Dilawari , H. Zhang , M. Shah , et al., “US Food and Drug Administration Approval Summary: Trastuzumab Deruxtecan for the Treatment of Adult Patients with Hormone Receptor‐Positive, Unresectable or Metastatic Human Epidermal Growth Factor Receptor 2‐Low or Human Epidermal Growth Factor Receptor 2‐Ultralow Breast Cancer,” Journal of Clinical Oncology 43, no. 26 (2025): 2942–2951. 40763319 10.1200/JCO-25-00812

[102] J. Kim , H. S. Kim , M. Nam , et al., “Tissue‐Agnostic Biomarkers in Solid Tumors: Current Approvals and Emerging Candidates,” Cancer and Metastasis Reviews 44, no. 3 (2025): 58. 40576713 10.1007/s10555-025-10274-2 PMC12204918

[103] F. Meric‐Bernstam , V. Makker , A. Oaknin , et al., “Efficacy and Safety of Trastuzumab Deruxtecan in Patients with HER2‐Expressing Solid Tumors: Primary Results from the DESTINY‐PanTumor02 Phase II Trial,” Journal of Clinical Oncology 42, no. 1 (2024): 47–58. 37870536 10.1200/JCO.23.02005 PMC10730032

[104] C. Yoo and Y. S. Park , “Companion Diagnostics for the Targeted Therapy of Gastric Cancer,” World Journal of Gastroenterology 21, no. 39 (2015): 10948–10955. 26494953 10.3748/wjg.v21.i39.10948 PMC4607896

[105] J. J. Harding , J. Fan , D. Y. Oh , et al., “Zanidatamab for HER2‐amplified, Unresectable, Locally Advanced or Metastatic Biliary Tract Cancer (HERIZON‐BTC‐01): A Multicentre, Single‐arm, Phase 2b Study,” The Lancet Oncology 24, no. 7 (2023): 772–782. 37276871 10.1016/S1470-2045(23)00242-5

[106] M. Yi , X. Zheng , M. Niu , et al., “Combination Strategies With PD‐1/PD‐L1 Blockade: Current Advances and Future Directions,” Molecular Cancer 21, no. 1 (2022): 28. 35062949 10.1186/s12943-021-01489-2 PMC8780712

[107] A. A. Davis and V. G. Patel , “The Role of PD‐L1 Expression as a Predictive Biomarker: An Analysis of all US Food and Drug Administration (FDA) Approvals of Immune Checkpoint Inhibitors,” Journal for Immunotherapy of Cancer 7, no. 1 (2019): 278. 31655605 10.1186/s40425-019-0768-9 PMC6815032

[108] D. B. Doroshow , S. Bhalla , M. B. Beasley , et al., “PD‐L1 as a Biomarker of Response to Immune‐checkpoint Inhibitors,” Nature Reviews Clinical Oncology 18, no. 6 (2021): 345–362. 10.1038/s41571-021-00473-5 33580222

[109] D. Kazandjian , D. L. Suzman , G. Blumenthal , et al., “FDA Approval Summary: Nivolumab for the Treatment of Metastatic Non‐Small Cell Lung Cancer with Progression on or after Platinum‐Based Chemotherapy,” The Oncologist 21, no. 5 (2016): 634–642. 26984449 10.1634/theoncologist.2015-0507 PMC4861371

[110] J. Sul , G. M. Blumenthal , X. Jiang , et al., “FDA Approval Summary: Pembrolizumab for the Treatment of Patients with Metastatic Non‐Small Cell Lung Cancer whose Tumors Express Programmed Death‐Ligand 1,” The Oncologist 21, no. 5 (2016): 643–650. 27026676 10.1634/theoncologist.2015-0498 PMC4861368

[111] M. Skrzypski and J. Jassem , “Consolidation Systemic Treatment After Radiochemotherapy for Unresectable Stage III Non‐small Cell Lung Cancer,” Cancer Treatment Reviews 66 (2018): 114–121. 29738940 10.1016/j.ctrv.2018.04.001

[112] F. Ohyanagi , M. Nomura , J. Shiihara , et al., “P37.08 OncomineTM Dx Target Test Companion Diagnostic System for Advanced Non‐Small Cell Lung Cancer,” Journal of Thoracic Oncology 16, no. 3 (2021): S445.

[113] S. Marwitz , S. Scheufele , S. Perner , et al., “Epigenetic Modifications of the Immune‐checkpoint Genes CTLA4 and PDCD1 in Non‐small Cell Lung Cancer Results in Increased Expression,” Clinical Epigenetics 9 (2017): 51. 28503213 10.1186/s13148-017-0354-2 PMC5426039

[114] M. Hersom and J. T. Jørgensen , “Companion and Complementary Diagnostics‐Focus on PD‐L1 Expression Assays for PD‐1/PD‐L1 Checkpoint Inhibitors in Non‐Small Cell Lung Cancer,” Therapeutic Drug Monitoring 40, no. 1 (2018): 9–16. 29084031 10.1097/FTD.0000000000000460

[115] J. T. Jørgensen , “Companion Diagnostic Assays for PD‐1/PD‐L1 Checkpoint Inhibitors in NSCLC,” Expert Review of Molecular Diagnostics 16, no. 2 (2016): 131–133. 26559787 10.1586/14737159.2016.1117389

[116] M. Rebelatto , A. Mistry , C. Sabalos , et al., “Development of a PD‐L1 Companion Diagnostic Assay for Treatment With MEDI4736 in NSCLC and SCCHN Patients,” Journal of Clinical Oncology 33, no. 15 (2015): 8033–8033.

[117] A. Dimou , K. Harrington , and K. N. Syrigos , “From the Bench to Bedside: Biological and Methodology Considerations for the Future of Companion Diagnostics in Nonsmall Cell Lung Cancer,” Pathology Research International 2011 (2011): 312346. 21785682 10.4061/2011/312346 PMC3140218

[118] M. Moutafi and D. L. Rimm , “Putting the Microenvironment Into the Immunotherapy Companion Diagnostic,” Clinical Cancer Research: An Official Journal of the American Association for Cancer Research 27, no. 14 (2021): 3812–3814. 33986024 10.1158/1078-0432.CCR-21-1238

[119] J. Caldara , J. S. Krueger , E. Ergon , et al., “Abstract 4923: Analysis of Companion Diagnostic Potentials for Multifaceted PD‐L1 Assays,” Cancer Research 79, no. 13 (2019): 4923–4923.31331910

[120] E. Chang , L. Pelosof , S. Lemery , et al., “Systematic Review of PD‐1/PD‐L1 Inhibitors in Oncology: From Personalized Medicine to Public Health,” The Oncologist 26, no. 10 (2021): e1786–e1799. 34196068 10.1002/onco.13887 PMC8488782

[121] M. Dolled‐Filhart , C. Roach , G. Toland , et al., “Development of a Companion Diagnostic for Pembrolizumab in Non‐Small Cell Lung Cancer Using Immunohistochemistry for Programmed Death Ligand‐1,” Archives of Pathology & Laboratory Medicine 140, no. 11 (2016): 1243–1249. 27552095 10.5858/arpa.2015-0542-OA

[122] W. T. Huang , T. Yun , C. H. Chew , et al., “Microtube Array Membrane Hollow Fiber Assay (MTAM‐HFA)‐An Accurate and Rapid Potential Companion Diagnostic and Pharmacological Interrogation Solution for Cancer Immunotherapy (PD‐1/PD‐L1),” Biomolecules 12, no. 4 (2022): 480. 35454072 10.3390/biom12040480 PMC9027612

[123] A. R. Hansen and L. L. Siu , “PD‐L1 Testing in Cancer: Challenges in Companion Diagnostic Development,” JAMA Oncology 2, no. 1 (2016): 15–16. 26562503 10.1001/jamaoncol.2015.4685

[124] D. L. Rimm , G. Han , J. M. Taube , et al., “Reanalysis of the NCCN PD‐L1 Companion Diagnostic Assay Study for Lung Cancer in the Context of PD‐L1 Expression Findings in Triple‐negative Breast Cancer,” Breast Cancer Research 21, no. 1 (2019): 72. 31196152 10.1186/s13058-019-1156-6 PMC6567382

[125] K. Mizutani , K. Horie , T. Kato , et al., “Serum PD‐1 Levels Measured by ELISA Using Nivolumab Increased in Advanced RCC Patients: Novel Approach to Develop Companion Diagnostics for Antibody Therapy,” Journal of Cancer Research and Clinical Oncology 145, no. 6 (2019): 1661–1663. 30515569 10.1007/s00432-018-2806-2 PMC6527537

[126] D. König , S. Savic Prince , and S. I. Rothschild , “Targeted Therapy in Advanced and Metastatic Non‐Small Cell Lung Cancer. An Update on Treatment of the Most Important Actionable Oncogenic Driver Alterations,” Cancers (Basel) 13, no. 4 (2021): 804. 33671873 10.3390/cancers13040804 PMC7918961

[127] R. C. Maloney , M. Zhang , H. Jang , et al., “The Mechanism of Activation of Monomeric B‐RAF V600E,” Computational and Structural Biotechnology Journal 19 (2021): 3349–3363. 34188782 10.1016/j.csbj.2021.06.007 PMC8215184

[128] R. Langland , T. Sharp , J. Tsai , et al., “Development of a Companion Diagnostic Test for Inhibitors of V600E BRAF,” Clinical Cancer Research 12, no. 19_Supplement (2014): A13–A13.

[129] H. Halait , K. Demartin , S. Shah , et al., “Analytical Performance of a Real‐time PCR‐based Assay for V600 Mutations in the BRAF Gene, Used as the Companion Diagnostic Test for the Novel BRAF Inhibitor Vemurafenib in Metastatic Melanoma,” Diagnostic Molecular Pathology: The American Journal of Surgical Pathology, Part B 21, no. 1 (2012): 1–8. 22306669 10.1097/PDM.0b013e31823b216f

[130] F. Lopez‐Rios , B. Angulo , B. Gomez , et al., “Comparison of Testing Methods for the Detection of BRAF V600E Mutations in Malignant Melanoma: Pre‐approval Validation Study of the Companion Diagnostic Test for Vemurafenib,” PLoS ONE 8, no. 1 (2013): e53733. 23326492 10.1371/journal.pone.0053733 PMC3542342

[131] L. Huang , C. Wen , X. Yang , et al., “PEAK1, Acting as a Tumor Promoter in Colorectal Cancer, Is Regulated by the EGFR/KRas Signaling Axis and miR‐181d,” Cell Death & Disease 9, no. 3 (2018): 271. 29449544 10.1038/s41419-018-0320-8 PMC5833579

[132] J. M. Bauml , B. T. Li , V. Velcheti , et al., “Clinical Validation of Guardant360 CDx as a Blood‐based Companion Diagnostic for Sotorasib,” Lung Cancer (Amsterdam, Netherlands) 166 (2022): 270–278. 34838325 10.1016/j.lungcan.2021.10.007 PMC10325630

[133] O. O'Brien , M. C. Wright , and C. O'Brien , “Cost‐Efficient and Easy to Perform PCR‐Based Assay to Identify Met Exon 14 Skipping in Formalin‐Fixed Paraffin‐Embedded (FFPE) Non‐Small Cell Lung Cancer (NSCLC) Samples,” Diagnostics (Basel) 9, no. 1 (2019): 13. 30669306 10.3390/diagnostics9010013 PMC6468531

[134] G. R. Oxnard , J. C. Yang , H. Yu , et al., “TATTON: A Multi‐arm, Phase Ib Trial of Osimertinib Combined With Selumetinib, Savolitinib, or Durvalumab in EGFR‐mutant Lung Cancer,” Annals of Oncology: Official Journal of the European Society for Medical Oncology 31, no. 4 (2020): 507–516. 32139298 10.1016/j.annonc.2020.01.013

[135] M. Schuler , R. Berardi , W. T. Lim , et al., “Molecular Correlates of Response to Capmatinib in Advanced Non‐small‐cell Lung Cancer: Clinical and Biomarker Results From a Phase I Trial,” Annals of Oncology: Official Journal of the European Society for Medical Oncology 31, no. 6 (2020): 789–797. 32240796 10.1016/j.annonc.2020.03.293 PMC9720758

[136] S. M. Wu , W. S. Tsai , S. F. Chiang , et al., “Comprehensive Transcriptome Profiling of Taiwanese Colorectal Cancer Implicates an Ethnic Basis for Pathogenesis,” Scientific Reports 10, no. 1 (2020): 4526. 32161294 10.1038/s41598-020-61273-y PMC7066141

[137] S. Kang , J. Woo , and S. Kim , “A Systematic Review of Companion Diagnostic Tests by Immunohistochemistry for the Screening of Alectinib‐Treated Patients in ALK‐Positive Non‐Small Cell Lung Cancer,” Diagnostics (Basel) 12, no. 5 (2022): 1297. 35626451 10.3390/diagnostics12051297 PMC9140374

[138] K. Takeuchi , Y. Togashi , Y. Kamihara , et al., “Prospective and Clinical Validation of ALK Immunohistochemistry: Results From the Phase I/II Study of Alectinib for ALK‐positive Lung Cancer (AF‐001JP study),” Annals of Oncology: Official Journal of the European Society for Medical Oncology 27, no. 1 (2016): 185–192. 26487585 10.1093/annonc/mdv501 PMC4684157

[139] L. Nguyen , W. M. Martens J , A. Van Hoeck , et al., “Pan‐cancer Landscape of Homologous Recombination Deficiency,” Nature Communications 11, no. 1 (2020): 5584. 10.1038/s41467-020-19406-4 PMC7643118 33149131

[140] C. J. LaFargue , G. Z. Dal Molin , A. K. Sood , et al., “Exploring and Comparing Adverse Events Between PARP Inhibitors,” The Lancet Oncology 20, no. 1 (2019): e15–e28. 30614472 10.1016/S1470-2045(18)30786-1 PMC7292736

[141] L. E. Dockery , C. C. Gunderson , and K. N. Moore , “Rucaparib: The Past, Present, and Future of a Newly Approved PARP Inhibitor for Ovarian Cancer,” OncoTargets and Therapy 10 (2017): 3029–3037. 28790837 10.2147/OTT.S114714 PMC5488752

[142] C. C. Gunderson and K. N. Moore , “BRACAnalysis CDx as a Companion Diagnostic Tool for Lynparza,” Expert Review of Molecular Diagnostics 15, no. 9 (2015): 1111–1116. 26292709 10.1586/14737159.2015.1078238

[143] S. Dunn , “Dr Sandi Dunn Illuminates Emerging Key Treatment Approaches and Companion Diagnostics for Triple Negative Breast Cancer,” Expert Opinion on Investigational Drugs 29, no. 12 (2020): 1309–1312. 33070643 10.1080/13543784.2020.1839847

[144] T. Golan , P. Hammel , M. Reni , et al., “Maintenance Olaparib for Germline BRCA‐Mutated Metastatic Pancreatic Cancer,” New England Journal of Medicine 381, no. 4 (2019): 317–327. 31157963 10.1056/NEJMoa1903387 PMC6810605

[145] J. de Bono , J. Mateo , and K. Fizazi , “Olaparib for Metastatic Castration‐Resistant Prostate Cancer,” New England Journal of Medicine 382, no. 22 (2020): 2091–2102. 32343890 10.1056/NEJMoa1911440

[146] V. Kumar , J. Yu , V. Phan , et al., “Androgen Receptor Immunohistochemistry as a Companion Diagnostic Approach to Predict Clinical Response to Enzalutamide in Triple‐Negative Breast Cancer,” JCO Precision Oncology 1 (2017): 1–19. 10.1200/PO.17.00075 35172518

[147] Y. Kimura , T. Itagaki , and M. Ohara , “Companion Diagnosis by BRACAnalysis CDx^TM^ and Therapeutic Experience of Olaparib for HER2‐negative Breast Cancer,” Annals of Oncology 30 (2019): vi136.

[148] J. S. Kwon , A. V. Tinker , A. Karsan , et al., “Costs and Benefits of Tumor Testing for Mutations in High‐grade Serous Ovarian Cancer as a Companion Diagnostic for PARP Inhibitor Treatment,” Gynecologic Oncology 154 (2019): 177–177.31056111

[149] F. Sotgia and M. P. Lisanti , “Mitochondrial mRNA Transcripts Predict Overall Survival, Tumor Recurrence and Progression in Serous Ovarian Cancer: Companion Diagnostics for Cancer Therapy,” Oncotarget 8, no. 40 (2017): 66925–66939. 28978006 10.18632/oncotarget.19963 PMC5620146

[150] K. L. Copeland , S. B. Wehnelt , L. E. Wange , et al., “Outcomes of Clinical Testing for Tumor BRAC1 and BRCA2 Gene Analysis for 354 Patients: First Experience With Tumor Companion Diagnostic for PARP Inhibitors,” Annals of Oncology 27 (2016): vi301.

[151] T. Takeda , K. Tsuji , Y. Kobayashi , et al., “Clinical and Pathological Analysis of Companion Diagnostic Testing of Microsatellite Instability‐high for Pembrolizumab in Gynaecologic Malignancy,” Japanese Journal of Clinical Oncology 52, no. 2 (2022): 128–133. 34750611 10.1093/jjco/hyab175

[152] L. Serpe , M. Gallicchio , R. Canaparo , et al., “Targeted Treatment of Folate Receptor‐positive Platinum‐resistant Ovarian Cancer and Companion Diagnostics, With Specific Focus on Vintafolide and Etarfolatide,” Pharmacogenomics and Personalized Medicine 7 (2014): 31–42. 24516337 10.2147/PGPM.S58374 PMC3917542

[153] D. Mancini‐DiNardo and K. Brown , “Precision Medicine and Companion Diagnostics Join the Battle against Ovarian Cancer,” MLO: Medical Laboratory Observer 48, no. 10 (2016): 36–37. 30047653

[154] S. Balasubramaniam , J. A. Beaver , S. Horton , et al., “FDA Approval Summary: Rucaparib for the Treatment of Patients With Deleterious BRCA Mutation‐Associated Advanced Ovarian Cancer,” Clinical Cancer Research: An Official Journal of the American Association for Cancer Research 23, no. 23 (2017): 7165–7170. 28751443 10.1158/1078-0432.CCR-17-1337

[155] J. Sus , J. Bosak , and T. Hauser , “Crushing Tablets or Sprinkling Capsules: Implications for Clinical Strategy and Study Performance Based on BE Studies of Rivaroxaban and Deferasirox,” Clinical and Translational Science 17, no. 3 (2024): e13752. 38511529 10.1111/cts.13752 PMC10955620

[156] A. Tracy , J. Kahn , B. Geng , et al., “A Novel Case of Chronic Spontaneous Urticaria Associated Chronic Myelogenous Leukemia With Rapid Resolution Upon Treatment With Imatinib,” JAAD Case Rep 30 (2022): 21–23. 36345411 10.1016/j.jdcr.2022.09.025 PMC9636012

[157] D. P. Dash and D. Dinauer , “FDA Cleared Companion Diagnostics (CDx) Tests (IDH1, IDH2 and FLT3) for Acute Myeloid Leukemia (AML) Patient Care,” Blood 138 (2021): 4442.

[158] J. M. Politei and A. Patrono , “Clinically Meaningful Outcomes After 1 Year of Treatment With Setmelanotide in an Adult Patient With a Variant in SH2B1,” Obes Facts 17, no. 6 (2024): 646–651. 39284294 10.1159/000541267 PMC11661841

[159] S. Souchelnytskyi and N. Souchelnytskyi , “Chloroquine Use in the Treatment of COVID‐19: Systems Biology Report of Common Targets of SARS‐CoV‐2 and Chloroquine,” Research Square (2020). [Preprint].

[160] M. Sandquist and H. R. Wong , “Biomarkers of Sepsis and Their Potential Value in Diagnosis, Prognosis and Treatment,” Expert Review of Clinical Immunology 10, no. 10 (2014): 1349–1356. 25142036 10.1586/1744666X.2014.949675 PMC4654927

[161] B. Wang , X. Zhang , H. Chen , et al., “A Review of Intraocular Biomolecules in Retinal Vein Occlusion: Toward Potential Biomarkers for Companion Diagnostics,” Frontiers in Pharmacology 13 (2022): 859951. 35559255 10.3389/fphar.2022.859951 PMC9086509

[162] K. Timmerman , M. Weekes , G. Traversy , et al., “Evidence for Optimal HIV Screening and Testing Intervals in HIV‐negative Individuals From Various Risk Groups: A Systematic Review,” Canada Communicable Disease Report = Releve des maladies transmissibles au Canada 44, no. 12 (2018): 337–347. 31517954 10.14745/ccdr.v44i12a05 PMC6707414

[163] M. L. Bolognesi , A. Gandini , F. Prati , et al., “From Companion Diagnostics to Theranostics: A New Avenue for Alzheimer's Disease?,” Journal of Medicinal Chemistry 59, no. 17 (2016): 7759–7770. 27124551 10.1021/acs.jmedchem.6b00151

[164] D. K. Tripathi , K. Srivastava , K. L. Nagpal , et al., “Exploration of some New Secretory Proteins to Be Employed for Companion Diagnosis of Mycobacterium Tuberculosis,” Immunology Letters 209 (2019): 67–74. 30898660 10.1016/j.imlet.2019.03.010

[165] R. B. Stricker and L. Johnson , “Lyme Disease: The Promise of Big Data, Companion Diagnostics and Precision Medicine,” Infection and Drug Resistance 9 (2016): 215–219. 27672336 10.2147/IDR.S114770 PMC5024771

[166] M. Ruhwald , L. de Thurah , D. Kuchaka , et al., “Introducing the ESAT‐6 Free IGRA, a Companion Diagnostic for TB Vaccines Based on ESAT‐6,” Scientific Reports 7 (2017): 45969. 28387329 10.1038/srep45969 PMC5384086

[167] K. Kasuno , J. Yodoi , and M. Iwano , “Urinary Thioredoxin as a Biomarker of Renal Redox Dysregulation and a Companion Diagnostic to Identify Responders to Redox‐Modulating Therapeutics,” Antioxidants & Redox Signaling 36, no. 13‐15 (2022): 1051–1065. 34541903 10.1089/ars.2021.0194

[168] H. Matsumoto , “Serum Periostin: A Novel Biomarker for Asthma Management,” Allergology International: Official Journal of the Japanese Society of Allergology 63, no. 2 (2014): 153–160. 24759559 10.2332/allergolint.13-RAI-0678

[169] R. He , P. Qi , L. Shu , et al., “Dysbiosis and Extraintestinal Cancers,” Journal of Experimental & Clinical Cancer Research 44, no. 1 (2025): 44. 39915884 10.1186/s13046-025-03313-x PMC11804008

[170] D. Krupka , K. Rakoczy , A. Chełmoński , et al., “Crucial Role of Early Detection in Managing Heart Failure in Kearns‐Sayre Syndrome: A Case Report,” Am J Case Rep 26 (2025): e947439. 40824877 10.12659/AJCR.947439 PMC12372848

[171] J. Zhong , Y. Liu , N. Luo , et al., “Metagenomic Next‐generation Sequencing for Rapid Detection of Pulmonary Infection in Patients With Acquired Immunodeficiency Syndrome,” Ann Clin Microbiol Antimicrob 22, no. 1 (2023): 57. 37430367 10.1186/s12941-023-00608-9 PMC10334547

[172] S. R. Vitale , F. H. Groenendijk , R. van Marion , et al., “TP53 Mutations in Serum Circulating Cell‐Free Tumor DNA as Longitudinal Biomarker for High‐Grade Serous Ovarian Cancer,” Biomolecules 10, no. 3 (2020): 415. 32156073 10.3390/biom10030415 PMC7175353

[173] S. Bernardi , A. Cavalleri , S. Mutti , et al., “Digital PCR (dPCR) Is Able to Anticipate the Achievement of Stable Deep Molecular Response in Adult Chronic Myeloid Leukemia Patients: Results of the DEMONSTRATE Study,” Annal of Hematology 104, no. 1 (2025): 207–217. 10.1007/s00277-024-06100-4 PMC11868186 39611878

[174] M. Fontanilles , F. Marguet , P. Ruminy , et al., “Simultaneous Detection of EGFR Amplification and EGFRvIII Variant Using Digital PCR‐based Method in Glioblastoma,” Acta Neuropathol Commun 8, no. 1 (2020): 52. 32303258 10.1186/s40478-020-00917-6 PMC7165387

[175] L. F. Leite da Silva , E. F. Saldanha , and J. S. A. de Menezes , “Plasma ctDNA Kinetics as a Predictor of Systemic Therapy Response for Advanced Non‐small Cell Lung Cancer: A Systematic Review and Meta‐analysis,” The Oncologist 30, no. 2 (2025): oyae344. 39998904 10.1093/oncolo/oyae344 PMC11853598

[176] E. Lin , J. Chien , F. S. Ong , et al., “Challenges and Opportunities for Next‐generation Sequencing in Companion Diagnostics,” Expert Review of Molecular Diagnostics 15, no. 2 (2015): 193–209. 25249308 10.1586/14737159.2015.961916

[177] J. D. Khoury and D. V. Catenacci , “Next‐generation Companion Diagnostics: Promises, Challenges, and Solutions,” Archives of Pathology & Laboratory Medicine 139, no. 1 (2015): 11–13. 25166874 10.5858/arpa.2014-0063-ED PMC4991626

[178] G. J. Doherty , M. Petruzzelli , E. Beddowes , et al., “Cancer Treatment in the Genomic Era,” Annual Review of Biochemistry 88 (2019): 247–280. 10.1146/annurev-biochem-062917-011840 30901264

[179] K. Kato , J. Okami , H. Nakamura , et al., “Analytical Performance of a Highly Sensitive System to Detect Gene Variants Using Next‐Generation Sequencing for Lung Cancer Companion Diagnostics,” Diagnostics (Basel) 13, no. 8 (2023): 1476. 37189577 10.3390/diagnostics13081476 PMC10137435

[180] V. Schacht and J. S. Kern , “Basics of Immunohistochemistry,” The Journal of Investigative Dermatology 135, no. 3 (2015): 1–4. 10.1038/jid.2014.541 25666678

[181] A. K. Attuluri , C. P. V. Serkad , A. Gunda , et al., “Analytical Validation of CanAssist‐Breast: An Immunohistochemistry Based Prognostic Test for Hormone Receptor Positive Breast Cancer Patients,” BMC Cancer 19, no. 1 (2019): 249. 30894144 10.1186/s12885-019-5443-5 PMC6425559

[182] C. Roach , N. Zhang , E. Corigliano , et al., “Development of a Companion Diagnostic PD‐L1 Immunohistochemistry Assay for Pembrolizumab Therapy in Non‐Small‐cell Lung Cancer,” Applied Immunohistochemistry & Molecular Morphology: AIMM 24, no. 6 (2016): 392–397. 27333219 10.1097/PAI.0000000000000408 PMC4957959

[183] T. Neuman , M. London , and J. Kania‐Almog , “A Harmonization Study for the Use of 22C3 PD‐L1 Immunohistochemical Staining on Ventana's Platform,” Journal of Thoracic Oncology: Official Publication of the International Association for the Study of Lung Cancer 11, no. 11 (2016): 1863–1868. 27664534 10.1016/j.jtho.2016.08.146

[184] J. D. Twomey and B. Zhang , “Cancer Immunotherapy Update: FDA‐Approved Checkpoint Inhibitors and Companion Diagnostics,” The AAPS Journal 23, no. 2 (2021): 39. 33677681 10.1208/s12248-021-00574-0 PMC7937597

[185] E. Torlakovic , H. J. Lim , J. Adam , et al., ““Interchangeability” of PD‐L1 Immunohistochemistry Assays: A Meta‐analysis of Diagnostic Accuracy,” Modern Pathology: An Official Journal of the United States and Canadian Academy of Pathology, Inc 33, no. 1 (2020): 4–17. 31383961 10.1038/s41379-019-0327-4 PMC6927905

[186] A. I. Fernandez , P. Gaule , and D. L. Rimm , “Tissue Age Affects Antigenicity and Scoring for the 22C3 Immunohistochemistry Companion Diagnostic Test,” Modern Pathology: An Official Journal of the United States and Canadian Academy of Pathology, Inc 36, no. 7 (2023): 100159. 36925070 10.1016/j.modpat.2023.100159 PMC10502188

[187] J. T. Jørgensen and M. Hersom , “Companion Diagnostics‐a Tool to Improve Pharmacotherapy,” Annals of Translational Medicine 4, no. 24 (2016): 482. 28149844 10.21037/atm.2016.12.26 PMC5233535

[188] E. Dennis , P. Banks , L. B. Murata , et al., “Validation of an Electronic Program for Pathologist Training in the Interpretation of a Complex Companion Diagnostic Immunohistochemical Assay,” Human Pathology 56 (2016): 194–203. 27349303 10.1016/j.humpath.2016.06.013

[189] J. M. Monné Rodríguez , A. L. Frisk , and R. Kreutzer , “European Society of Toxicologic Pathology (Pathology 2.0 Molecular Pathology Special Interest Group): Review of in Situ Hybridization Techniques for Drug Research and Development,” Toxicologic Pathology 51, no. 3 (2023): 92–111. 37449403 10.1177/01926233231178282 PMC10467011

[190] H. Kim and J. H. Chung , “Biomarker Testing of Cytology Specimens in Personalized Medicine for Lung Cancer Patients,” J Pathol Transl Med 56, no. 6 (2022): 326–333. 36345618 10.4132/jptm.2022.10.17 PMC9682222

[191] M. Wu , S. Yuan , K. Liu , et al., “Gastric Cancer Signaling Pathways and Therapeutic Applications,” Technology in Cancer Research & Treatment 23 (2024): 15330338241271935. 39376170 10.1177/15330338241271935 PMC11468335

[192] S. N. Lone , S. Nisar , T. Masoodi , et al., “Liquid Biopsy: A Step Closer to Transform Diagnosis, Prognosis and Future of Cancer Treatments,” Molecular Cancer 21, no. 1 (2022): 79. 35303879 10.1186/s12943-022-01543-7 PMC8932066

[193] Y. Sato , “Clinical Utility of Liquid Biopsy‐based Companion Diagnostics in the Non‐small‐cell Lung Cancer Treatment,” Exploration of Targeted Anti‐Tumor Therapy 3, no. 5 (2022): 630–642. 36338524 10.37349/etat.2022.00104 PMC9630093

[194] S. Mirza , K. Bhadresha , M. J. Mughal , et al., “Liquid Biopsy Approaches and Immunotherapy in Colorectal Cancer for Precision Medicine: Are We There Yet?,” Frontiers in Oncology 12 (2022): 1023565. 36686736 10.3389/fonc.2022.1023565 PMC9853908

[195] M. Ignatiadis , G. W. Sledge , and S. S. Jeffrey , “Liquid Biopsy Enters the Clinic—Implementation Issues and Future Challenges,” Nature Reviews Clinical Oncology 18, no. 5 (2021): 297–312. 10.1038/s41571-020-00457-x 33473219

[196] M. Harigopal , D. Kowalski , and A. Vosoughi , “Enumeration and Molecular Characterization of Circulating Tumor Cells as an Innovative Tool for Companion Diagnostics in Breast Cancer,” Expert review of Molecular Diagnostics 20, no. 8 (2020): 815–828. 32546017 10.1080/14737159.2020.1784009

[197] A. Morabito and C. Rolfo , “Small Cell Lung Cancer: A New Era Is Beginning?,” Cancers (Basel) 13, no. 11 (2021): 2646. 34071158 10.3390/cancers13112646 PMC8197965

[198] M. E. Menyailo , M. S. Tretyakova , and E. V. Denisov , “Heterogeneity of Circulating Tumor Cells in Breast Cancer: Identifying Metastatic Seeds,” International Journal of Molecular Sciences 21, no. 5 (2020): 1696. 32121639 10.3390/ijms21051696 PMC7084665

[199] K. T. Jin , X. Y. Chen , H. R. Lan , et al., “Current Progress in the Clinical Use of Circulating Tumor Cells as Prognostic Biomarkers,” Cancer Cytopathology 127, no. 12 (2019): 739–749. 31589381 10.1002/cncy.22189

[200] T. Rossi , G. Gallerani , G. Martinelli , et al., “Circulating Tumor Cells as a Tool to Untangle the Breast Cancer Heterogeneity Issue,” Biomedicines 9, no. 9 (2021): 1242. 34572427 10.3390/biomedicines9091242 PMC8466266

[201] B. Tomasik , M. Skrzypski , M. Bieńkowski , et al., “Current and Future Applications of Liquid Biopsy in Non‐small‐cell Lung Cancer‐A Narrative Review,” Translational Lung Cancer Research 12, no. 3 (2023): 594–614. 37057121 10.21037/tlcr-22-742 PMC10087994

[202] I. Casanova‐Salas , A. Athie , P. C. Boutros , et al., “Quantitative and Qualitative Analysis of Blood‐based Liquid Biopsies to Inform Clinical Decision‐making in Prostate Cancer,” European Urology 79, no. 6 (2021): 762–771. 33422353 10.1016/j.eururo.2020.12.037 PMC8941682

[203] K. Das , M. Paltani , P. K. Tripathi , et al., “Current Implications and Challenges of Artificial Intelligence Technologies in Therapeutic Intervention of Colorectal Cancer,” Exploration of Targeted Anti‐Tumor Therapy 4, no. 6 (2023): 1286–1300. 38213536 10.37349/etat.2023.00197 PMC10776591

[204] A. Stenzinger , V. Endris , J. Budczies , et al., “Harmonization and Standardization of Panel‐Based Tumor Mutational Burden Measurement: Real‐World Results and Recommendations of the Quality in Pathology Study,” Journal of Thoracic Oncology: Official Publication of the International Association for the Study of Lung Cancer 15, no. 7 (2020): 1177–1189. 32119917 10.1016/j.jtho.2020.01.023

[205] K. Shigeyasu , H. Tazawa , Y. Hashimoto , et al., “Fluorescence Virus‐guided Capturing System of Human Colorectal Circulating Tumour Cells for Non‐invasive Companion Diagnostics,” Gut 64, no. 4 (2015): 627–635. 24870621 10.1136/gutjnl-2014-306957

[206] S. Nagrath , L. V. Sequist , S. Maheswaran , et al., “Isolation of Rare Circulating Tumour Cells in Cancer Patients by Microchip Technology,” Nature 450, no. 7173 (2007): 1235–1239. 18097410 10.1038/nature06385 PMC3090667

[207] Y. Y. Huang , P. Chen , C. H. Wu , et al., “Screening and Molecular Analysis of Single Circulating Tumor Cells Using Micromagnet Array,” Scientific Reports 5 (2015): 16047. 26538094 10.1038/srep16047 PMC4633592

[208] Y. Q. Li , B. K. Chandran , C. T. Lim , et al., “Rational Design of Materials Interface for Efficient Capture of Circulating Tumor Cells,” Advanced Science (Weinheim, Baden‐Wurttemberg, Germany) 2, no. 11 (2015): 1500118. 27980914 10.1002/advs.201500118 PMC5115340

[209] D. B. Asante , L. Calapre , M. Ziman , et al., “Liquid Biopsy in Ovarian Cancer Using Circulating Tumor DNA and Cells: Ready for Prime Time?,” Cancer Letters 468 (2020): 59–71. 31610267 10.1016/j.canlet.2019.10.014

[210] V. Haselmann , C. Gebhardt , I. Brechtel , et al., “Liquid Profiling of Circulating Tumor DNA in Plasma of Melanoma Patients for Companion Diagnostics and Monitoring of BRAF Inhibitor Therapy,” Clinical Chemistry 64, no. 5 (2018): 830–842. 29483107 10.1373/clinchem.2017.281543

[211] R. Yin , Q. Zhang , S. Liao , et al., “Medical Imaging‐based Companion Diagnostics for Solid Tumors,” EngMedicine 1, no. 1 (2024): 100009.

[212] J. Schwenck , D. Sonanini , J. M. Cotton , et al., “Advances in PET Imaging of Cancer,” Nature Reviews Cancer 23, no. 7 (2023): 474–490. 37258875 10.1038/s41568-023-00576-4

[213] S. P. Rowe and M. G. Pomper , “Molecular Imaging in Oncology: Current Impact and Future Directions,” CA: A Cancer Journal for Clinicians 72, no. 4 (2022): 333–352. 34902160 10.3322/caac.21713 PMC9189244

[214] E. K. Alexander and E. S. Cibas , “Diagnosis of Thyroid Nodules,” The Lancet Diabetes & Endocrinology 10, no. 7 (2022): 533–539. 35752200 10.1016/S2213-8587(22)00101-2

[215] Y. Gao , C. Hernandez , H. X. Yuan , et al., “Ultrasound Molecular Imaging of Ovarian Cancer With CA‐125 Targeted Nanobubble Contrast Agents,” Nanomedicine: Nanotechnology, Biology, And Medicine 13, no. 7 (2017): 2159–2168. 28603079 10.1016/j.nano.2017.06.001 PMC11686501

[216] Z. Dong , P. Liang , G. Guan , et al., “Overcoming Hypoxia‐Induced Ferroptosis Resistance via a ^19^F/^1^H‐MRI Traceable Core‐Shell Nanostructure,” Angewandte Chemie (International ed in English) 61, no. 48 (2022): e202206074. 36222012 10.1002/anie.202206074

[217] L. Succony , D. M. Rassl , A. P. Barker , et al., “Adenocarcinoma Spectrum Lesions of the Lung: Detection, Pathology and Treatment Strategies,” Cancer Treatment Reviews 99 (2021): 102237. 34182217 10.1016/j.ctrv.2021.102237

[218] X. Cheng , Y. Chai , J. Xu , et al., “Enzyme Cascade Reaction‐based Ratiometric Fluorescence Probe for Visual Monitoring the Activity of Alkaline Phosphatase,” Sensors and Actuators B: Chemical 309 (2020): 127765.

[219] M. Yassin , A. T. Soliman , V. De Sanctis , et al., “A Young Adult With Unintended Acute Intravenous Iron Intoxication Treated With Oral Chelation: The Use of Liver Ferriscan for Diagnosing and Monitoring Tissue Iron Load,” Mediterr J Hematol Infect Dis 9, no. 1 (2017): e2017008. 28101313 10.4084/MJHID.2017.008 PMC5224804

[220] J. C. Wood , S. Pressel , Z. R. Rogers , et al., “Liver Iron Concentration Measurements by MRI in Chronically Transfused Children With Sickle Cell Anemia: Baseline Results From the TWiTCH Trial,” American Journal of Hematology 90, no. 9 (2015): 806–810. 26087998 10.1002/ajh.24089 PMC4546569

[221] R. Yue , Z. Li , H. Liu , et al., “Imaging‐guided Companion Diagnostics in Radiotherapy by Monitoring APE1 Activity With Afterglow and MRI Imaging,” Nature Communications 15, no. 1 (2024): 6349. 10.1038/s41467-024-50688-0 PMC11283504 39068156

[222] B. V. Marquez‐Nostra , S. Lee , R. Laforest , et al., “Preclinical PET Imaging of Glycoprotein Non‐metastatic Melanoma B in Triple Negative Breast Cancer: Feasibility of an Antibody‐based Companion Diagnostic Agent,” Oncotarget 8, no. 61 (2017): 104303–104314. 29262642 10.18632/oncotarget.22228 PMC5732808

[223] X. Pan , Q. Wu , Y. Zhang , et al., “Development and Preclinical Evaluation of CDH17‐Specific ImmunoPET Imaging in Colorectal Cancers,” Mol Pharm 22, no. 9 (2025): 5512–5522. 40785215 10.1021/acs.molpharmaceut.5c00525

[224] W. Huang , Y. Zhang , M. Cao , et al., “ImmunoPET Imaging of TROP2 in Patients With Solid Tumours,” EMBO Molecular Medicine 16, no. 5 (2024): 1143–1161. 38565806 10.1038/s44321-024-00059-5 PMC11099157

[225] W. Huang , T. Wang , Y. Qiu , et al., “CD38‐specific ImmunoPET Imaging for Multiple Myeloma Diagnosis and Therapeutic Monitoring: Preclinical and First‐in‐human Studies,” European Journal of Nuclear Medicine and Molecular Imaging 52, no. 5 (2025): 1791–1804. 39725695 10.1007/s00259-024-07036-7

[226] N. C. Wong , Y. Cai , L. K. Meszaros , et al., “Preclinical Development and Characterisation of ^99^Tc‐NM‐01 for SPECT/CT Imaging of human PD‐L1,” Am J Nucl Med Mol Imaging 11, no. 3 (2021): 154–166. 34234994 PMC8255215

[227] R. A. Rissman , M. C. Donohue , O. Langford , et al., “Longitudinal Phospho‐tau217 Predicts Amyloid Positron Emission Tomography in Asymptomatic Alzheimer's Disease,” J Prev Alzheimers Dis 11, no. 4 (2024): 823–830. 39044490 10.14283/jpad.2024.134 PMC11266279

[228] S. Stacchiotti , S. Martini , S. Pasquali , et al., “GDF‐15 Predicts Epithelioid Hemangioendothelioma Aggressiveness and Is Downregulated by Sirolimus Through ATF4/ATF5 Suppression,” Clinical Cancer Research: An Official Journal of the American Association for Cancer Research 30, no. 22 (2024): 5122–5137. 39283723 10.1158/1078-0432.CCR-23-3991 PMC11565171

[229] J. Sebastiano , S. A. McGlone , Z. V. Samuels , et al., “Evaluating Radiotheranostic Targets for Endometrial Cancer,” Journal of Nuclear Medicine 66, no. 10 (2025): 1631–1638. 40841152 10.2967/jnumed.125.270318 PMC12487871

[230] M. Zhou , B. Chen , C. Lu , et al., “ImmunoPET Imaging of LAG‐3 Expression in Tumor Microenvironment With ^68^Ga‐labelled Cyclic Peptides Tracers: From Bench to Bedside,” Journal for Immunotherapy of Cancer 12, no. 7 (2024): e009153. 39060024 10.1136/jitc-2024-009153 PMC11284836

[231] Y. Meng , J. Sun , N. Qv , et al., “Application of Molecular Imaging Technology in Tumor Immunotherapy,” Cellular Immunology 348 (2020): 104039. 32007223 10.1016/j.cellimm.2020.104039

[232] K. M. Tully , N. B. Sobol , P. M. R. Pereira , et al. Chapter 10 ‐ Molecular Imaging Companion Diagnostics. in Companion and Complementary Diagnostics, ed. J. T. Jørgensen (Academic Press, 2019): 201–228.

[233] D. Locke and C. C. Hoyt , “Companion Diagnostic Requirements for Spatial Biology Using Multiplex Immunofluorescence and Multispectral Imaging,” Frontiers in Molecular Biosciences 10 (2023): 1051491. 36845550 10.3389/fmolb.2023.1051491 PMC9948403

[234] R. L. Van Heertum , R. Scarimbolo , R. Ford , et al., “Companion Diagnostics and Molecular Imaging‐enhanced Approaches for Oncology Clinical Trials,” Drug Design, Development and Therapy 9 (2015): 5215–5223. 26392755 10.2147/DDDT.S87561 PMC4573073

[235] D. S. Lee and G. J. Cheon , “Nuclear Theranostics in Asia: In Vivo Companion Diagnostics,” Nuclear Medicine and Molecular Imaging 53, no. 1 (2019): 1–6. 30828392 10.1007/s13139-019-00573-2 PMC6377583

[236] K. Mishiro , R. Nishii , I. Sawazaki , et al., “Development of Radiohalogenated Osimertinib Derivatives as Imaging Probes for Companion Diagnostics of Osimertinib,” Journal of Medicinal Chemistry 65, no. 3 (2022): 1835–1847. 35015529 10.1021/acs.jmedchem.1c01211

[237] Y. Zeng , T. Dou , L. Ma , et al., “Biomedical Photoacoustic Imaging for Molecular Detection and Disease Diagnosis: , “Always‐On” and , “Turn‐On” Probes,” Advanced Science (Weinheim, Baden‐Wurttemberg, Germany) 9, no. 25 (2022): e2202384. 35773244 10.1002/advs.202202384 PMC9443455

[238] M. Y. Lucero and J. Chan , “Photoacoustic Imaging of Elevated Glutathione in Models of Lung Cancer for Companion Diagnostic Applications,” Nature Chemistry 13, no. 12 (2021): 1248–1256. 10.1038/s41557-021-00804-0 PMC8629919 34697400

[239] J. Wischhusen , K. E. Wilson , J. G. Delcros , et al., “Ultrasound Molecular Imaging as a Non‐invasive Companion Diagnostic for Netrin‐1 Interference Therapy in Breast Cancer,” Theranostics 8, no. 18 (2018): 5126–5142. 30429890 10.7150/thno.27221 PMC6217066

[240] A. W. Woodham , S. H. Zeigler , E. L. Zeyang , et al., “In Vivo Detection of Antigen‐specific CD8^+^ T Cells by Immuno‐positron Emission Tomography,” Nature Methods 17, no. 10 (2020): 1025–1032. 32929269 10.1038/s41592-020-0934-5 PMC7541633

[241] B. N. McKnight and N. T. Viola‐Villegas , “ ^89^Zr‐ImmunoPET Companion Diagnostics and Their Impact in Clinical Drug Development,” Journal of Labelled Compounds & Radiopharmaceuticals 61, no. 9 (2018): 727–738. 29341222 10.1002/jlcr.3605 PMC6050145

[242] K. S. Carmon and A. Azhdarinia , “Application of Immuno‐PET in Antibody‐Drug Conjugate Development,” Molecular Imaging 17 (2018): 1536012118801223. 30370812 10.1177/1536012118801223 PMC6207972

[243] O. Ilovich , A. Natarajan , S. Hori , et al., “Development and Validation of an Immuno‐PET Tracer as a Companion Diagnostic Agent for Antibody‐Drug Conjugate Therapy to Target the CA6 Epitope,” Radiology 276, no. 1 (2015): 191–198. 25734548 10.1148/radiol.15140058 PMC4570559

[244] M. Loft , C. Christensen , M. M. Clausen , et al., “First‐in‐Humans PET Imaging of Tissue Factor in Patients With Primary and Metastatic Cancers Using ^18^F‐labeled Active‐Site Inhibited Factor VII (^18^F‐ASIS): Potential as Companion Diagnostic,” Journal of Nuclear Medicine 63, no. 12 (2022): 1871–1879. 35589407 10.2967/jnumed.122.264068 PMC9730914

[245] V. A. Naumenko , S. S. Vodopyanov , K. Y. Vlasova , et al., “Intravital Imaging of Liposome Behavior Upon Repeated Administration: A Step towards the Development of Liposomal Companion Diagnostic for Cancer Nanotherapy,” Journal of Controlled Release: Official Journal of the Controlled Release Society 330 (2021): 244–256. 33333122 10.1016/j.jconrel.2020.12.014

[246] A. D. Puranik , H. R. Kulkarni , and R. P. Baum , “Companion Diagnostics and Molecular Imaging,” Cancer Journal (Sudbury, Mass) 21, no. 3 (2015): 213–217. 26049701 10.1097/PPO.0000000000000110

[247] J. M. Idée , S. Louguet , S. Ballet , et al., “Theranostics and Contrast‐agents for Medical Imaging: A Pharmaceutical Company Viewpoint,” Quantitative Imaging in Medicine and Surgery 3, no. 6 (2013): 292–297. 24404442 10.3978/j.issn.2223-4292.2013.12.06 PMC3882809

[248] D. Gao , X. Guo , Y. Yang , et al., “Microfluidic Chip and Isothermal Amplification Technologies for the Detection of Pathogenic Nucleic Acid,” Journal of Biological Engineering 16, no. 1 (2022): 33. 36457138 10.1186/s13036-022-00312-w PMC9714395

[249] T. H. M. Moore , S. Dawson , K. Kirby , et al., “Point‐of‐care Tests in the Emergency Medical Services: A Scoping Review,” Scandinavian Journal of Trauma, Resuscitation and Emergency Medicine 33, no. 1 (2025): 18. 39901298 10.1186/s13049-025-01329-y PMC11792643

[250] U. P. Rohr , C. Binder , T. Dieterle , et al., “The Value of in Vitro Diagnostic Testing in Medical Practice: A Status Report,” PLoS ONE 11, no. 3 (2016): e0149856. 26942417 10.1371/journal.pone.0149856 PMC4778800

[251] S. B. Haga , “Challenges of Development and Implementation of Point of Care Pharmacogenetic Testing,” Expert review of Molecular Diagnostics 16, no. 9 (2016): 949–960. 27402403 10.1080/14737159.2016.1211934 PMC6709578

[252] B. T. Velayudhan and H. K. Naikare , “Point‐of‐care Testing in Companion and Food Animal Disease Diagnostics,” Frontiers in Veterinary Science 9 (2022): 1056440. 36504865 10.3389/fvets.2022.1056440 PMC9732271

[253] S. Choi , “Powering Point‐of‐care Diagnostic Devices,” Biotechnology Advances 34, no. 3 (2016): 321–330. 26631766 10.1016/j.biotechadv.2015.11.004

[254] G. A. Aleku , M. P. Adoga , and S. M. Agwale , “HIV Point‐of‐care Diagnostics: Meeting the Special Needs of sub‐Saharan Africa,” Journal of Infection in Developing Countries 8, no. 10 (2014): 1231–1243. 25313598 10.3855/jidc.4664

[255] E. M. Abdel‐Moety , A. M. Abou Al‐Alamein , and E. Fawaz , “A Companion Diagnostic for Personalizing Mivacurium at the Point‐of‐Care,” Journal of the Electrochemical Society 167, no. 8 (2020): 087510.

[256] J. Aidoo‐Brown , D. Moschou , and P. Estrela , “Multiplexed Prostate Cancer Companion Diagnostic Devices,” Sensors (Basel, Switzerland) 21, no. 15 (2021): 5023. 34372259 10.3390/s21155023 PMC8347987

[257] A. S. Modak , “Point‐of‐care Companion Diagnostic Tests for Personalizing Psychiatric Medications: Fulfilling an Unmet Clinical Need,” Journal of Breath Research 12, no. 1 (2017): 017101. 28920579 10.1088/1752-7163/aa8d2e

[258] A. Panneer Selvam and S. Prasad , “Companion and Point‐of‐Care Sensor System for Rapid Multiplexed Detection of a Panel of Infectious Disease Markers,” SLAS Technology 22, no. 3 (2017): 338–347. 28520525 10.1177/2211068217696779

[259] A. Guan , Y. Wang , K. S. Phillips , et al., “A Contact‐lens‐on‐a‐chip Companion Diagnostic Tool for Personalized Medicine,” Lab on a Chip 16, no. 7 (2016): 1152–1156. 26923038 10.1039/c6lc00034g

[260] A. L. Vataire , S. Aballéa , and G. Katz , “Economic Impact of a Genomic Companion Diagnostic Test for Breast Cancer Patients in French Private Hospitals,” Value in Health: The Journal of the International Society for Pharmacoeconomics and Outcomes Research 17, no. 7 (2014): A623. 10.1016/j.jval.2014.08.2214 27202199

[261] G. Campbell , “The Role of Statistics in the Design and Analysis of Companion Diagnostic (CDx) Studies,” Biostatistics & Epidemiology 5, no. 2 (2021): 218–231.

[262] Y. Liu , T. Szu‐Yu , M. Michael , et al., “Thresholding of a Continuous Companion Diagnostic Test Confident of Efficacy in Targeted Population,” Statistics in Biopharmaceutical Research 8, no. 3 (2016): 325–333.

[263] Y. Li , Y. Gu , and J. Jiang , “Analysis of the Relationship between the Quality of Small Biopsy Specimens of Non‐small Cell Lung Cancer and the Mutation Rate of EGFR Gene,” Zhongguo Fei Ai Za Zhi 24, no. 5 (2021): 331–337. 34034456 10.3779/j.issn.1009-3419.2021.102.16 PMC8174110

[264] S. A. Turner , J. D. Peterson , J. R. Pettus , et al., “The Pitfalls of Companion Diagnostics: Evaluation of Discordant EGFR Mutation Results From a Clinical Laboratory and a Central Laboratory,” The Journal of Molecular Diagnostics: JMD 18, no. 3 (2016): 331–335. 26923179 10.1016/j.jmoldx.2015.12.004 PMC5707184

[265] H. Moulahoum and F. Ghorbanizamani , “The LOD Paradox: When Lower Isn't Always Better in Biosensor Research and Development,” Biosensors and Bioelectronics 264 (2024): 116670. 39151260 10.1016/j.bios.2024.116670

[266] C. Chen , M. P. Douglas , M. V. Ragavan , et al., “Clinical Validity and Utility of Circulating Tumor DNA (ctDNA) Testing in Advanced Non‐small Cell Lung Cancer (aNSCLC): A Systematic Literature Review and Meta‐analysis,” Molecular Diagnosis & Therapy 28, no. 5 (2024): 525–536. 39093546 10.1007/s40291-024-00725-x PMC11349784

[267] S. Cabello‐Aguilar , J. A. Vendrell , and J. Solassol , “Real‐World Technical Hurdles of ctDNA NGS Analysis: Lessons From Clinical Implementation,” Diseases 13, no. 10 (2025): 312. 41149046 10.3390/diseases13100312 PMC12564474

[268] C. Fiala and E. P. Diamandis , “Utility of Circulating Tumor DNA in Cancer Diagnostics With Emphasis on Early Detection,” BMC Medicine [Electronic Resource] 16, no. 1 (2018): 166. 30285732 10.1186/s12916-018-1157-9 PMC6167864

[269] M. Li , “Statistical Methods for Clinical Validation of Follow‐On Companion Diagnostic Devices via an External Concordance Study,” Statistics in Biopharmaceutical Research 8, no. 3 (2016): 355–363.

[270] L. Ma , H. Guo , Y. Zhao , et al., “Liquid Biopsy in Cancer Current: Status, Challenges and Future Prospects,” Signal Transduct Target Ther 9, no. 1 (2024): 336. 39617822 10.1038/s41392-024-02021-w PMC11609310

[271] M. Gerlinger , A. J. Rowan , S. Horswell , et al., “Intratumor Heterogeneity and Branched Evolution Revealed by Multiregion Sequencing,” New England Journal of Medicine 366, no. 10 (2012): 883–892. 22397650 10.1056/NEJMoa1113205 PMC4878653

[272] J. C. M. Wan , C. Massie , J. Garcia‐Corbacho , et al., “Liquid Biopsies Come of Age: Towards Implementation of Circulating Tumour DNA,” Nature Reviews Cancer 17, no. 4 (2017): 223–238. 28233803 10.1038/nrc.2017.7

[273] G. Siravegna , S. Marsoni , S. Siena , et al., “Integrating Liquid Biopsies Into the Management of Cancer,” Nature Reviews Clinical Oncology 14, no. 9 (2017): 531–548. 10.1038/nrclinonc.2017.14 28252003

[274] C. Alix‐Panabières and K. Pantel , “Clinical Prospects of Liquid Biopsies,” Nature Biomedical Engineering 1, no. 4 (2017): 0065.

[275] N. McGranahan and C. Swanton , “Clonal Heterogeneity and Tumor Evolution: Past, Present, and the Future,” Cell 168, no. 4 (2017): 613–628. 28187284 10.1016/j.cell.2017.01.018

[276] I. Dagogo‐Jack and A. T. Shaw , “Tumour Heterogeneity and Resistance to Cancer Therapies,” Nature Reviews Clinical Oncology 15, no. 2 (2018): 81–94. 10.1038/nrclinonc.2017.166 29115304

[277] J. F. Graf and M. I. Zavodszky , “Characterizing the Heterogeneity of Tumor Tissues From Spatially Resolved Molecular Measures,” PLoS ONE 12, no. 11 (2017): e0188878. 29190747 10.1371/journal.pone.0188878 PMC5708750

[278] P. R. Prasetyanti and J. P. Medema , “Intra‐tumor Heterogeneity From a Cancer Stem Cell Perspective,” Molecular Cancer 16, no. 1 (2017): 41. 28209166 10.1186/s12943-017-0600-4 PMC5314464

[279] J. Hausser and U. Alon , “Tumour Heterogeneity and the Evolutionary Trade‐offs of Cancer,” Nature Reviews Cancer 20, no. 4 (2020): 247–257. 32094544 10.1038/s41568-020-0241-6

[280] C. Carmona‐Fontaine , M. Deforet , L. Akkari , et al., “Metabolic Origins of Spatial Organization in the Tumor Microenvironment,” PNAS 114, no. 11 (2017): 2934–2939. 28246332 10.1073/pnas.1700600114 PMC5358370

[281] E. F. Davis‐Marcisak , T. D. Sherman , P. Orugunta , et al., “Differential Variation Analysis Enables Detection of Tumor Heterogeneity Using Single‐Cell RNA‐Sequencing Data,” Cancer Research 79, no. 19 (2019): 5102–5112. 31337651 10.1158/0008-5472.CAN-18-3882 PMC6844448

[282] Y. N. Lamb , “Osimertinib: A Review in Previously Untreated, EGFR Mutation‐Positive, Advanced NSCLC,” Target Oncol 16, no. 5 (2021): 687–695. 34564820 10.1007/s11523-021-00839-w PMC8536603

[283] J. He , Z. Huang , L. Han , et al., “Mechanisms and Management of 3rd‑Generation EGFR‑TKI Resistance in Advanced Non‑Small Cell Lung Cancer (Review),” International Journal of Oncology 59, no. 5 (2021): 90. 34558640 10.3892/ijo.2021.5270 PMC8562388

[284] S. Morganti , P. Tarantino , E. Ferraro , et al., “Complexity of Genome Sequencing and Reporting: Next Generation Sequencing (NGS) Technologies and Implementation of Precision Medicine in Real Life,” Critical Reviews in Oncology/Hematology 133 (2019): 171–182. 30661654 10.1016/j.critrevonc.2018.11.008

[285] K. Mishiro , S. Imai , Y. Ematsu , et al., “RGD Peptide‐Conjugated Dodecaborate With the Ga‐DOTA Complex: A Preliminary Study for the Development of Theranostic Agents for Boron Neutron Capture Therapy and Its Companion Diagnostics,” Journal of Medicinal Chemistry 65, no. 24 (2022): 16741–16753. 36512639 10.1021/acs.jmedchem.2c01586

[286] D. Luo , J. A. Smith , N. A. Meadows , et al., “A Quantitative Assessment of Factors Affecting the Technological Development and Adoption of Companion Diagnostics,” Frontiers in Genetics 6 (2015): 357. 26858745 10.3389/fgene.2015.00357 PMC4730156

[287] M. Gomez Montero , H. El Alili , and M. Hashim , “How Are Companion Diagnostics Considered in Economic Evaluations of Oncology Treatments? A Review of Health Technology Assessments,” PharmacoEconomics—Open 6, no. 5 (2022): 637–646. 35790681 10.1007/s41669-022-00350-6 PMC9440183

[288] S. B. Kim , I. H. Song , Y. S. Song , et al., “Biodistribution and Internal Radiation Dosimetry of a Companion Diagnostic Radiopharmaceutical, [^68^Ga]PSMA‐11, in Subcutaneous Prostate Cancer Xenograft Model Mice,” Scientific Reports 11, no. 1 (2021): 15263. 34315965 10.1038/s41598-021-94684-6 PMC8316415

[289] B. Doble , M. Tan , A. Harris , et al., “Modeling Companion Diagnostics in Economic Evaluations of Targeted Oncology Therapies: Systematic Review and Methodological Checklist,” Expert Review of Molecular Diagnostics 15, no. 2 (2015): 235–254. 25142227 10.1586/14737159.2014.929499

[290] T. A. Barnes , E. Amir , A. J. Templeton , et al., “Efficacy, Safety, Tolerability and Price of Newly Approved Drugs in Solid Tumors,” Cancer Treatment Reviews 56 (2017): 1–7. 28437678 10.1016/j.ctrv.2017.03.011

[291] J. R. Trosman , S. L. Van Bebber , and K. A. Phillips , “Coverage Policy Development for Personalized Medicine: Private Payer Perspectives on Developing Policy for the 21‐gene Assay,” J Oncol Pract 6, no. 5 (2010): 238–242. 21197187 10.1200/JOP.000075 PMC2936466

[292] A. Aggarwal , T. Fojo , C. Chamberlain , et al., “Do Patient Access Schemes for High‐cost Cancer Drugs Deliver Value to Society?‐Lessons From the NHS Cancer Drugs Fund,” Annals of Oncology: Official Journal of the European Society for Medical Oncology 28, no. 8 (2017): 1738–1750. 28453615 10.1093/annonc/mdx110 PMC5834015

[293] L. Govaerts , S. Simoens , W. Van Dyck , et al., “Shedding Light on Reimbursement Policies of Companion Diagnostics in European Countries,” Value in Health: The Journal of the International Society for Pharmacoeconomics and Outcomes Research 23, no. 5 (2020): 606–615. 32389226 10.1016/j.jval.2020.01.013

[294] M. R. Trusheim and E. R. Berndt , “The Clinical Benefits, Ethics, and Economics of Stratified Medicine and Companion Diagnostics,” Drug Discovery Today 20, no. 12 (2015): 1439–1450. 26542060 10.1016/j.drudis.2015.10.017

[295] B. Quinn , “Payers and the Assessment of Clinical Utility for Companion Diagnostics,” Clinical Pharmacology and Therapeutics 88, no. 6 (2010): 751–754. 21081944 10.1038/clpt.2010.234

[296] S. K. Byron , N. Crabb , E. George , et al., “The Health Technology Assessment of Companion Diagnostics: Experience of NICE,” Clinical Cancer Research: An Official Journal of the American Association for Cancer Research 20, no. 6 (2014): 1469–1476. 24634470 10.1158/1078-0432.CCR-13-1955

[297] L. San Miguel and F. Hulstaert , “The Importance of Test Accuracy in Economic Evaluations of Companion Diagnostics,” Journal of Comparative Effectiveness Research 4, no. 6 (2015): 569–577. 26529499 10.2217/cer.15.41

[298] L. Schluckebier , R. Caetano , O. U. Garay , et al., “Cost‐effectiveness Analysis Comparing Companion Diagnostic Tests for EGFR, ALK, and ROS1 versus Next‐generation Sequencing (NGS) in Advanced Adenocarcinoma Lung Cancer Patients,” BMC Cancer 20, no. 1 (2020): 875. 32928143 10.1186/s12885-020-07240-2 PMC7489015

[299] E. A. Lim , H. Lee , E. Bae , et al., “Economic Evaluation of Companion Diagnostic Testing for EGFR Mutations and First‐Line Targeted Therapy in Advanced Non‐Small Cell Lung Cancer Patients in South Korea,” PLoS ONE 11, no. 8 (2016): e0160155. 27483001 10.1371/journal.pone.0160155 PMC4970739

[300] G. S. Zaric , “Cost Implications of Value‐Based Pricing for Companion Diagnostic Tests in Precision Medicine,” Pharmacoeconomics 34, no. 7 (2016): 635–644. 26899833 10.1007/s40273-016-0388-x

[301] D. Horgan , G. Ciliberto , P. Conte , et al., “Bringing Greater Accuracy to Europe's Healthcare Systems: The Unexploited Potential of Biomarker Testing in Oncology,” Biomedicine Hub 5, no. 3 (2020): 182–223. 33564664 10.1159/000511209 PMC7841733

[302] J. Lee and F. Lopez‐Rios , “Health Economics and Outcomes Research: Informing Companion Diagnostic Development, Guidelines, Adoption and Reimbursement,” Expert Review of Molecular Diagnostics 13, no. 5 (2013): 413–415. 23782247 10.1586/erm.13.27

[303] D. F. Steiner , P. C. Chen , and C. H. Mermel , “Closing the Translation Gap: AI Applications in Digital Pathology,” Biochimica et Biophysica Acta Reviews on Cancer 1875, no. 1 (2021): 188452. 33065195 10.1016/j.bbcan.2020.188452

[304] R. Kamla and N. Komori , “Diagnosing the Translation Gap,” Accounting, Auditing & Accountability Journal 31, no. 7 (2018): 1874–1903.

[305] D. Hartl , V. de Luca , A. Kostikova , et al., “Translational Precision Medicine: An Industry Perspective,” Journal of Translational Medicine 19, no. 1 (2021): 245. 34090480 10.1186/s12967-021-02910-6 PMC8179706

[306] H. P. Shih , X. Zhang , and A. M. Aronov , “Drug Discovery Effectiveness From the Standpoint of Therapeutic Mechanisms and Indications,” Nature Reviews Drug Discovery 17, no. 1 (2018): 78. 10.1038/nrd.2017.255 29242612

[307] G. M. Blumenthal , E. Mansfield , and R. Pazdur , “Next‐Generation Sequencing in Oncology in the Era of Precision Medicine,” JAMA Oncology 2, no. 1 (2016): 13–14. 26540172 10.1001/jamaoncol.2015.4503

[308] R. Salgado , A. M. Bellizzi , D. Rimm , et al., “How Current Assay Approval Policies Are Leading to Unintended Imprecision Medicine,” The Lancet Oncology 21, no. 11 (2020): 1399–1401. 33098760 10.1016/S1470-2045(20)30592-1

[309] D. M. Roscoe , Y. F. Hu , and R. Philip , “Companion Diagnostics: A Regulatory Perspective From the Last 5 Years of Molecular Companion Diagnostic Approvals,” Expert Review of Molecular Diagnostics 15, no. 7 (2015): 869–880. 26109316 10.1586/14737159.2015.1045490

[310] M. Ansari , “The Regulation of Companion Diagnostics: A Global Perspective,” Therapeutic Innovation & Regulatory Science 47, no. 4 (2013): 405–415. 30235516 10.1177/2168479013492734

[311] S. L. Kang , J. H. Woo , N. H. Kim , et al., “Necessity of Strengthening the Current Clinical Regulatory for Companion Diagnostics: An Institutional Comparison of the FDA, EMA, and MFDS,” Molecular Therapy Methods & Clinical Development 30 (2023): 447–458. 37663648 10.1016/j.omtm.2023.08.008 PMC10474566

[312] J. T. Jørgensen , “Oncology Drug‐companion Diagnostic Combinations,” Cancer Treatment and Research Communications 29 (2021): 100492. 34844911 10.1016/j.ctarc.2021.100492

[313] S. W. Karuri and R. Simon , “A Two‐stage Bayesian Design for Co‐development of New Drugs and Companion Diagnostics,” Statistics in Medicine 31, no. 10 (2012): 901–914. 22238151 10.1002/sim.4462

[314] P. D. Cotter , “A New Paradigm for Personalized Medicine and Companion Diagnostics: The Contract Diagnostics Organization,” The Open Conference Proceedings Journal 3, no. 1 (2012): 52–58.

[315] J. Thomas , E. Stratton , and M. Keppens , “Companion Diagnostics: Emerging Strategies and Issues in Pharmaceutical Development,” Expert Review of Molecular Diagnostics 12, no. 6 (2012): 561–563. 22845476 10.1586/erm.12.49

[316] S. H. Ou , R. A. Soo , A. Kubo , et al., “Will the Requirement by the US FDA to Simultaneously Co‐Develop Companion Diagnostics (CDx) Delay the Approval of Receptor Tyrosine Kinase Inhibitors for RTK‐Rearranged (ROS1‐, RET‐, AXL‐, PDGFR‐α‐, NTRK1‐) Non‐Small Cell Lung Cancer Globally?,” Frontiers in Oncology 4 (2014): 58. 24744988 10.3389/fonc.2014.00058 PMC3978317

[317] J. T. Jørgensen , “Companion Diagnostics and the Drug–Diagnostic Codevelopment Model,” Drug Development Research 73, no. 7 (2012): 390–397.

[318] J. T. Jørgensen and M. Hersom , “Clinical and Regulatory Aspects of Companion Diagnostic Development in Oncology,” Clinical Pharmacology and Therapeutics 103, no. 6 (2018): 999–1008. 29197081 10.1002/cpt.955

[319] J. A. Beaver , A. Tzou , G. M. Blumenthal , et al., “An FDA Perspective on the Regulatory Implications of Complex Signatures to Predict Response to Targeted Therapies,” Clinical Cancer Research: An Official Journal of the American Association for Cancer Research 23, no. 6 (2017): 1368–1372. 27993967 10.1158/1078-0432.CCR-16-1098 PMC5354944

[320] C. Ensinger , “Models of Partnership Between the Pharmaceutical and Diagnostics Industries Around Companion Diagnostics for Cancer and Beyond,” Expert Opinion on Medical Diagnostics 5, no. 2 (2011): 91–94. 23480583 10.1517/17530059.2011.552497

[321] R. Bijker , S. S. Merkouris , N. A. Dowling , et al., “ChatGPT for Automated Qualitative Research: Content Analysis,” Journal of Medical Internet Research 26 (2024): e59050. 39052327 10.2196/59050 PMC11310599

[322] A. K. Sah , D. S. Rao , A. M. Abbas , et al., “The Future of Diagnostics: Cutting‐edge Advances in Healthcare Technology,” Italian Journal of Medicine 19, no. 2 (2025). n.p.

[323] D. Amodio , C. Rossetti , A. Angelidou , et al., “Immune Development in Early Life (IDEaL) Longitudinal Cohort Study Protocol: Identifying Biomarkers of Vaccine Responsiveness, Respiratory Infection, and Asthma,” J Allergy Clin Immunol Glob 4, no. 4 (2025): 100517. 40727648 10.1016/j.jacig.2025.100517 PMC12302638

[324] Y. Yoshinaga , C. Daum , G. He , et al., “Genome Sequencing,” Methods in Molecular Biology 1775 (2018): 37–52. 29876807 10.1007/978-1-4939-7804-5_4

[325] S. A. Mahoney , A. K. Dey , N. Basisty , et al., “Identification and Functional Analysis of Senescent Cells in the Cardiovascular System Using Omics Approaches,” American Journal of Physiology. Heart and Circulatory Physiology 325, no. 5 (2023): H1039–H1058. 37656130 10.1152/ajpheart.00352.2023 PMC10908411

[326] R. Aebersold and M. Mann , “Mass‐spectrometric Exploration of Proteome Structure and Function,” Nature 537, no. 7620 (2016): 347–355. 27629641 10.1038/nature19949

[327] L. Cui , H. Lu , and Y. H. Lee , “Challenges and Emergent Solutions for LC‐MS/MS Based Untargeted Metabolomics in Diseases,” Mass Spectrometry Reviews 37, no. 6 (2018): 772–792. 29486047 10.1002/mas.21562

[328] A. D. Kennedy , M. J. Miller , K. Beebe , et al., “Metabolomic Profiling of Human Urine as a Screen for Multiple Inborn Errors of Metabolism,” Genetic Testing and Molecular Biomarkers 20, no. 9 (2016): 485–495. 27448163 10.1089/gtmb.2015.0291 PMC5314726

[329] V. Tolstikov , V. R. Akmaev , R. Sarangarajan , et al., “Clinical Metabolomics: A Pivotal Tool for Companion Diagnostic Development and Precision Medicine,” Expert Review of Molecular Diagnostics 17, no. 5 (2017): 411–413. 28317395 10.1080/14737159.2017.1308827

[330] W. Zhang , Z. Cai , D. Liang , et al., “Immune Cell‐Related Genes in Juvenile Idiopathic Arthritis Identified Using Transcriptomic and Single‐Cell Sequencing Data,” International Journal of Molecular Sciences 24, no. 13 (2023): 10619. 37445800 10.3390/ijms241310619 PMC10342059

[331] I. Zafar , A. Unar , N. U. Khan , et al., “Molecular Biology in the Exabyte Era: Taming the Data Deluge for Biological Revelation and Clinical Transformation,” Computational Biology and Chemistry 119 (2025): 108535. 40466336 10.1016/j.compbiolchem.2025.108535

[332] J. Zhang , W. Wang , and X. Q. Wang , “A Robust Diagnostic Model for High‐risk MASH: Integrating Clinical Parameters and Circulating Biomarkers through a Multi‐omics Approach,” Hepatology International 19, no. 4 (2025): 820–835. 40205303 10.1007/s12072-025-10792-9

[333] P. Wang , S. Liang , H. Zhang , et al., “Molecular Characterization of Breast Cancer and Multiple Primary Malignancies: The Latest Application Using Unmarked Quantitative Proteomics,” Int J Surg 111, no. 11 (2025): 7746–7760. 40694032 10.1097/JS9.0000000000002999 PMC12626462

[334] A. K. Sah , M. Afzal , R. H. Elshaikh , et al., “Innovative Strategies in the Diagnosis and Treatment of Liver Cirrhosis and Associated Syndromes,” Life (Basel) 15, no. 5 (2025): 779. 40430206 10.3390/life15050779 PMC12112768

[335] M. M. Alruwaili , F. H. Abuadas , M. Alsadi , et al., “Exploring Nurses' Awareness and Attitudes Toward Artificial Intelligence: Implications for Nursing Practice,” Digit Health 10 (2024): 20552076241271803. 39114115 10.1177/20552076241271803 PMC11304479

[336] P. Leo , A. Janowczyk , R. Elliott , et al., “Computer Extracted Gland Features From H&E Predicts Prostate Cancer Recurrence Comparably to a Genomic Companion Diagnostic Test: A Large Multi‐site Study,” NPJ Precision Oncology 5, no. 1 (2021): 35. 33941830 10.1038/s41698-021-00174-3 PMC8093226

[337] A. Babker , R. S. Suliman , A. A. M. Ghazwani , W. AlHarbi , and V. Lyashenko , “Combined Use of Edge Selection Operators in Individual Color Channels for the Analysis of Cytological Images Presented in RGB Format,” Biomedical and Pharmacology Journal 18 (2025): 121–138.

[338] M. S. Nabi , M. F. A. Fauzi , Z. U. Rehman , et al., “HER2‐IHC‐40x: A High‐resolution Histopathology Dataset for HER2 IHC Scoring in Breast Cancer,” Data Brief 62 (2025): 111922. 41143256 10.1016/j.dib.2025.111922 PMC12545830

[339] G. Ye , Z. Wei , C. Han , et al., “AI‐derived Longitudinal and Multi‐dimensional CT Classifier for Non‐small Cell Lung Cancer to Optimize Neoadjuvant Chemoimmunotherapy Decision: A Multicentre Retrospective Study,” EClinicalMedicine 89 (2025): 103551. 41127561 10.1016/j.eclinm.2025.103551 PMC12538916

[340] Roche . Roche granted FDA Breakthrough Device Designation for first AI‐driven companion diagnostic for non‐small cell lung cancer 2025, https://www.roche.com/media/releases/med‐cor‐2025‐04‐29?utm_source=chatgpt.com.

[341] J. Clough , M. Colwill , A. Poullis , et al., “Biomarkers in Inflammatory Bowel Disease: A Practical Guide,” Therap Adv Gastroenterol 17 (2024): 17562848241251600. 10.1177/17562848241251600 PMC11085009 38737913

[342] P. Wu , C. Zhang , X. Tang , et al., “Pan‐cancer Characterization of Cell‐free Immune‐related miRNA Identified as a Robust Biomarker for Cancer Diagnosis,” Molecular Cancer 23, no. 1 (2024): 31. 38347558 10.1186/s12943-023-01915-7 PMC10860228

[343] C. Ladbury , A. Amini , A. Govindarajan , et al., “Integration of Artificial Intelligence in Lung Cancer: Rise of the Machine,” Cell Reports Medicine 4, no. 2 (2023): 100933. 36738739 10.1016/j.xcrm.2023.100933 PMC9975283

[344] P. X. Wang , Y. C. Zhong , B. Duan , et al., “Exploring Morphological Heterogeneity of Circulating Tumor Cells: Machine Learning‐based Approach for Cell Identification and Prognostic Implications,” Science Bulletin (Beijing) 70, no. 13 (2025): 2070–2074. 10.1016/j.scib.2025.04.048 40348669

[345] K. Swanson , E. Wu , A. Zhang , et al., “From Patterns to Patients: Advances in Clinical Machine Learning for Cancer Diagnosis, Prognosis, and Treatment,” Cell 186, no. 8 (2023): 1772–1791. 36905928 10.1016/j.cell.2023.01.035

[346] A. K. Sah , R. H. Elshaikh , M. G. Shalabi , et al., “Role of Artificial Intelligence and Personalized Medicine in Enhancing HIV Management and Treatment Outcomes,” Life (Basel) 15, no. 5 (2025): 745. 40430173 10.3390/life15050745 PMC12112836

[347] M. Li , Y. Wu , and J. Lin , “Imaging‐guided Companion Theranostics: Ushering in a New Era of Personalized Cancer Care,” Nano Biomedicine and Engineering 17, no. 1 (2025): 3–16.

[348] G. Gebhart , L. E. Lamberts , Z. Wimana , et al., “Molecular Imaging as a Tool to Investigate Heterogeneity of Advanced HER2‐positive Breast Cancer and to Predict Patient Outcome Under Trastuzumab Emtansine (T‐DM1): The ZEPHIR Trial,” Annals of Oncology: Official Journal of the European Society for Medical Oncology 27, no. 4 (2016): 619–624. 26598545 10.1093/annonc/mdv577

[349] X. Shen , H. Zhou , X. Zhou , et al., “(68)Ga‐grazytracer PET for Noninvasive Assessment of Response to Immunotherapy in Solid Tumors and Lymphomas: A Phase 1/2 Clinical Trial,” Nature Communications 15, no. 1 (2024): 8791. 10.1038/s41467-024-53197-2 PMC11467221 39389969

[350] B. Li , H. Liu , Y. He , et al., “A , “Self‐Checking” pH/Viscosity‐Activatable NIR‐II Molecule for Real‐Time Evaluation of Photothermal Therapy Efficacy,” Angewandte Chemie (International ed in English) 61, no. 16 (2022): e202200025. 35170174 10.1002/anie.202200025

[351] R. Woodhouse , M. Li , J. Hughes , et al., “Clinical and Analytical Validation of FoundationOne Liquid CDx, a Novel 324‐Gene cfDNA‐based Comprehensive Genomic Profiling Assay for Cancers of Solid Tumor Origin,” PLoS ONE 15, no. 9 (2020): e0237802. 32976510 10.1371/journal.pone.0237802 PMC7518588

[352] S. Ando , S. Futami , K. Azuma , et al., “Synchronous Double Primary Lung Adenocarcinomas with EGFR L858R Point Mutation and MET Exon 14 Skipping Mutation,” Journal of Medical Cases 15, no. 8 (2024): 153–158. 39091578 10.14740/jmc4210 PMC11287901

[353] M. Wang , Y. Fan , M. Sun , et al., “Sunvozertinib for Patients in China With Platinum‐pretreated Locally Advanced or Metastatic Non‐small‐cell Lung Cancer and EGFR Exon 20 Insertion Mutation (WU‐KONG6): Single‐arm, Open‐label, Multicentre, Phase 2 Trial,” Lancet Respiratory Medicine 12, no. 3 (2024): 217–224. 38101437 10.1016/S2213-2600(23)00379-X

