# Supplementary material for: Companion Diagnostics in Clinical Therapy: Current Applications and Future Directions
Source: MedComm (2020). 2026 Mar 1;7(3):e70638. doi: 10.1002/mco2.70638 (PMC12950518; doi:10.1002/mco2.70638)
Supplement: Supplementary file 1 — Supporting Table 1: The major differences of CDx regulation policies between FDA, EMA, NMPA, MFDS, and PMDA. [file MCO2-7-e70638-s001.docx]

**Supplementary material**

**Companion Diagnostics in Clinical Therapy: Current Applications and Future Directions**

Yuesong Wu^1,2,#^, Rou Xue^1,#^, Xiangwen Luo^1, 4^, Jiangnan Liao^1, 2^, Zongbo Zhang^1^, Jinhai Deng^1^, Teng Liu^1,2,3*^, Xin Li^1, 2, 3, 4*^, Zhe-Sheng Chen^6*^, MingZhu Yin^1, 2, 3, 4,5*^

^1^ Clinical Research Center (CRC), Medical Pathology Center (MPC), Cancer Early Detection and Treatment Center (CEDTC) and Translational Medicine Research Center (TMRC), Chongqing University Three Gorges Hospital, Chongqing University, Wanzhou District, Chongqing 404100, China.

^2^ Chongqing Technical Innovation Center for Quality Evaluation and Identification of Authentic Medicinal Herbs, Wanzhou District, Chongqing 404100, China.

^3^ Chongqing University Three Gorges Hospital & Academy for Advanced interdisciplinary Technology, CQU - Ferenc Krausz Nobel Laureate Scientific Workstation.

^4^ School of Medicine Chongqing University, Chongqing University, Shapingba District, Chongqing 400030, China.

^5^ Institute of Advanced Interdisciplinary Studies,Chongqing University

^6^ Department of Pharmaceutical Sciences, College of Pharmacy and Health Sciences, St. John's University, Queens, NY 11439, USA.

Authors information:

Yuesong Wu: Email: maysonga@163.com, Tel: 18382447688.

Rou Xue: Email: xuerou321@163.com, Tel: 19122163551.

Xiangwen Luo: Email: xiangwenluo@stu.cqu.edu.cn, Tel: 18846149925.

Jiangnan Liao: Emai: jiangnanliao18@gmail.com; Tel: 13896484345.

Zongbo Zhang: Email: 18716119482@163.com, Tel: 18716119482.

Jinhai Deng: Email: jinhaideng_kcl@163.com, Tel: 18811771686.

Teng Liu: Email: tengliu17@cqu.edu.cn, Tel: 13638219888.

Xin Li: Email: lixin.bioinfor@cqu.edu.cn, Tel: 15561812785.

Zhe-Sheng Chen: Email: [chenz@stjohns.edu](mailto:chenz@stjohns.edu), Tel: 17189901432.

**SUPPORTING TABLE**

Table S1 The major differences of CDx regulation policies between FDA, EMA, NMPA, MFDS and PMDA.

| **Institutions (classifications)** | **Definition** | **Regulatory pathway** |
| --- | --- | --- |
| FDA  (Class II or III) | An IVD provides essential information for the safe and effective use of a corresponding drug or biological product.  a) Identify patients who are most likely to benefit from a particular therapeutic product,  b) Dentify patients likely to be at increased risk for serious side effects as a result of treatment with a particular therapeutic product,  c)Monitor response to treatment with a particular therapeutic product for the purpose of adjusting treatment to achieve improved safety or effectiveness. | Centered on PMA, with mandatory drug-CDx co-development and parallel review.  1) Q-Sub Pre-Submission Program  Analytical validation strategies; Clinical study design; Necessity and structure of bridging studies; and Alignment between the CDx and the therapeutic’s pivotal trials.  2) Analytical Validation  Accuracy, precision, repeatability, reproducibility; Limit of detection, limit of quantitation; Linearity and reportable range; Analytical specificity, cross-reactivity; interference studies, Sample stability and reagent stability, Platform comparability.  3) Clinical Validation  Prospective CDx testing within the therapeutic’s pivotal trials or Bridging studies comparing the to-be-marketed CDx to the assay used in drug trials.  4) PMA Submission  Analytical validation data, Clinical validation data, Manufacturing information and QMS documentation, Stability studies, Software validation.  5) FDA Coordinated Review and Approval  CDRH and CDER/CBER conduct a joint review to ensure alignment between the CDx performance and the drug’s intended use.  Upon approval: The CDx is referenced in the therapeutic product’s labeling, any significant modifications require a supplemental PMA (sPMA). |
| EMA  (Class C) | An IVD test that supports the safe and effective use of a specific medicinal product, by identifying patients that are suitable or unsuitable for treatment. | Based on NB and consultation procedure with medicinal product authorities.  1) Performance Evaluation  Scientific validity, Analytical performance, Clinical performance.  2) NB Conformity Assessment  QMS (ISO 13485), Review of the PER and technical documentation, Review of post-market surveillance and post-market clinical follow-up plans.  3) Consultation Procedure with Medicinal Product.  Appropriateness of the CDx for the specified medicinal product, Clinical relevance and cutoff justification, Alignment with the drug’s summary of product characteristics, this adds 60-150 days to the evaluation.  4) CE Marking  When the NB assessment and consultation procedure are both completed, the CDx can receive CE marking.  5) Post-Market Requirements  PMCF activities, PMS reporting, NB reassessment for significant design or performance changes. |
| NMPA  (High‑risk IVD, Class III) | An IVD that tests samples collected from patients with tumors, the results of which can provide important information about the safety and efficacy of antineoplastic drugs for patients.  a) Identify patients most likely to benefit from the drug;  b) Identify patients at greater risk for serious adverse reactions associated with the drug;  c) Identify subgroups of the population that have been adequately studied for safety and efficacy. | Requiring type testing, clinical trials, and coordinated review with corresponding drugs.  1) Type Testing  Analytical performance characterization; Stability studies; Safety evaluations; Software verification and validation.  2) Manufacturer Testing and Dossier Preparation  Risk management (ISO14971); Manufacturing process validation; Shelf life and transport stability; Batch-to-batch consistency.  3) Clinical Trials  Alignment with the drug’s Chinese indication; Inclusion of Chinese patient samples; Bridging studies when therapeutic trials lack Chinese populations.  4) Drug-Diagnostic Coordinated Review  Novel targeted therapies; Biomarker-defined subpopulations.  5) Approval  The CDx is typically listed in the drug’s labeling; Major post-market changes require new technical review |
| MFDS  (High‑risk IVD, Class III) | An IVD provides medical device which provides information that is essential for the safe and effective use of a corresponding therapeutic product to:  a) Identify, before and/or during treatment, patients who are most likely to benefit from the corresponding therapeutic product;  b) Identify, before and/or during treatment, patients likely to be at increased risk of serious adverse reactions as a result of treatment with the corresponding therapeutic product;  c) Monitor response to treatment with the therapeutic product for the purpose of adjusting treatment to achieve improved safety or effectiveness;  d) Identify patients in the population for whom the therapeutic product has been adequately studied, and found safe and effective, i.e., there is insufficient information about the safety and effectiveness of the therapeutic product in any other population. | PMA with Korea-specific clinical and technical expectations.  1) Pre-Consultation with MFDS  Analytical validation design; Clinical study requirements; Justification of cutoffs and intended use; Relationship between the CDx and the drug’s approved indication.  2) Analytical Validation  Accuracy, precision, repeatability, reproducibility; Analytical sensitivity and specificity; Interference and cross-reactivity; Verification across reagent lots and instruments.  3) Clinical Performance Studies  Local clinical performance data, or; Bridging studies when relying on foreign data; The clinical study must be aligned with the therapeutic’s Korean indication.  4) Technical Review  Technical dossier; Software validation; KGMP (Korean GMP) compliance; Risk management files.  5) Approval and Post-Market Changes  Significant modifications (such as changes in algorithm, cutoff, platform, or sample type) require a new review. |
| PMDA  (Highly controlled IVD) | A CoDx refers to an in vitro diagnostic agent or a medical device that is used to improve the efficacy or safety of a specific therapeutic product, is essential for using the pertinent therapeutic product, and corresponds to either of the following:  a) An in vitro diagnostic agent or a medical device that is used to identify patients who are expected to respond better to a specific therapeutic product.  b) An in vitro diagnostic agent or a medical device that is used to identify patients who are likely to be at high risk of developing adverse events associated with a particular therapeutic product.  c) An in vitro diagnostic agent or a medical device that is necessary for optimizing the treatment including dose, schedule, and discontinuation of a particular therapeutic product. | Based on Shonin approval, with mandated parallel review of the diagnostic and therapeutic.  1) Formal Pre-Consultation with PMDA  Analytical validation design; Statistical methods; Applicability to Japanese patient populations; Necessity for local bridging studies; Cutoff justification and clinical relevance.  2) Analytical Validation  Reproducibility across lots and testing sites; Interference and cross-reactivity; Robustness testing; Platform and reagent lot comparability.  3) Clinical Validation and Bridging Studies  Genetic and demographic differences; Differences in clinical practice patterns; Bridging studies may compare Japanese samples with global data to ensure clinical performance equivalence.  4) Shonin Review  Analytical and clinical evidence; Manufacturing quality under the Japanese QMS Ordinance; Co-development data with the therapeutic product. |
